# Supplementary material for: Whole-genome sequencing reveals the molecular implications of the stepwise progression of lung adenocarcinoma
Source: Nat Commun. 2023 Dec 15;14:8375. doi: 10.1038/s41467-023-43732-y (PMC10724178; doi:10.1038/s41467-023-43732-y)
Supplement: Supplementary file 1 — Supplementary Information [file 41467_2023_43732_MOESM1_ESM.pdf]

SUPPLEMENTARY INFORMATION

# Whole-genome sequencing reveals the molecular implications of the stepwise progression of lung adenocarcinoma

Yasuhiko Haga†, Yoshitaka Sakamoto†, Keiko Kajiya†, Hitomi Kawai, Miho Oka, Noriko Motoi, Masayuki Shirasawa, Masaya Yotsukura, Shun-Ichi Watanabe, Miyuki Arai, Junko Zenkoh, Kouya Shiraishi, Masahide Seki, Akinori Kanai, Yuichi Shiraishi, Yasushi Yatabe, Daisuke Matsubara, Yutaka Suzuki*, Masayuki Noguchi, Takashi Kohno*, Ayako Suzuki*

†These authors contributed equally to this work.

*To whom correspondence should be addressed;
Yutaka Suzuki; Tel.: +81 4 7136 4076; Email: ysuzuki@hgc.jp
Takashi Kohno; Tel: +81 3 3547 5272; Email: tkkohno@ncc.go.jp
Ayako Suzuki; Tel.: +81 4 7136 4076; Email: asuzuki@edu.k.u-tokyo.ac.jp

Contents:

Supplementary Figures S1–S13 (p. 2–p. 41)
Supplementary Tables S1–S10 (p. 42–p. 61)
Supplementary References (p. 62)

Supplementary Data S1, S2, and S3 are provided in a separate excel file.

## Supplementary Figures

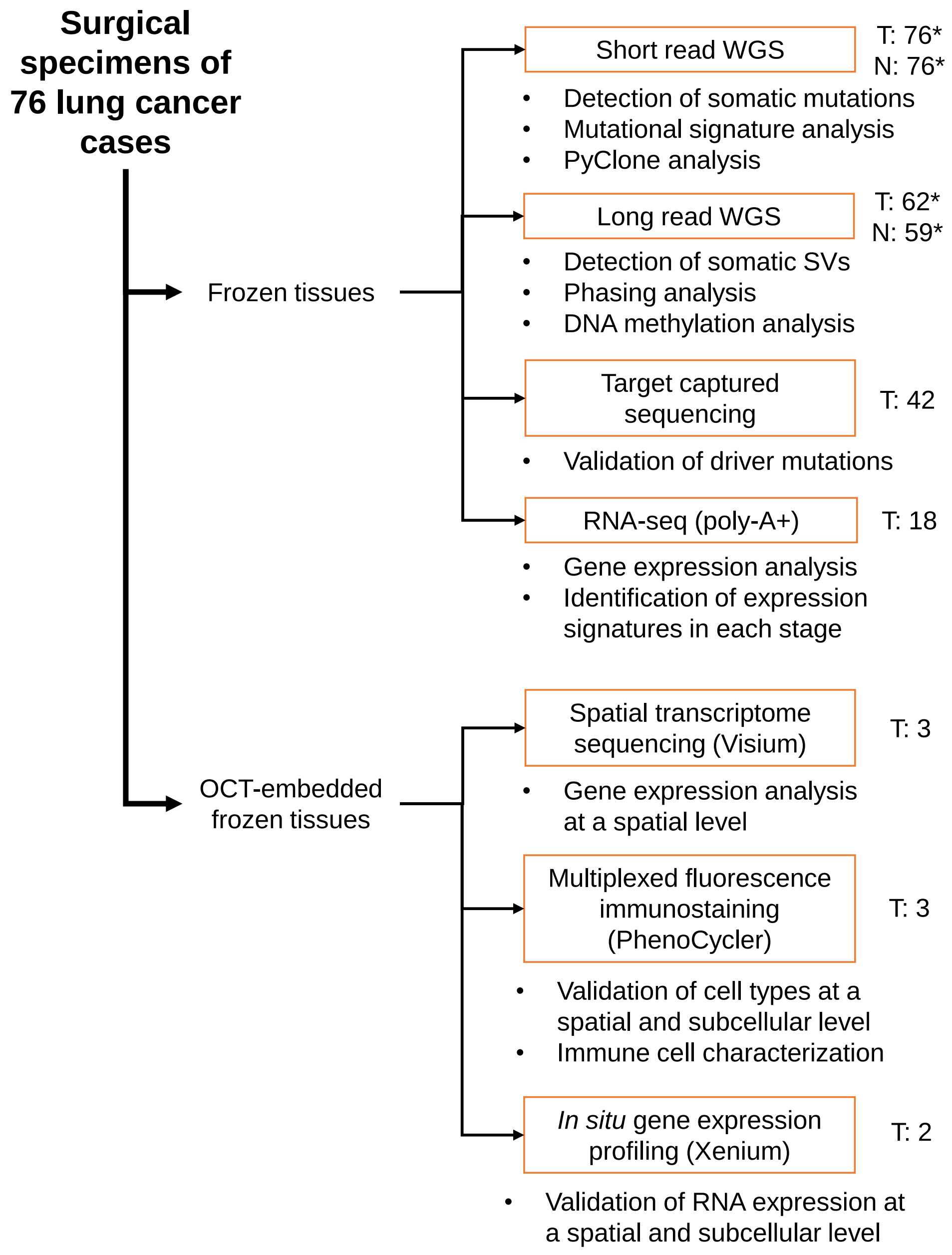


**Supplementary Figure S1 A schema of this study**

Fresh frozen tissues from surgical specimens of 76 lung cancer cases were used for this study. Short read WGS sequencing was performed for tumor-normal tissue pairs of all 76 cases. Long read sequencing was performed using high-molecular weighted DNA samples from 62 tumor and 59 normal tissues, respectively. For validation of somatic mutation detection, target captured sequencing was conducted for 42 tumor samples using the remaining DNA samples. For transcriptome analysis, RNA-seq (poly-A

method) was performed for 18 tumor samples whose RNA samples were not highly degraded. Using OCT-embedded frozen tissues, spatial omics analysis was conducted for analysis of gene expression patterns considering histological and spatial information. Spatial transcriptome sequencing analysis (Visium) and multiplexed fluorescence immunostaining (PhenoCycler) were performed for three representative Early-Ad cases. These cases harbor typical histological characters in AIS Noguchi type A, AIS Noguchi type B, and MIA Noguchi type C. For further validation of spatial expression patterns at the subcellular level, *in situ* gene expression profiling (Xenium) was performed for two AIS cases (one AIS Noguchi type A and one AIS Noguchi type B) using tissue sections nearby those used for Visium/PhenoCycler analysis. *Twenty datasets previously analyzed[1,2] were included in this study as well.

**a**

TSU-06 (AIS, Noguchi type A)
*CDKN2A* whole-gene deletion (chr9:12326709-33455460)

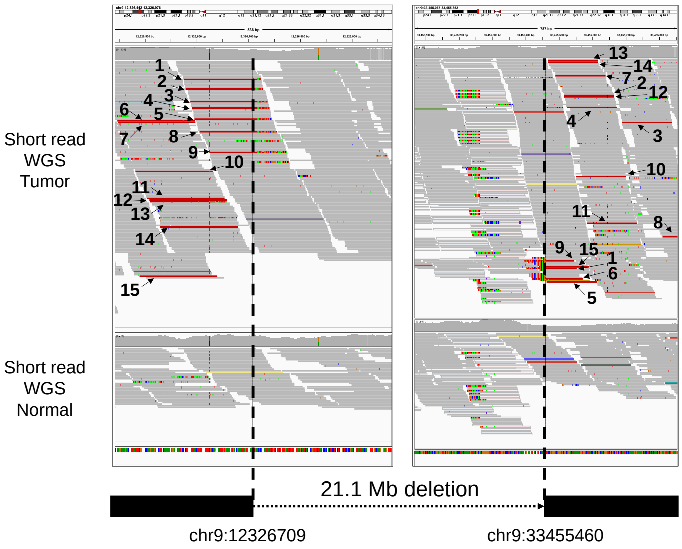


| No. | Read name | Pair orientation | Insert size |
|---|---|---|---|
| 1 | A00648:33:HFKKCDSXX:2:2302:17888:34976 | F2R1 | 21,128,978 |
| 2 | A00648:33:HFKKCDSXX:2:1631:32389:31125 | F2R1 | 21,129,085 |
| 3 | A00648:33:HFKKCDSXX:2:1347:5466:35446 | F2R1 | 21,129,250 |
| 4 | A00648:33:HFKKCDSXX:2:1649:32515:32847 | F2R1 | 21,129,082 |
| 5 | A00648:33:HFKKCDSXX:2:2557:1588:19930 | F2R1 | 21,129,017 |
| 6 | A00648:33:HFKKCDSXX:2:1543:12680:12743 | F2R1 | 21,129,124 |
| 7 | A00648:33:HFKKCDSXX:2:1348:30626:18302 | F2R1 | 21,129,191 |
| 8 | A00648:33:HFKKCDSXX:2:1139:13557:27383 | F1R2 | 21,129,354 |
| 9 | A00648:33:HFKKCDSXX:2:2454:16649:23406 | F1R2 | 21,128,921 |
| 10 | A00648:33:HFKKCDSXX:2:2574:22598:29387 | F2R1 | 21,129,218 |
| 11 | A00648:33:HFKKCDSXX:2:2608:31557:14372 | F1R2 | 21,129,230 |
| 12 | A00648:33:HFKKCDSXX:2:2547:16387:35133 | F1R2 | 21,129,156 |
| 13 | A00648:33:HFKKCDSXX:2:2631:28682:14591 | F2R1 | 21,129,107 |
| 14 | A00648:33:HFKKCDSXX:2:2403:31693:11068 | F2R1 | 21,129,087 |
| 15 | A00648:33:HFKKCDSXX:2:1536:14344:24956 | F1R2 | 21,129,100 |

**b**

TSU-06 (AIS, Noguchi type A)
*CDKN2A* whole-gene deletion (chr9:12326709-33455460)

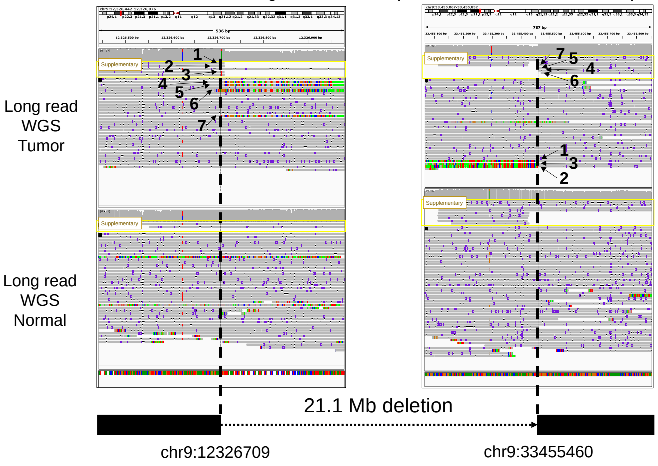


| No. | Read name |
|---|---|
| 1 | 94a52167-6c14-4faf-b5c4-8132dccf5c84 |
| 2 | 1742fefe-ce33-465c-b7c8-fcf0373f8257 |
| 3 | 506cbec3-55a2-4d35-b024-b1f0aab13ea9 |
| 4 | 9530bdc1-27b7-4770-bd0b-0d42312218d7 |
| 5 | 66c27a0d-4512-498e-8516-89c663f8fa78 |
| 6 | 0e7b345e-39bd-4efa-bc9e-fb0a27199254 |
| 7 | 3e1149cc-460e-4610-8d08-5385a9a85ef1 |

**c**

TSU-06 (AIS, Noguchi type A)

*CDKN2A* whole-gene deletion (chr9:16509949-29171657)

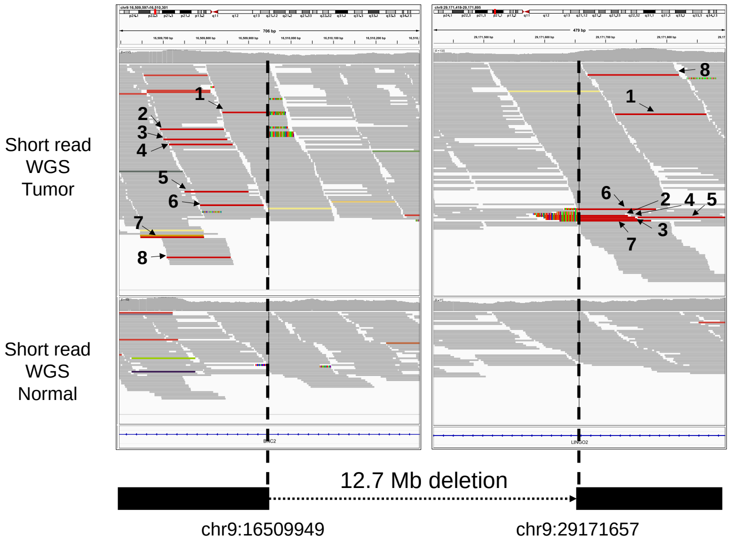


| No. | Read name | Pair orientation | Insert size |
|---|---|---|---|
| 1 | A00648:33:HFKKCDSXX:2:1161:15763:33051 | F2R1 | 12,662,027 |
| 2 | A00648:33:HFKKCDSXX:2:2625:20880:31234 | F2R1 | 12,662,044 |
| 3 | A00648:33:HFKKCDSXX:2:2328:11776:24361 | F1R2 | 12,662,051 |
| 4 | A00648:33:HFKKCDSXX:2:1646:5348:20995 | F2R1 | 12,662,035 |
| 5 | A00648:33:HFKKCDSXX:2:1318:30481:8563 | F1R2 | 12,662,152 |
| 6 | A00648:33:HFKKCDSXX:2:1443:14073:35916 | F1R2 | 12,661,996 |
| 7 | A00648:33:HFKKCDSXX:2:2632:8901:7764 | F1R2 | 12,662,128 |
| 8 | A00648:33:HFKKCDSXX:2:1473:12500:27774 | F2R1 | 12,662,112 |

**d**

TSU-06 (AIS, Noguchi type A)

*CDKN2A* whole-gene deletion (chr9:16509949-29171657)

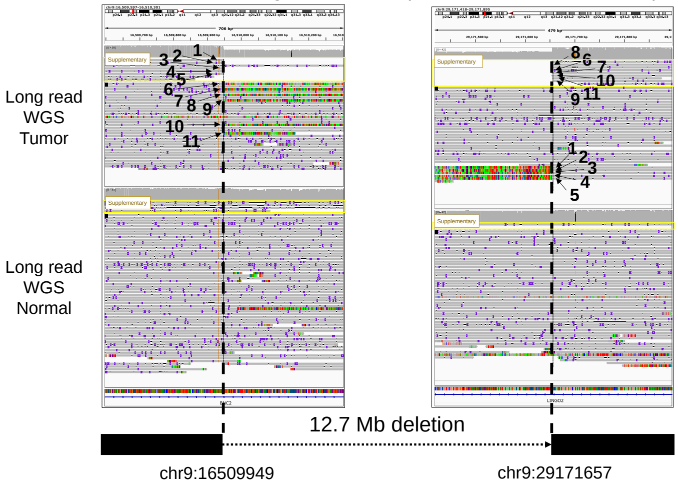


| No. | Read name |
|---|---|
| 1 | 8d7ab56b-04a2-4ab6-85ba-33273a0918bb |
| 2 | 74a80537-5b6f-412c-91d8-14af2e95e621 |
| 3 | 582832c4-66e7-4fa0-9bbc-05ae3cb0aa6e |
| 4 | 023837dd-6610-4ee6-acd9-b1476468ed31 |
| 5 | 9893d452-db8f-45b4-885e-a28ce2a7eb8e |
| 6 | 154ab6e9-fb46-4636-a2fb-fa501d822cec |
| 7 | 5f335b27-1ae0-4d13-a332-8f8cbb0ed880 |
| 8 | dd012ea3-f602-4d96-bd70-46ee5ba83551 |
| 9 | 51f1b926-62c5-4ad3-acb2-d924b158e768 |
| 10 | 8e65c7cb-2e90-4076-a7f0-18110e9149c1 |
| 11 | fb90caae-5858-456d-9fae-a7a0c64d6788 |

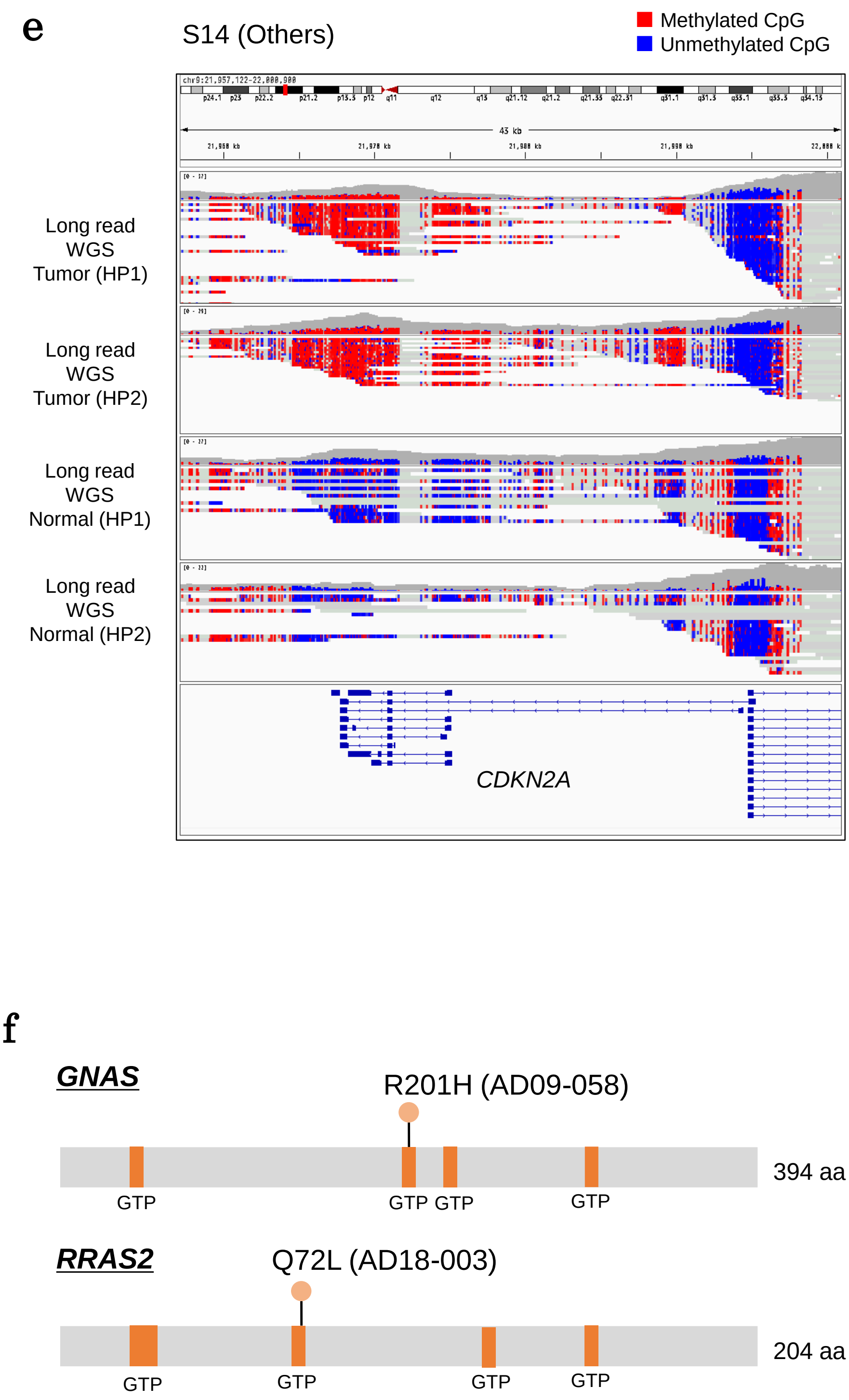
e
S14 (Others)
Methylated CpG
Unmethylated CpG
Long read WGS Tumor (HP1)
Long read WGS Tumor (HP2)
Long read WGS Normal (HP1)
Long read WGS Normal (HP2)
CDKN2A
f
GNAS
R201H (AD09-058)
394 aa
GTP
GTP GTP
GTP
RRAS2
Q72L (AD18-003)
204 aa
GTP
GTP
GTP
GTP

**g**

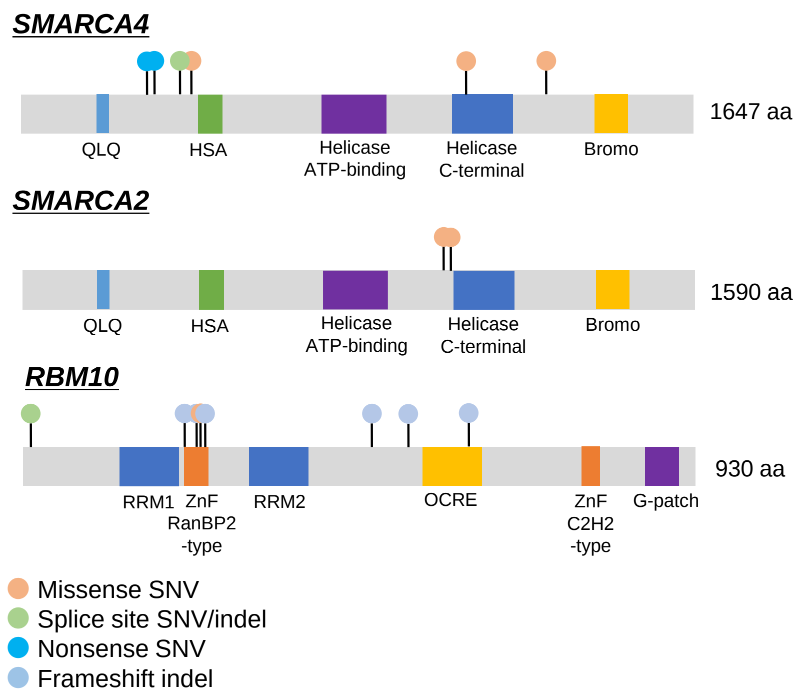


**Supplementary Figure S2 Mutations in cancer-related genes and potential driver genes** **(a–d)** Whole-gene deletions including the *CDKN2A* gene. Two representative deletions in case TSU-06 are shown. The junction points of the deletion were detected in both short and long read WGS data. Reads mapped to the surrounding region of the breakpoint are visualized by IGV. For short reads (**a c**), discordant read pairs (with abnormal insert size or pair orientation) are colored in IGV. The names of reads split mapped to both sides of the breakpoint are provided in the bottom table and the corresponding read numbers are shown with arrows on the IGV screenshot. For long reads (**b d**), the supplementary alignments and soft-clipped bases are shown as well. The names of reads splitting by the breakpoint are shown in the bottom table and the corresponding read numbers with arrows on the IGV screenshot. (**e**) Visualization of *CDKN2A* hypermethylation in case S14. The long reads in each haplotype (HP1: haplotype #1; HP2; haplotype #2) are provided with the bisulfite mode (CG) in IGV. In this case, both alleles were methylated in the *CDKN2A*/p16 promoter. (**f, g**) Non-synonymous mutations in potential driver genes (**f**) and cancer-related genes (**g**). The information on functional regions and protein domains was extracted from the UniProt database. GTP: GTP binding region; QLQ: QLQ (Gln-Leu-Gln) domain; HSA: Helicase/SANT-associated domain; Bromo: Bromodomain; RRM: RNA recognition motif domain; ZnF: zinc finger binding region; OCRE: Octamer repeat domain.

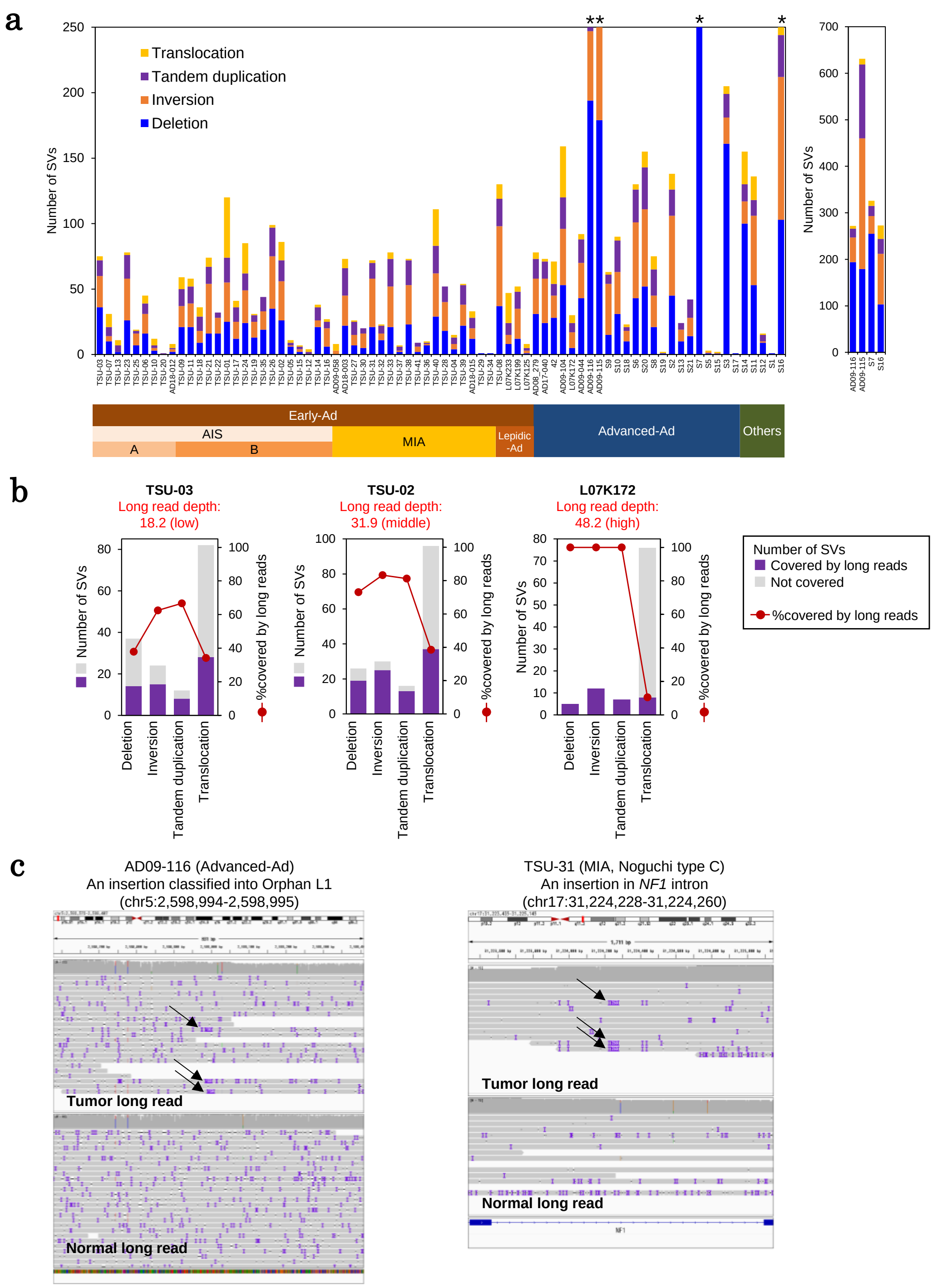


**Supplementary Figure S3 SV detection using whole-genome sequencing data**

(**a**) Number of SVs detected using short read whole-genome sequencing (WGS) data. (**b**) Number of SVs covered by long reads in each representative case. (**c**) Examples of inserted sequences. IGV visualization of long read WGS for tumor and normal samples. Source data are provided as a Source Data file for **a** and **b**.

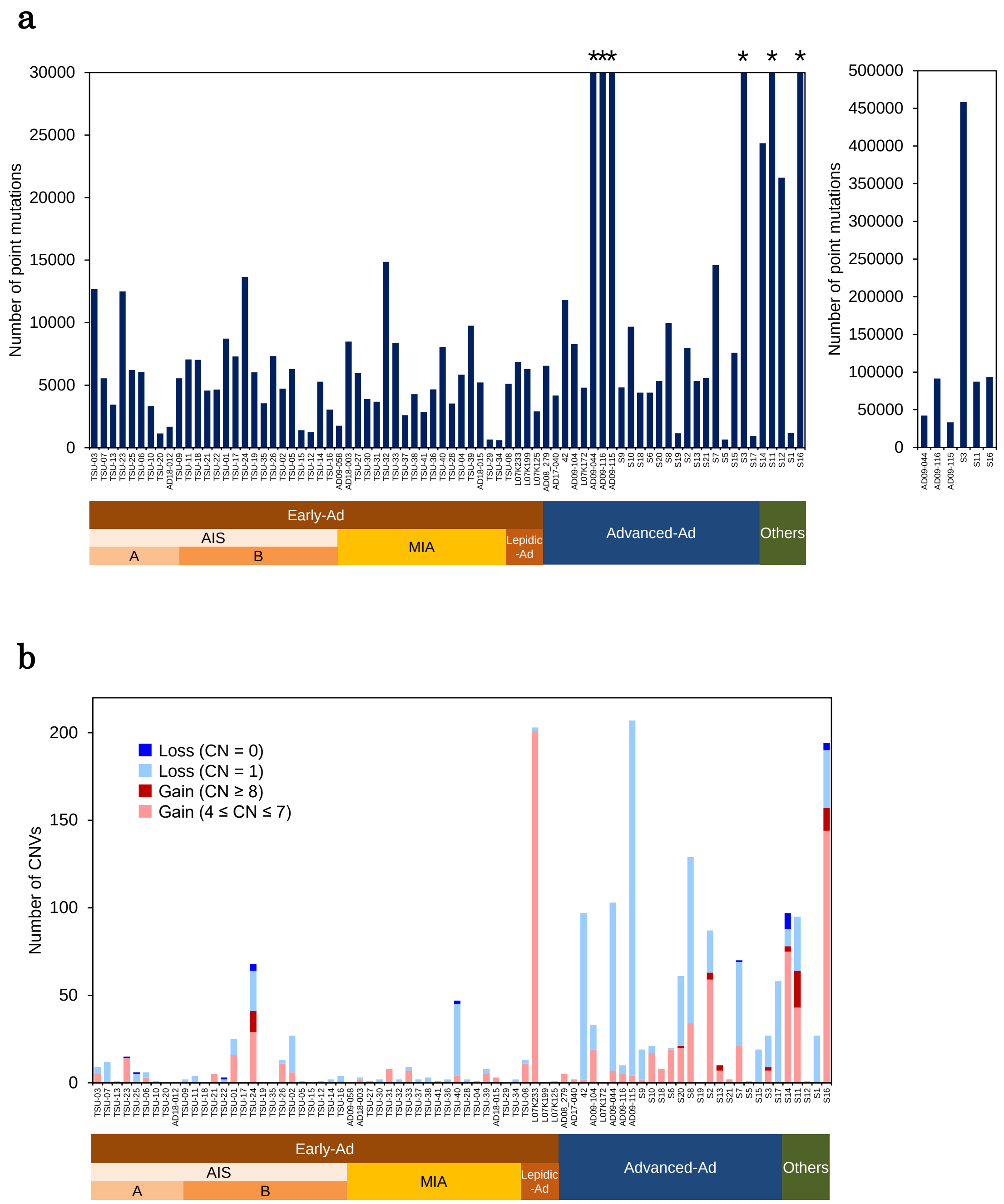
a
Number of point mutations
30000
25000
20000
15000
10000
5000
0
500000
450000
400000
350000
300000
250000
200000
150000
100000
50000
0
Number of point mutations
Early-Ad
AIS
A
B
MIA
Lepidic
-Ad
Advanced-Ad
Others
b
Number of CNVs
200
150
100
50
0
Loss (CN = 0)
Loss (CN = 1)
Gain (CN ≥ 8)
Gain (4 ≤ CN ≤ 7)
Early-Ad
AIS
A
B
MIA
Lepidic
-Ad
Advanced-Ad
Others

**c**

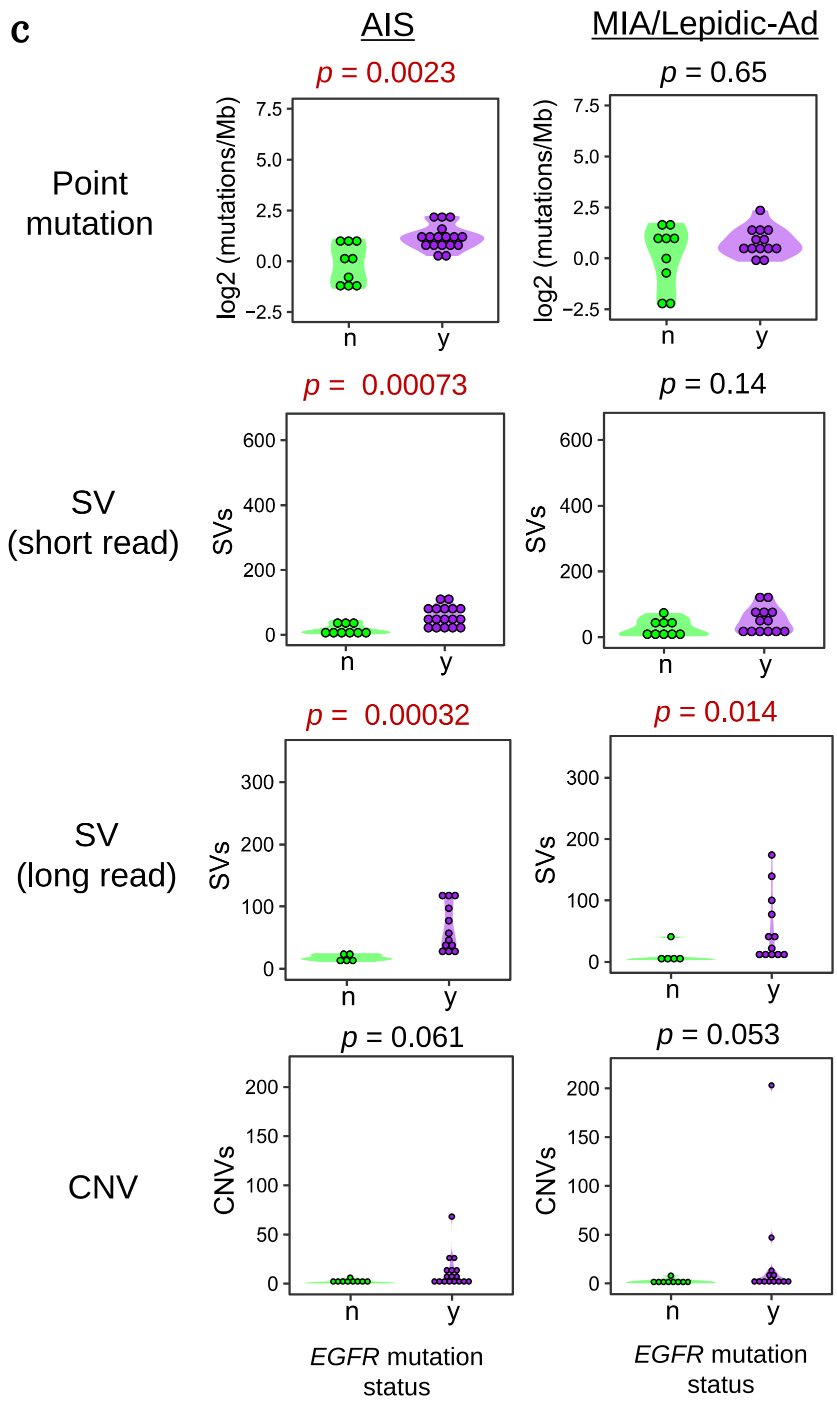
AIS
MIA/Lepidic-Ad
Point mutation
SV (short read)
SV (long read)
CNV
p = 0.0023
p = 0.65
p = 0.00073
p = 0.14
p = 0.00032
p = 0.014
p = 0.061
p = 0.053
log2 (mutations/Mb)
SVs
CNVs
n
y
EGFR mutation status

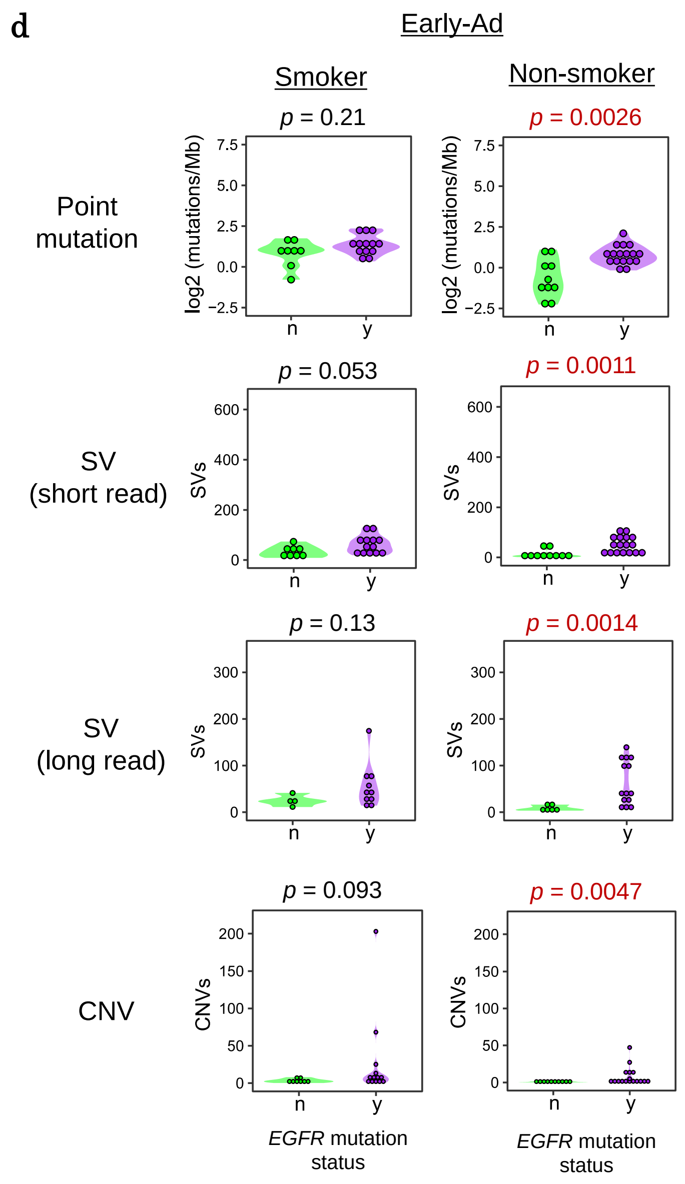


**Supplementary Figure S4 Number of somatic mutations early to advanced lung cancer cases**

(**a**) Number of point mutations in all cases. The number of mutations for the six cases with the highest numbers (asterisk) is shown in a separate graph. (**b**) Number of copy number variants (CNV) events in all cases. (**c**) Comparison of the number of mutations between *EGFR*-mutation positive and negative cases in each early adenocarcinoma subtype. The p-values were calculated by Wilcoxon rank sum test (two-sided, no multiple comparison adjustments). (**d**) Comparison of the number of mutations between *EGFR*-mutation positive and negative Early-Ad cases with/without smoking history. The p-values were calculated by Wilcoxon rank sum test (two-sided, no multiple comparison adjustments). Source data are provided as a Source Data file for **a**, **b**, **c** and **d**.

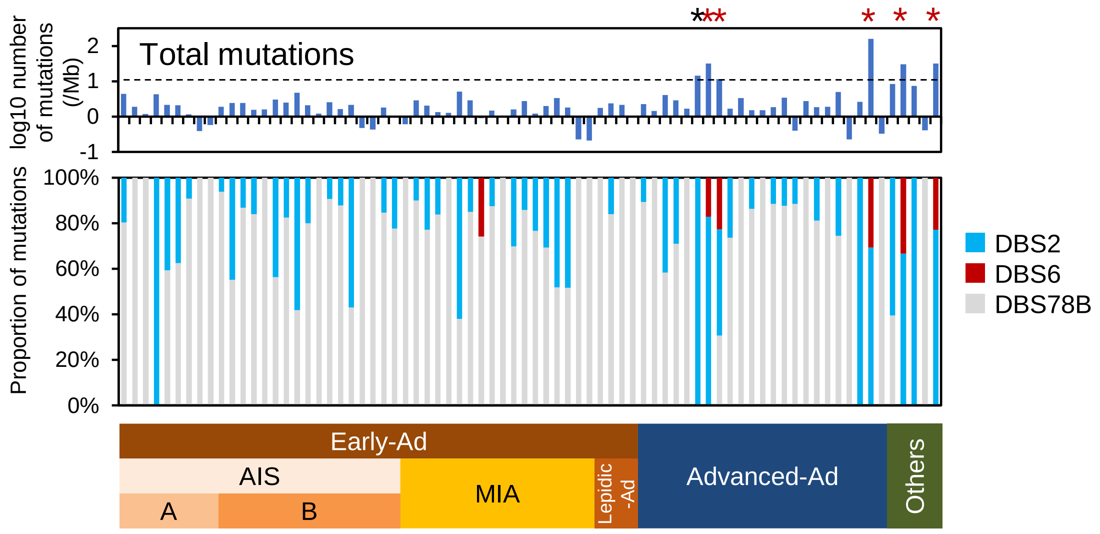


**Supplementary Figure S5 Mutational signatures of doublet nucleotide substitutions**

Proportion of doublet nucleotide substitutions (DBS) COSMIC signatures (v.3.2). Top, total number of mutations. Six cases with >10 mutations per Mb are marked with an asterisk. Among the six cases, five cases harbored mutations categorized in the DBS6 signature (indicated with a red asterisk). Source data are provided as a Source Data file.

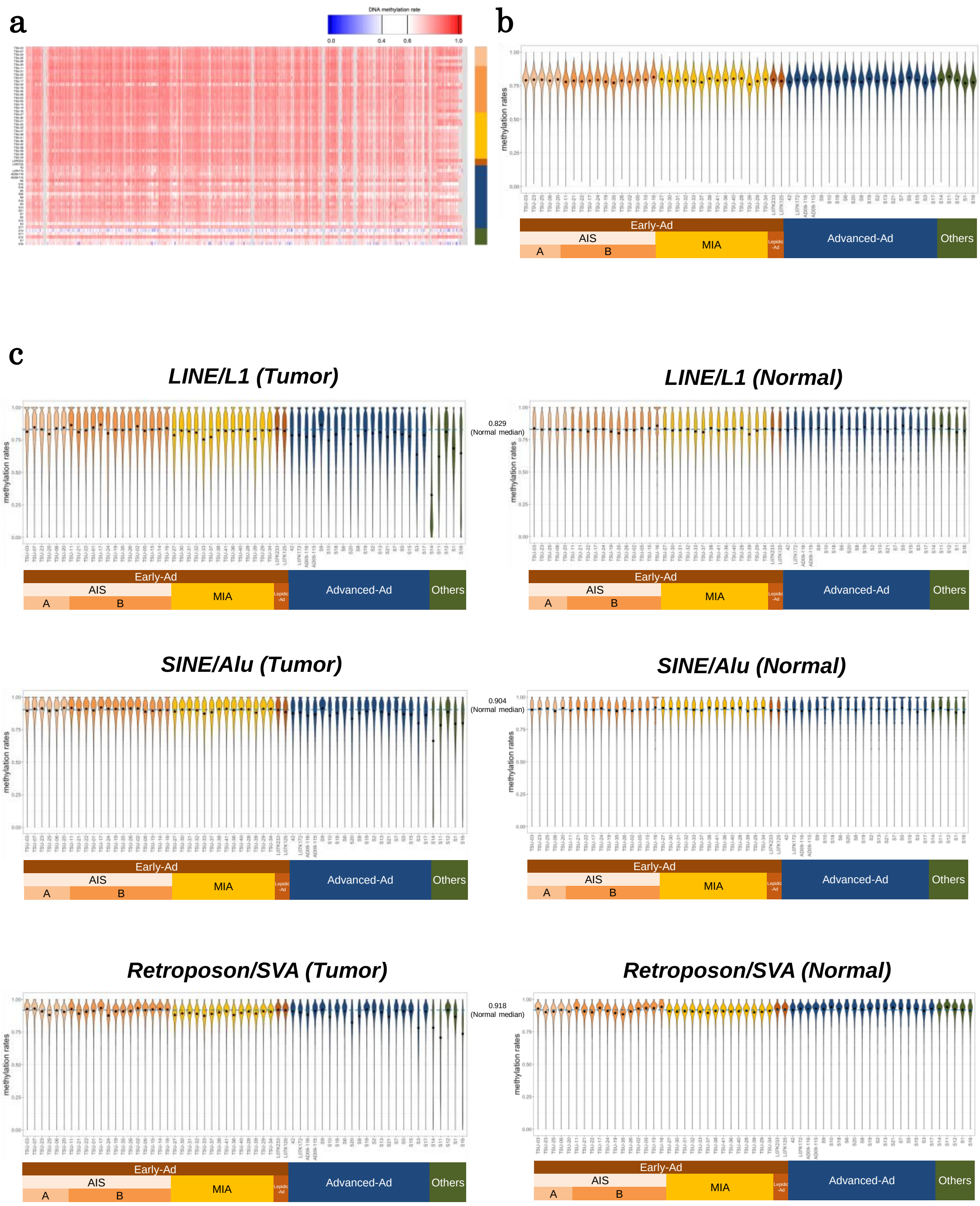
a
DNA methylation rate
0.0
0.4
0.6
1.0
b
methylation rates
Early-Ad
AIS
A
B
MIA
Lepidic-Ad
Advanced-Ad
Others
c
LINE/L1 (Tumor)
LINE/L1 (Normal)
0.829
(Normal median)
SINE/Alu (Tumor)
SINE/Alu (Normal)
0.904
(Normal median)
Retroposon/SVA (Tumor)
Retroposon/SVA (Normal)
0.918
(Normal median)

**d**

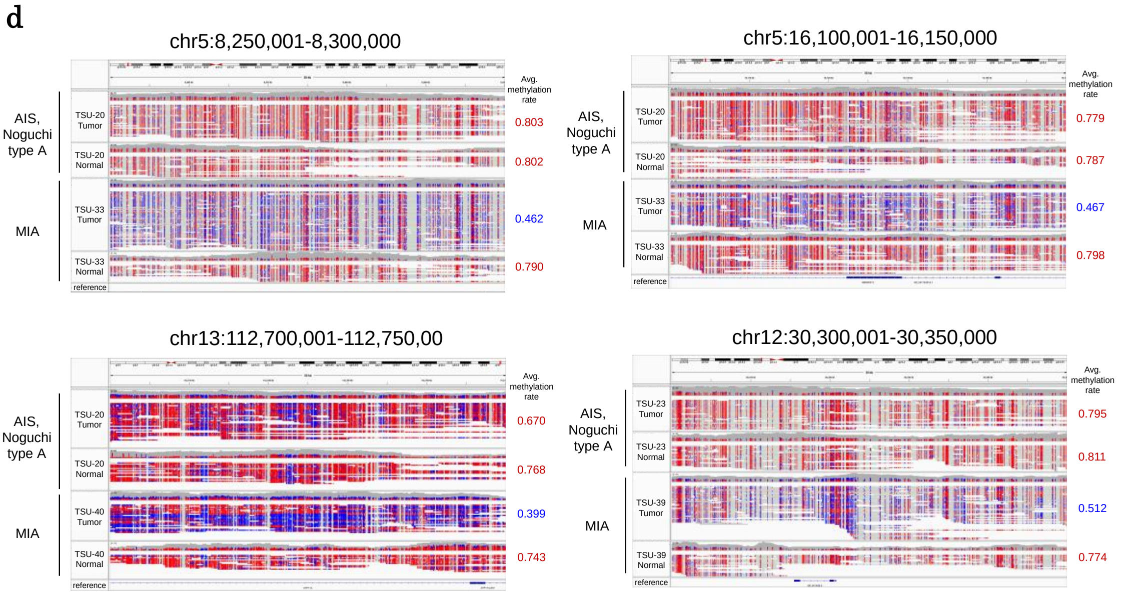
chr5:8,250,001-8,300,000
chr5:16,100,001-16,150,000
chr13:112,700,001-112,750,00
chr12:30,300,001-30,350,000
AIS, Noguchi type A
MIA
TSU-20 Tumor
TSU-20 Normal
TSU-33 Tumor
TSU-33 Normal
TSU-23 Tumor
TSU-23 Normal
TSU-39 Tumor
TSU-39 Normal
TSU-40 Tumor
TSU-40 Normal
reference
Avg. methylation rate
0.803
0.802
0.462
0.790
0.779
0.787
0.467
0.798
0.670
0.768
0.399
0.743
0.795
0.811
0.512
0.774

**e**

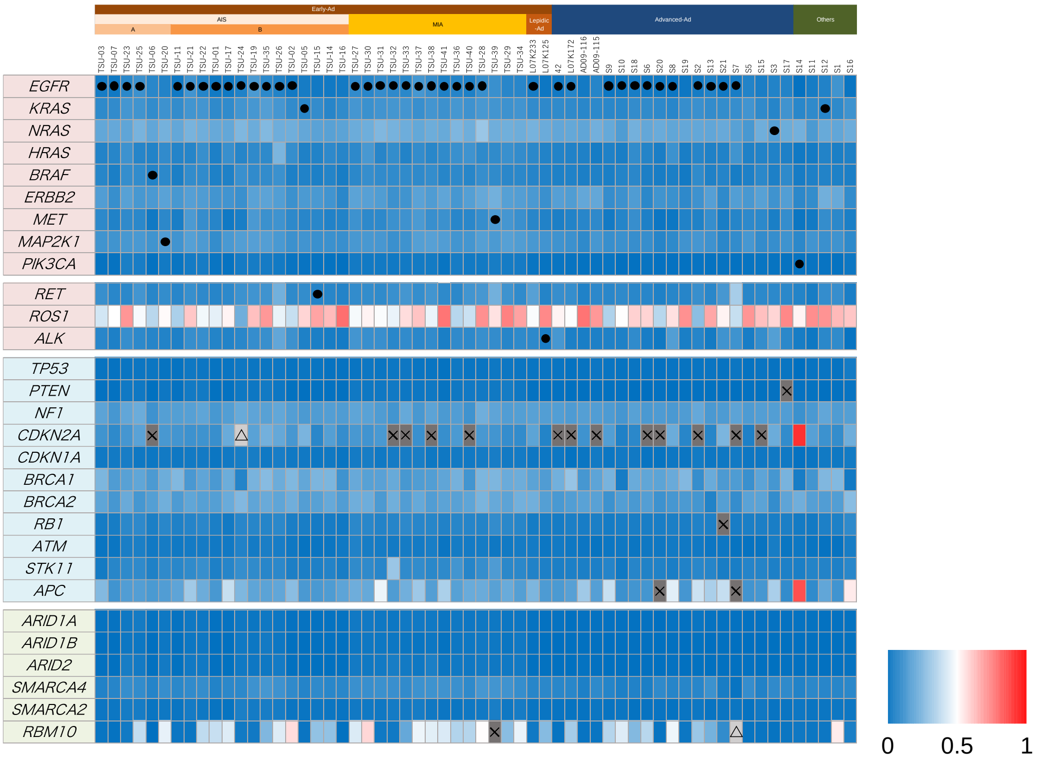
Early-Ad
AIS
A
B
MIA
Lepidic-Ad
Advanced-Ad
Others
EGFR
KRAS
NRAS
HRAS
BRAF
ERBB2
MET
MAP2K1
PIK3CA
RET
ROS1
ALK
TP53
PTEN
NF1
CDKN2A
CDKN1A
BRCA1
BRCA2
RB1
ATM
STK11
APC
ARID1A
ARID1B
ARID2
SMARCA4
SMARCA2
RBM10
0
0.5
1

f

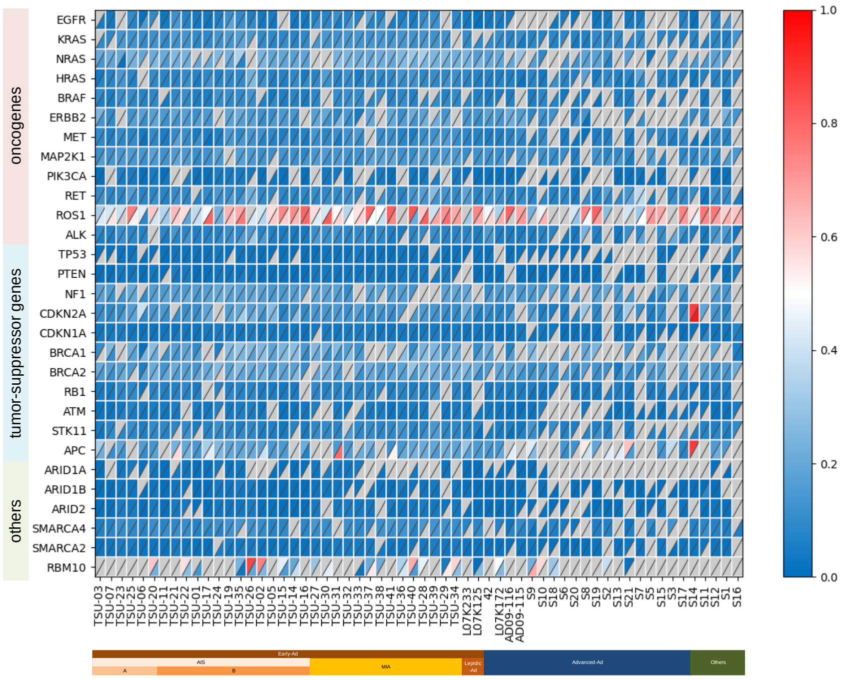
oncogenes
tumor-suppressor genes
others
EGFR
KRAS
NRAS
HRAS
BRAF
ERBB2
MET
MAP2K1
PIK3CA
RET
ROS1
ALK
TP53
PTEN
NF1
CDKN2A
CDKN1A
BRCA1
BRCA2
RB1
ATM
STK11
APC
ARID1A
ARID1B
ARID2
SMARCA4
SMARCA2
RBM10
1.0
0.8
0.6
0.4
0.2
0.0

g

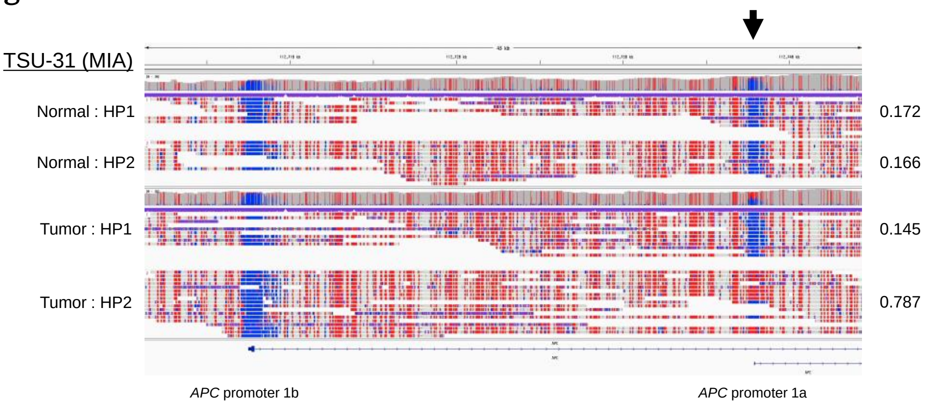
TSU-31 (MIA)
Normal : HP1
0.172
Normal : HP2
0.166
Tumor : HP1
0.145
Tumor : HP2
0.787
*APC* promoter 1b
*APC* promoter 1a

h

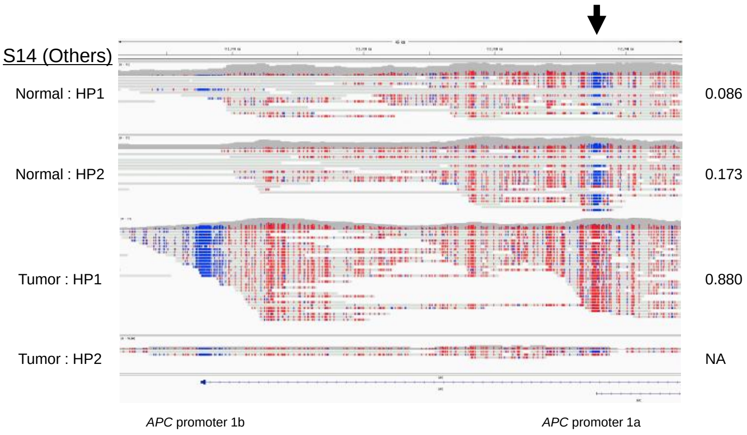
S14 (Others)
Normal : HP1
0.086
Normal : HP2
0.173
Tumor : HP1
0.880
Tumor : HP2
NA
*APC* promoter 1b
*APC* promoter 1a

**Supplementary Figure S6 DNA methylation patterns early to advanced lung cancer cases**

**(a)** Heatmap of genome-wide DNA methylation patterns (50 kb windows) for each case. **(b)** Genome-wide DNA methylation patterns (50 kb windows) of normal specimens. **(c)** DNA methylation patterns of the transposable elements, LINE-1, Alu and SVA. DNA methylation levels of tumor and normal genomes shown in the left and right panels, respectively. **(d)** Examples of hypomethylated regions in tumor genomes. IGV visualizations for the five regions. **(e)** DNA methylation rates of the promoter regions (±1.5 kb from the transcription start site, TSS) in cancer-related genes. For *BRCA1*, *BRCA2* and *APC* genes, the core promoter regions (±1 kb from the TSS) were used. Black circles (●) indicate cases with known driver mutations as defined in **Figure 1b**. Black multiplication signs (×) indicate cases harboring loss-of-function mutations. CpG sites with ≥5 reads were considered for the calculation of methylation rates. Triangle signs (△) indicate insufficient sequencing reads. **(f)** Haplotype-resolved DNA methylation patterns of promoter regions in cancer-related genes. DNA methylation rates of haplotype #1 and #2 calculated by distinguishing long reads with the HP tag. Only promoter regions with ≥50% of CpG sites covered by ≥3 reads were considered. Cases with insufficient reads are shown in grey. **(g h)** IGV visualization of DNA methylation patterns around the promoter regions of the *APC* gene.

Note:

In the analyses in **Supplementary Figures S6e–S6h**, we focused on epigenomic aberrations in cancer-related genes. Interestingly, we found that the downstream promoter region of *APC* gene was highly methylated in several Early-Ad cases. We further observed differences of DNA methylation rates between haplotype #1 and #2 at the *APC* promoter region in some cases. In an Early-Ad case (case TSU-31, MIA), one haplotype was highly methylated but the other still maintained a lower methylation state, indicating that the function of this gene would remain. On the other hand, in one advanced case (case S14, Others), hypermethylation occurred in one haplotype and LOH might occur in another haplotype, resulting in functional loss of the gene, which might start disrupting by epigenomic aberrations followed by genomic mutations during the progression of adenocarcinoma.

**a**

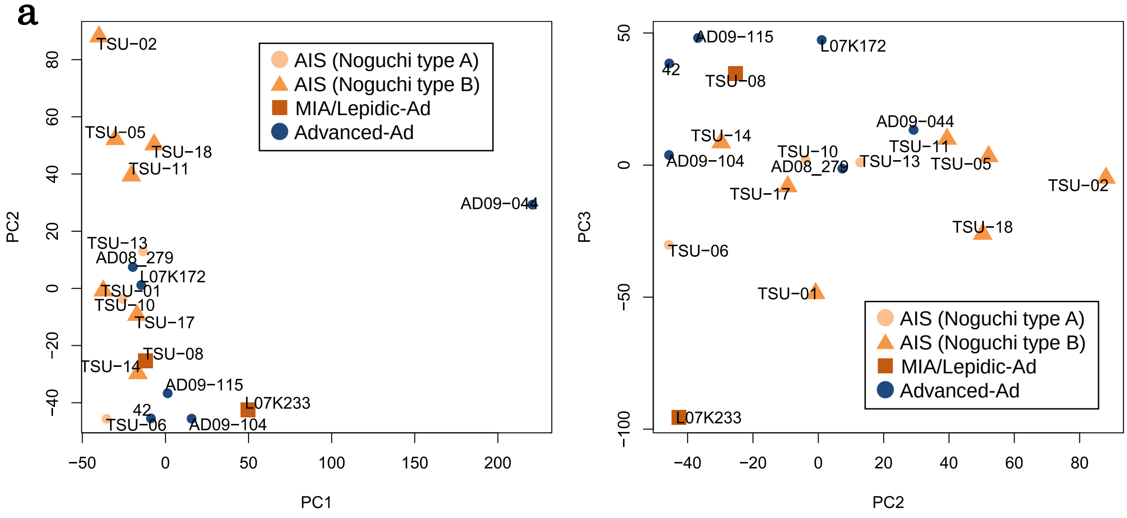

AIS (Noguchi type A)
AIS (Noguchi type B)
MIA/Lepidic-Ad
Advanced-Ad
TSU−02
TSU−05
TSU−18
TSU−11
AD09−044
TSU−13
AD08_279
L07K172
TSU−01
TSU−10
TSU−17
TSU−08
TSU−14
AD09−115
L07K233
42
TSU−06
AD09−104
TSU−03
PC1
PC2
PC3


**b**

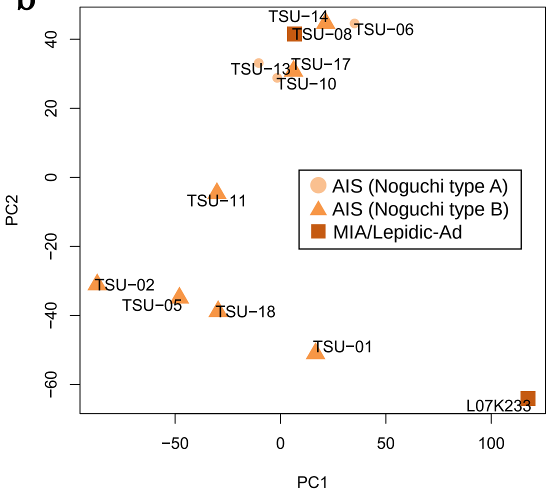

TSU−14
TSU−08
TSU−06
TSU−13
TSU−17
TSU−10
TSU−11
AIS (Noguchi type A)
AIS (Noguchi type B)
MIA/Lepidic-Ad
TSU−02
TSU−05
TSU−18
TSU−01
L07K233
PC1
PC2

**c**

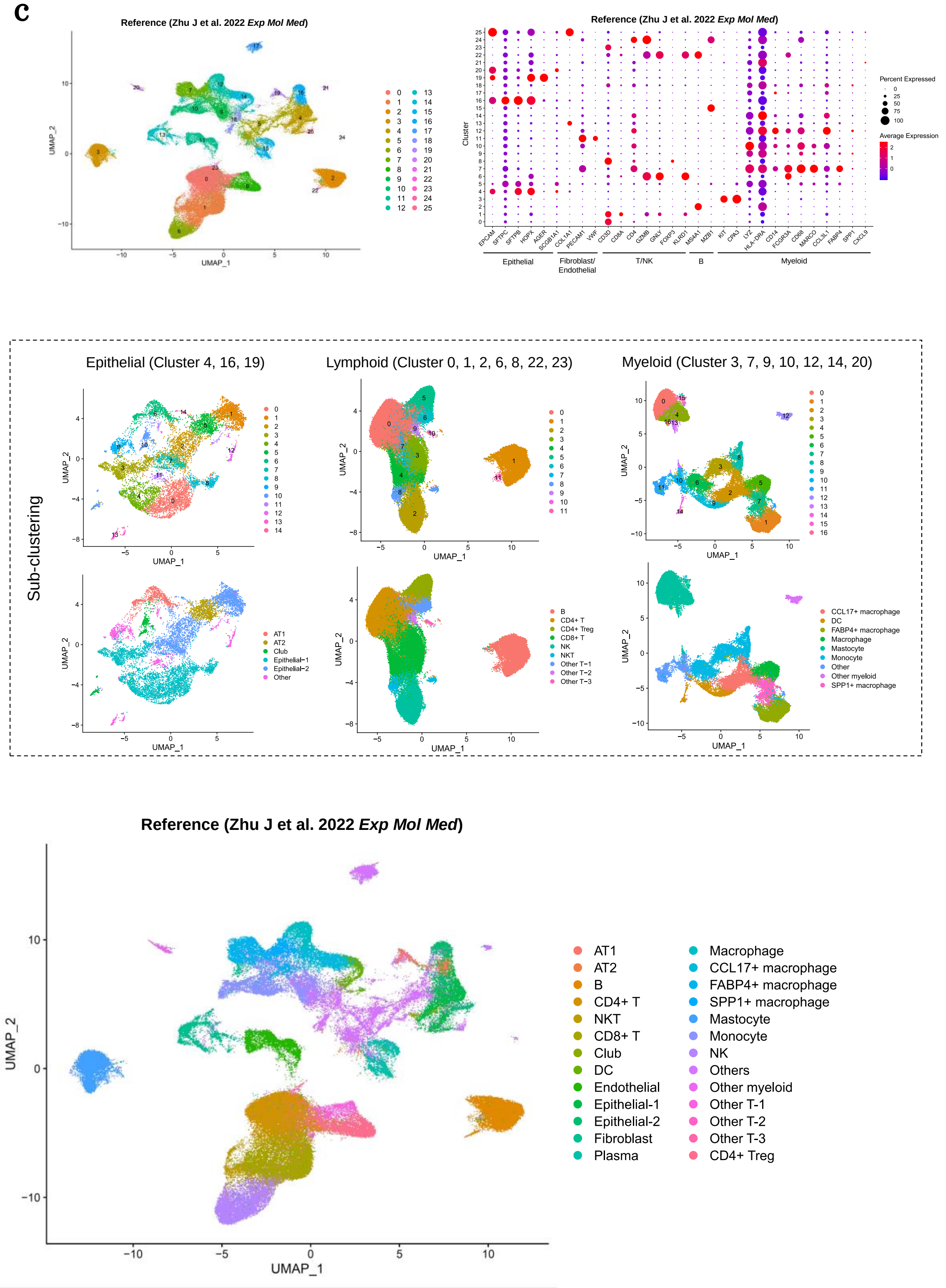

Reference (Zhu J et al. 2022 Exp Mol Med)
Reference (Zhu J et al. 2022 Exp Mol Med)
Percent Expressed
Average Expression
Cluster
Epithelial
Fibroblast/
Endothelial
T/NK
B
Myeloid
Sub-clustering
Epithelial (Cluster 4, 16, 19)
Lymphoid (Cluster 0, 1, 2, 6, 8, 22, 23)
Myeloid (Cluster 3, 7, 9, 10, 12, 14, 20)
AT1
AT2
Club
Epithelial−1
Epithelial−2
Other
B
CD4+ T
CD4+ Treg
CD8+ T
NK
NKT
Other T−1
Other T−2
Other T−3
CCL17+ macrophage
DC
FABP4+ macrophage
Macrophage
Mastocyte
Monocyte
Other
Other myeloid
SPP1+ macrophage
Reference (Zhu J et al. 2022 Exp Mol Med)
UMAP_1
UMAP_2
AT1
AT2
B
CD4+ T
NKT
CD8+ T
Club
DC
Endothelial
Epithelial-1
Epithelial-2
Fibroblast
Plasma
Macrophage
CCL17+ macrophage
FABP4+ macrophage
SPP1+ macrophage
Mastocyte
Monocyte
NK
Others
Other myeloid
Other T-1
Other T-2
Other T-3
CD4+ Treg

**d**

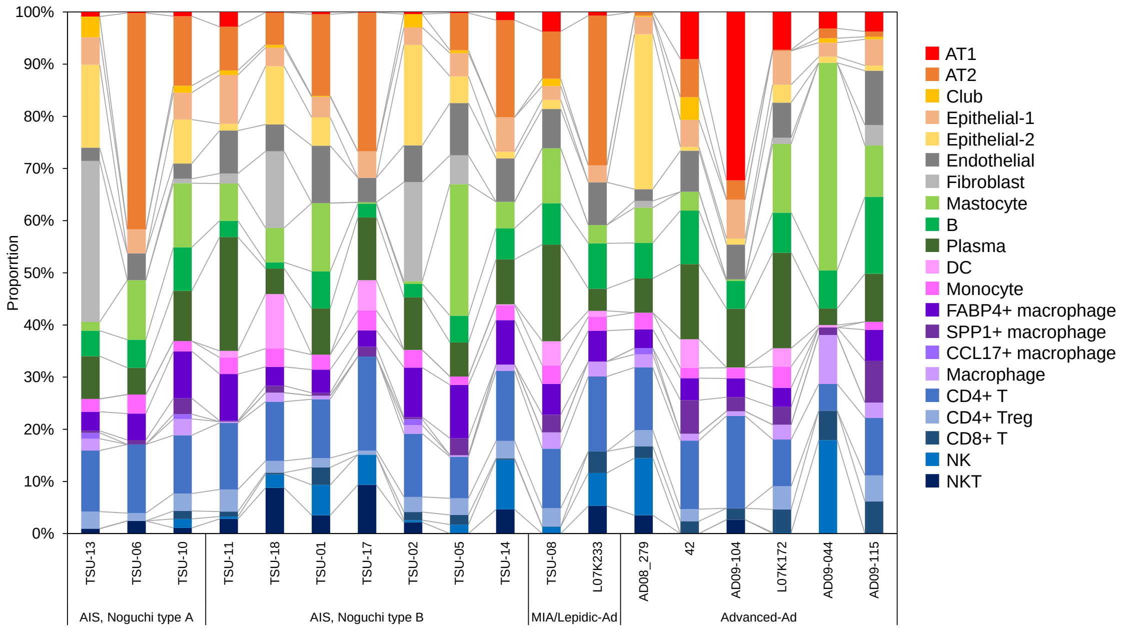

100%
90%
80%
70%
60%
50%
40%
30%
20%
10%
0%
Proportion
TSU-13
TSU-06
TSU-10
TSU-11
TSU-18
TSU-01
TSU-17
TSU-02
TSU-05
TSU-14
TSU-08
L07K233
AD08_279
42
AD09-104
L07K172
AD09-044
AD09-115
AIS, Noguchi type A
AIS, Noguchi type B
MIA/Lepidic-Ad
Advanced-Ad
AT1
AT2
Club
Epithelial-1
Epithelial-2
Endothelial
Fibroblast
Mastocyte
B
Plasma
DC
Monocyte
FABP4+ macrophage
SPP1+ macrophage
CCL17+ macrophage
Macrophage
CD4+ T
CD4+ Treg
CD8+ T
NK
NKT


**e**

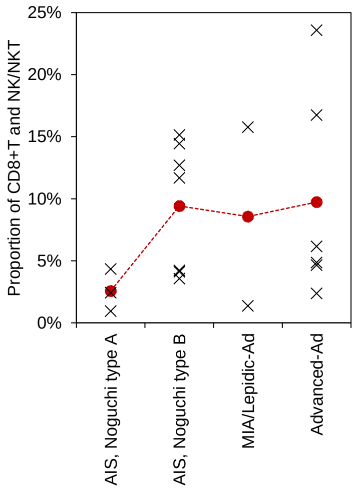

25%
20%
15%
10%
5%
0%
Proportion of CD8+T and NK/NKT
AIS, Noguchi type A
AIS, Noguchi type B
MIA/Lepidic-Ad
Advanced-Ad

f

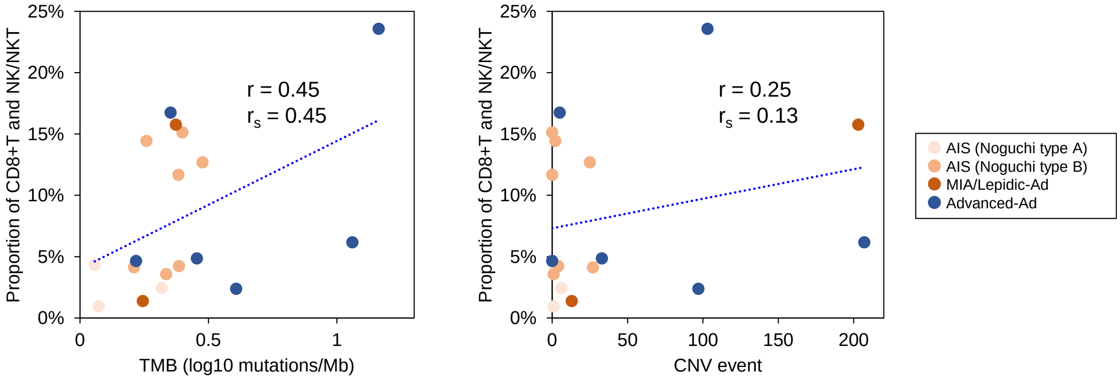


g

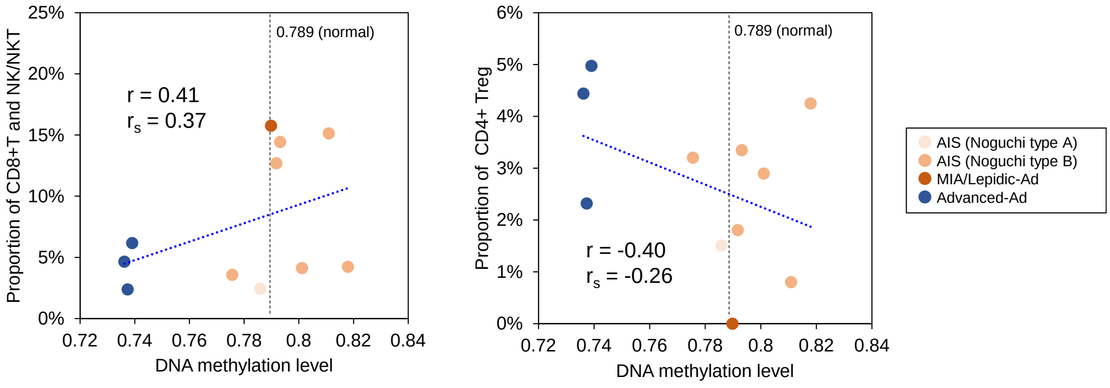


**Supplementary Figure S7 RNA-seq analysis**

(**a**) PCA plots using RNA-seq data from Early-Ad and Advanced-Ad cases. (**b**) PCA plot using deconvolution analysis of the datasets of Early-Ad cases. (**c**) Reference lung cancer scRNA-seq dataset for deconvolution analysis, constructed from publicly available[3] single-cell RNA-seq (scRNA-seq) data using Seurat. Top, UMAP plot of clusters and dot plot of marker gene expressions in each cluster. Center, the UMAP plots of sub-clustering analysis for epithelial, lymphoid, and myeloid cell clusters. Bottom, final reference constructed with annotation of 26 cell types. AT1: alveolar epithelial, type 1; AT2: alveolar epithelial, type 2; DC: dendritic cell; Treg: regulatory T. (**d**) Deconvolution analysis by CIBERSORTx for bulk RNA-seq datasets using reference scRNA-seq data. The estimated cell type fractions are shown for each case. (**e**) Distribution of the estimated fraction of cytotoxic immune cells including CD8+ T, NK, and NKT cells for each stage. (**f**) Association between the estimated fraction of cytotoxic immune cells and the tumor mutation burden (TMB) (left) or the number of mutations and CNV events

(right). Each dot represents a single case. Pearson and Spearman correlation coefficients are shown in the inset. The color key of the stage is shown in the margin. (**g**) Association between global DNA methylation rates (50 kb window; median) and the estimated fraction of cytotoxic immune cells (left) or regulatory T cells (right). The median DNA methylation rate of normal samples is shown in the plot as a control. Source data are provided as a Source Data file.

Note:

In **Supplementary Figures S7a** and **S7b**, we first observed the PCA plots to understand the transcriptome landscape of lung adenocarcinoma. Some AIS Noguchi type B cases showed a distinctive landscape, which indicated that transcriptome signatures of Noguchi type B tumors might differ from those of Noguchi type A tumors due to the beginning of alveolar collapse and interactions with microenvironment.

In **Supplementary Figures S7c–S7g**, we performed deconvolution analysis of bulk RNA-seq data to characterize immune features. As a result, various types of immune cells were infiltrated; in some cases at the Noguchi type B stage, the proportion of cytotoxic immune cells including CD8+ T cells, NK and NKT cells increased. Next, to compare our data with recent publications, as indicated by the reviewer, we evaluated the association between the immune infiltration levels and several omics features, such as TMB, CNV burden and DNA methylation levels. We found that TMB showed a slight positive correlation with the infiltration levels of cytotoxic cells while the number of CNV events was not significantly correlated. We also found that genome-wide DNA hypomethylation was associated with increased CD4+ Treg and decreased cytotoxic immune cells. This result indicates that global hypomethylation is associated with immune suppression, which is consistent with previous results[4].

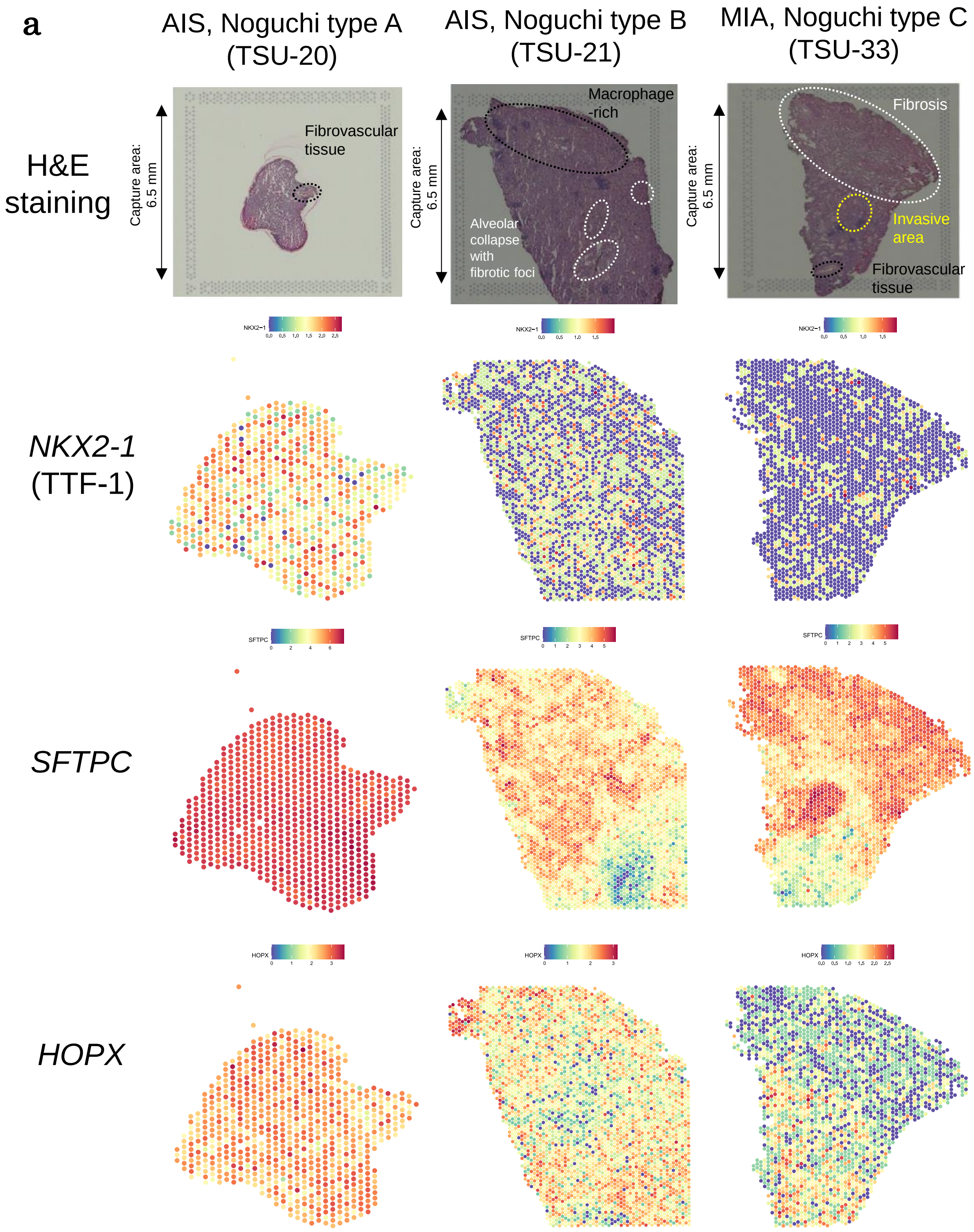

a
AIS, Noguchi type A (TSU-20)
AIS, Noguchi type B (TSU-21)
MIA, Noguchi type C (TSU-33)
H&E staining
Capture area: 6.5 mm
Fibrovascular tissue
Macrophage -rich
Alveolar collapse with fibrotic foci
Fibrosis
Invasive area
Fibrovascular tissue
NKX2-1 (TTF-1)
SFTPC
HOPX

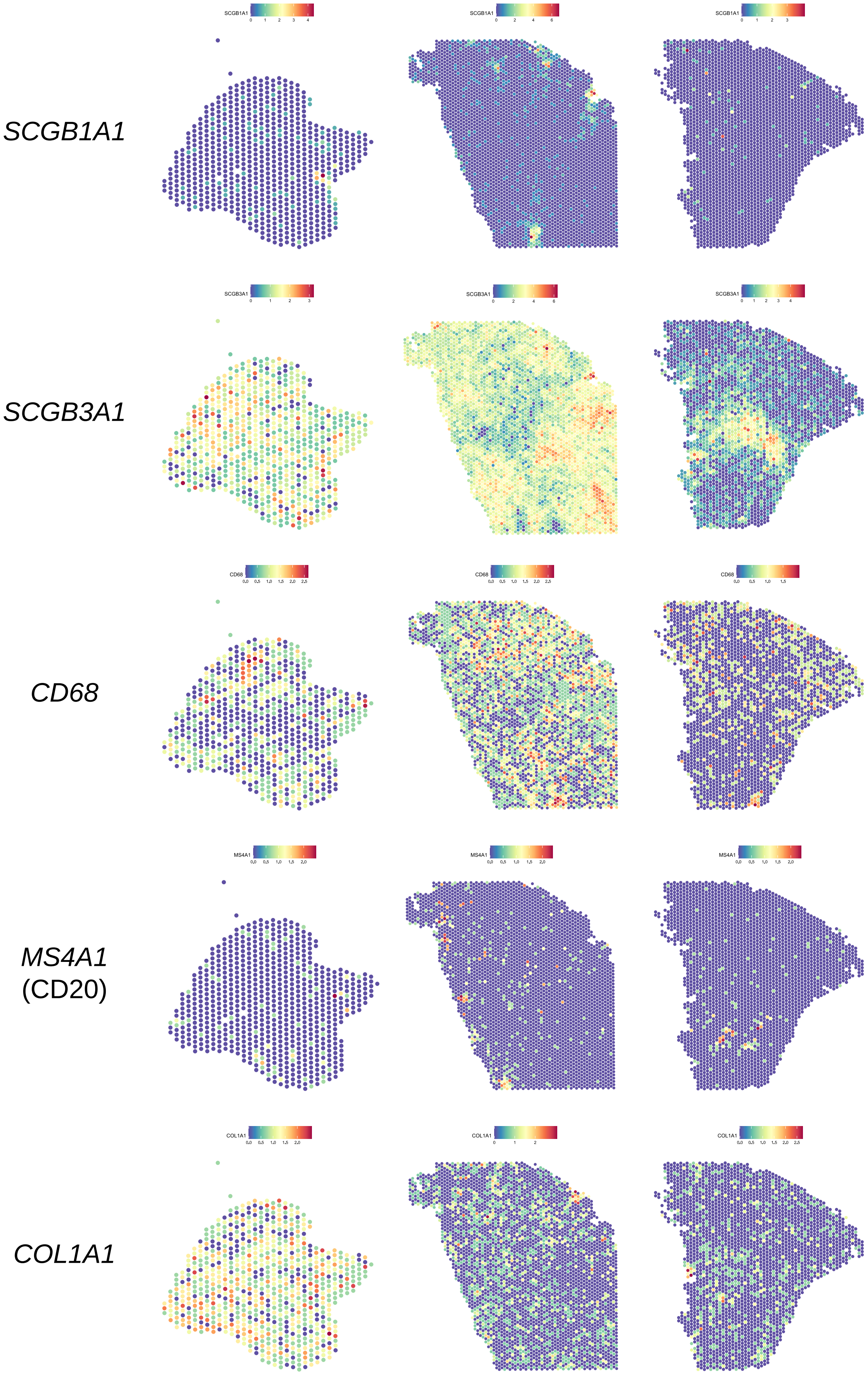
SCGB1A1
SCGB3A1
CD68
MS4A1
(CD20)
COL1A1

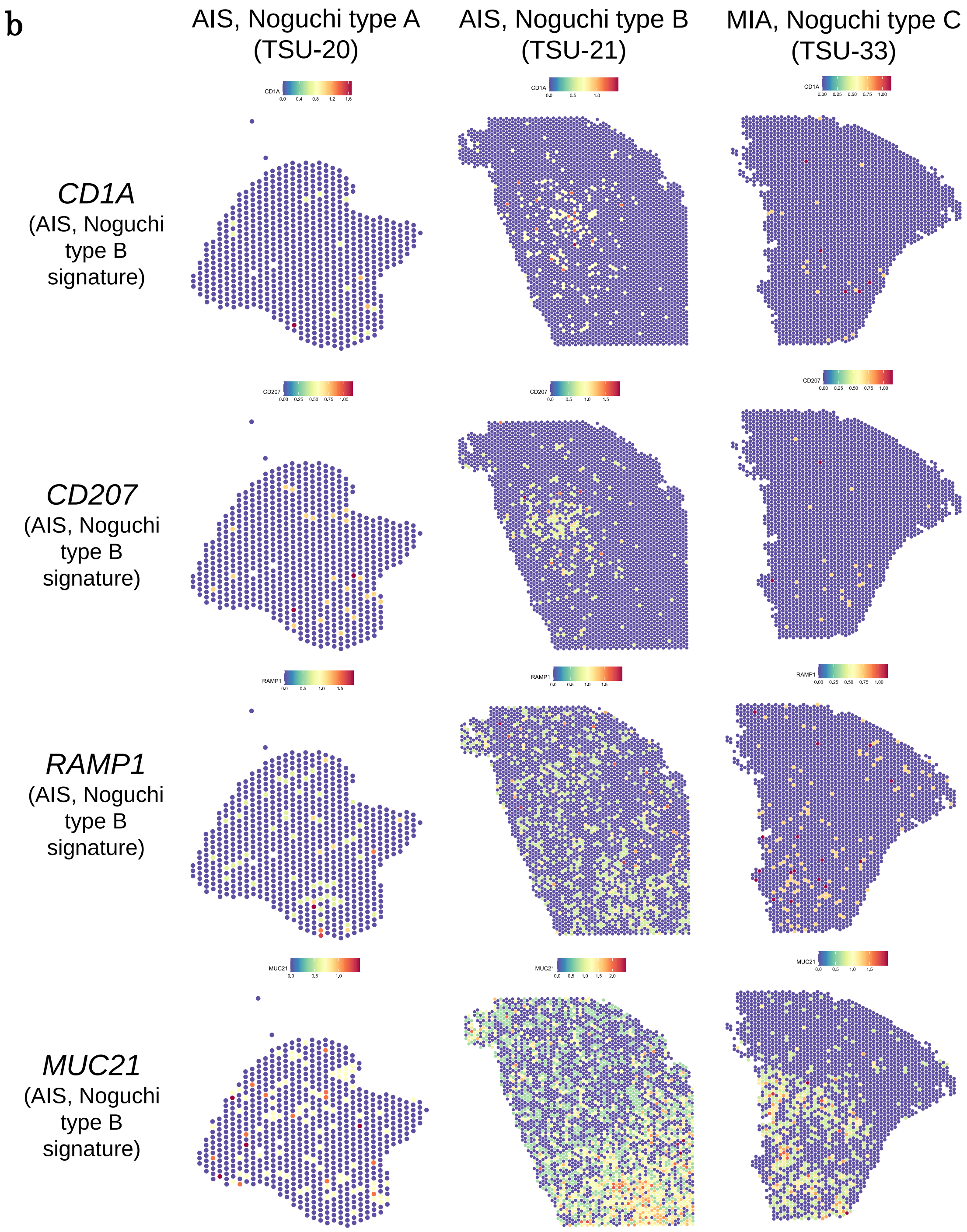
b
AIS, Noguchi type A (TSU-20)
AIS, Noguchi type B (TSU-21)
MIA, Noguchi type C (TSU-33)
*CD1A* (AIS, Noguchi type B signature)
*CD207* (AIS, Noguchi type B signature)
*RAMP1* (AIS, Noguchi type B signature)
*MUC21* (AIS, Noguchi type B signature)

**c**

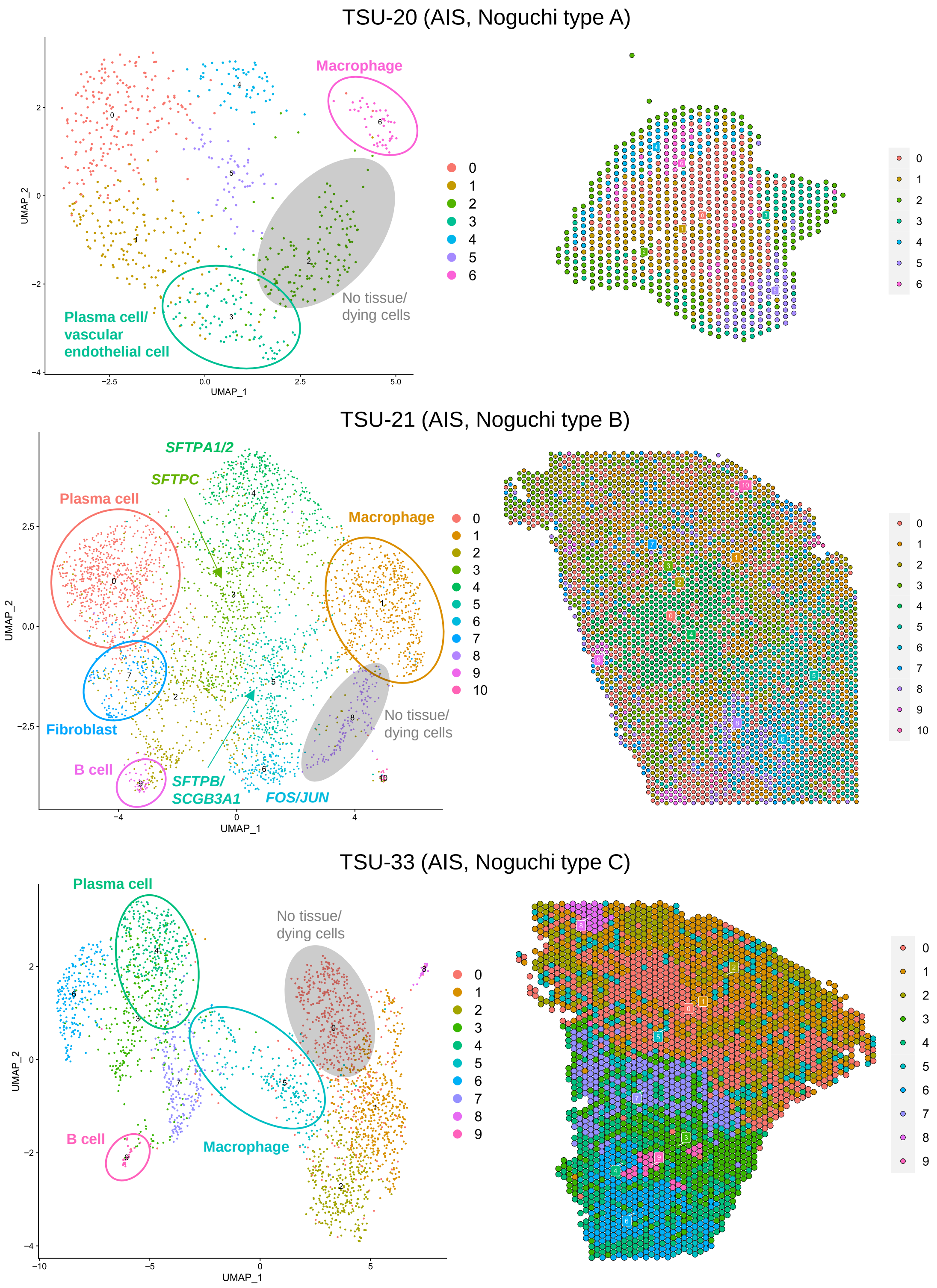
TSU-20 (AIS, Noguchi type A)
Macrophage
No tissue/
dying cells
Plasma cell/
vascular
endothelial cell
UMAP_1
UMAP_2
TSU-21 (AIS, Noguchi type B)
SFTPA1/2
SFTPC
Plasma cell
Macrophage
Fibroblast
No tissue/
dying cells
B cell
SFTPB/
SCGB3A1
FOS/JUN
UMAP_1
UMAP_2
TSU-33 (AIS, Noguchi type C)
Plasma cell
No tissue/
dying cells
B cell
Macrophage
UMAP_1
UMAP_2

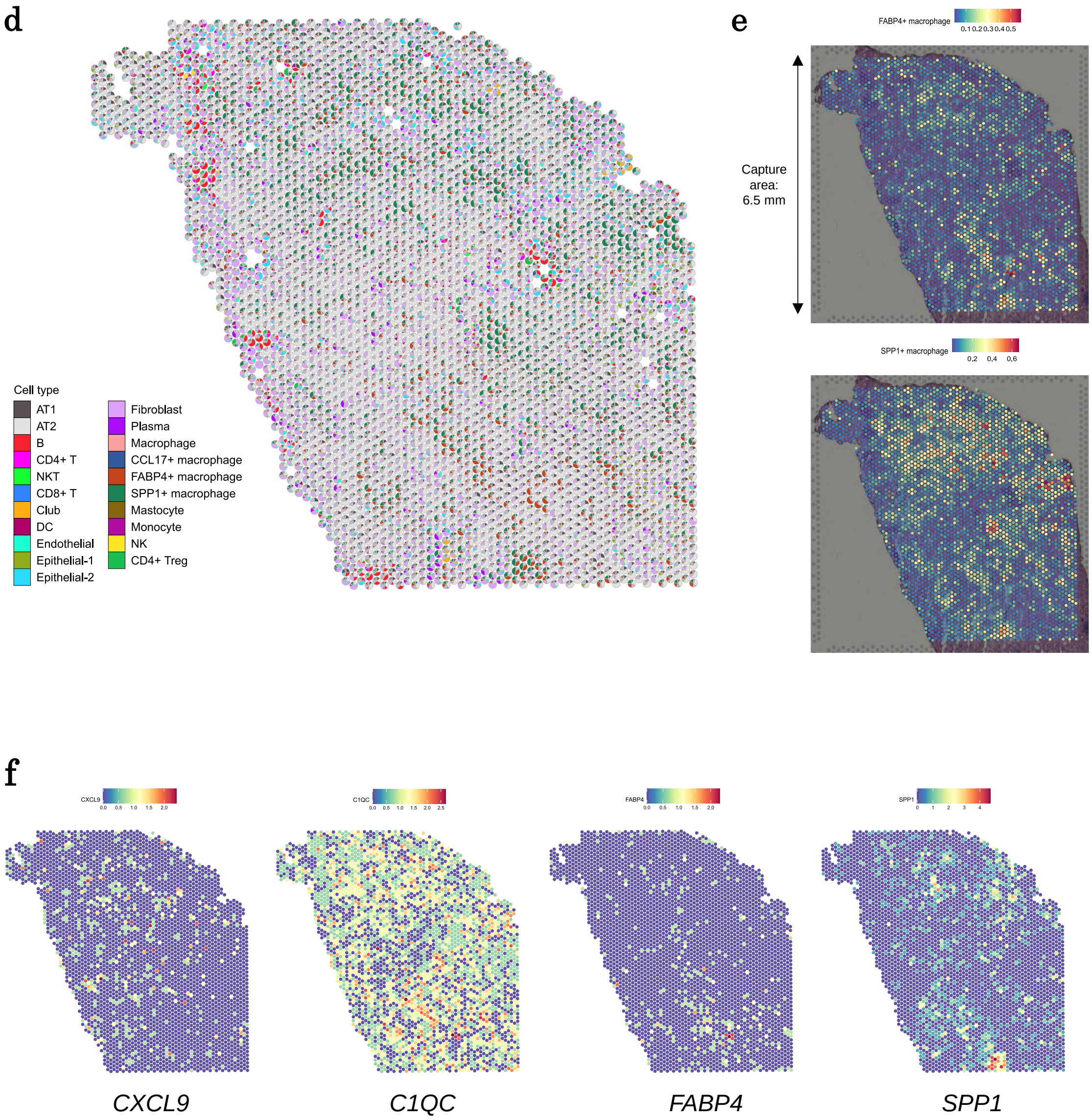


**g**

Weight (color scale: 0, 0.5, 2.4)

| | Cluster 0 | Cluster 1 | Cluster 2 | Cluster 3 | Cluster 4 | Cluster 5 | Cluster 6 | Cluster 7 | Cluster 8 | Cluster 9 | Cluster 10 |
|---|---|---|---|---|---|---|---|---|---|---|---|
| Cluster 0 | 0.39 | 1.16 | 0.54 | 0.74 | 0.39 | 0.47 | 0.12 | 0.46 | 0.50 | 0.05 | 0.00 |
| Cluster 1 | 0.56 | 1.95 | 0.66 | 0.90 | 0.42 | 0.60 | 0.15 | 0.80 | 0.72 | 0.00 | 0.00 |
| Cluster 2 | 0.17 | 1.18 | 0.42 | 0.78 | 0.30 | 0.42 | 0.07 | 0.29 | 0.41 | 0.04 | 0.00 |
| Cluster 3 | 0.28 | 1.36 | 0.41 | 0.61 | 0.40 | 0.35 | 0.00 | 0.45 | 0.42 | 0.07 | 0.00 |
| Cluster 4 | 0.19 | 0.59 | 0.18 | 0.29 | 0.98 | 0.10 | 0.00 | 0.28 | 0.24 | 0.00 | 0.00 |
| Cluster 5 | 0.02 | 0.68 | 0.07 | 0.09 | 0.10 | 0.36 | 0.14 | 0.12 | 0.22 | 0.00 | 0.00 |
| Cluster 6 | 0.03 | 0.15 | 0.03 | 0.00 | 0.00 | 0.15 | 0.93 | 0.00 | 0.21 | 0.00 | 0.00 |
| Cluster 7 | 1.45 | 2.17 | 1.46 | 1.68 | 0.89 | 0.84 | 0.00 | 2.38 | 1.29 | 0.00 | 0.00 |
| Cluster 8 | 0.11 | 0.81 | 0.31 | 0.34 | 0.21 | 0.29 | 0.30 | 0.33 | 1.08 | 0.00 | 0.00 |
| Cluster 9 | 0.01 | 0.00 | 0.01 | 0.02 | 0.00 | 0.00 | 0.00 | 0.00 | 0.00 | 0.26 | 0.00 |
| Cluster 10 | 0.00 | 0.00 | 0.00 | 0.00 | 0.00 | 0.00 | 0.00 | 0.00 | 0.00 | 0.00 | 0.14 |

Count (color scale: 0, 50, 200)

| | Cluster 0 | Cluster 1 | Cluster 2 | Cluster 3 | Cluster 4 | Cluster 5 | Cluster 6 | Cluster 7 | Cluster 8 | Cluster 9 | Cluster 10 |
|---|---|---|---|---|---|---|---|---|---|---|---|
| Cluster 0 | 27 | 79 | 24 | 40 | 62 | 34 | 64 | 69 | 63 | 18 | 0 |
| Cluster 1 | 34 | 117 | 24 | 81 | 101 | 44 | 96 | 120 | 102 | 0 | 0 |
| Cluster 2 | 10 | 55 | 12 | 24 | 39 | 14 | 38 | 42 | 44 | 11 | 0 |
| Cluster 3 | 56 | 141 | 46 | 111 | 145 | 82 | 0 | 146 | 146 | 45 | 0 |
| Cluster 4 | 79 | 150 | 58 | 123 | 150 | 96 | 0 | 159 | 160 | 0 | 0 |
| Cluster 5 | 10 | 65 | 10 | 40 | 71 | 19 | 73 | 65 | 65 | 0 | 0 |
| Cluster 6 | 72 | 148 | 44 | 0 | 0 | 94 | 165 | 0 | 140 | 0 | 0 |
| Cluster 7 | 112 | 185 | 78 | 149 | 182 | 105 | 0 | 190 | 181 | 0 | 0 |
| Cluster 8 | 34 | 119 | 32 | 93 | 108 | 57 | 119 | 131 | 120 | 0 | 0 |
| Cluster 9 | 8 | 0 | 6 | 15 | 0 | 0 | 0 | 0 | 0 | 20 | 0 |
| Cluster 10 | 0 | 0 | 0 | 0 | 0 | 0 | 0 | 0 | 0 | 0 | 37 |

Cluster 0: Plasma
Cluster 1: Macrophage
Cluster 2: Others
Cluster 3: *SFTPC*+ tumor
Cluster 4: *SFTPA1/2*+ tumor
Cluster 5: *SFTPB*+ tumor
Cluster 6: *FOS/JUN* tumor
Cluster 7: Fibroblast
Cluster 8: Others
Cluster 9: B
Cluster 10: Others

**h**

SPP1 signaling pathway network

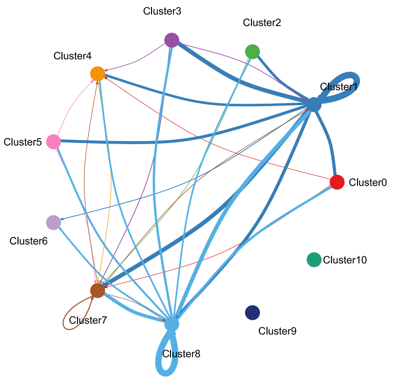


| Pathway name | Source | Target | Ligand - receptor | Probability |
|---|---|---|---|---|
| SPP1 | Cluster1 | Cluster7 | SPP1 - CD44 | 0.012 |
| SPP1 | Cluster1 | Cluster7 | SPP1 - (ITGA5+ITGB1) | 0.0067 |
| SPP1 | Cluster1 | Cluster7 | SPP1 - (ITGAV+ITGB1) | 0.0064 |
| SPP1 | Cluster1 | Cluster7 | SPP1 - (ITGAV+ITGB5) | 0.0048 |
| SPP1 | Cluster1 | Cluster7 | SPP1 - (ITGAV+ITGB6) | 0.0045 |
| SPP1 | Cluster1 | Cluster7 | SPP1 - (ITGA9+ITGB1) | 0.0042 |
| SPP1 | Cluster1 | Cluster7 | SPP1 - (ITGA4+ITGB1) | 0.0032 |
| SPP1 | Cluster1 | Cluster7 | SPP1 - (ITGA8+ITGB1) | 0.0012 |

**Supplementary Figure S8 Spatial transcriptome analysis of early cases**

(**a**) H&E images and representative cell-type marker expression patterns. The Visium capture area surrounded by the fiducial frame is 6.5 mm × 6.5 mm. Expression levels of Visium data are shown by Seurat using SCTransform normalized data. (**b**) Spatial expression patterns of representative AIS Noguchi type B signature genes. (**c**) Clustering and UMAP visualization of Visium data for the three cases. Cell type annotation is shown in the margin of the UMAP plot. (**d**) Deconvolution of Visium data from case TSU-21 using the reference scRNA-seq data (**Supplementary Fig. S7c**)[3] by spacexr RCTD. Each pie chart represents the estimated cell type fraction in each spot. (**e**) Fraction of FABP+ macrophages and SPP+ macrophages estimated by deconvolution analysis using the reference scRNA-seq data. The Visium capture area surrounded by the fiducial frame is 6.5 mm × 6.5 mm. (**f**) Spatial expression patterns of representative macrophage markers in the Visium data of case TSU-21. (**g**) Ligand and receptor interaction analysis by CellChat using Visium data of case TSU-21. The weight and interaction count among clusters are shown as well. (**h**) “SPP1 signaling pathway network” as an example of ligand-receptor interactions. Left, circle plot; right, the interaction details between cluster 1 (macrophage) and cluster 7 (fibroblast).

a

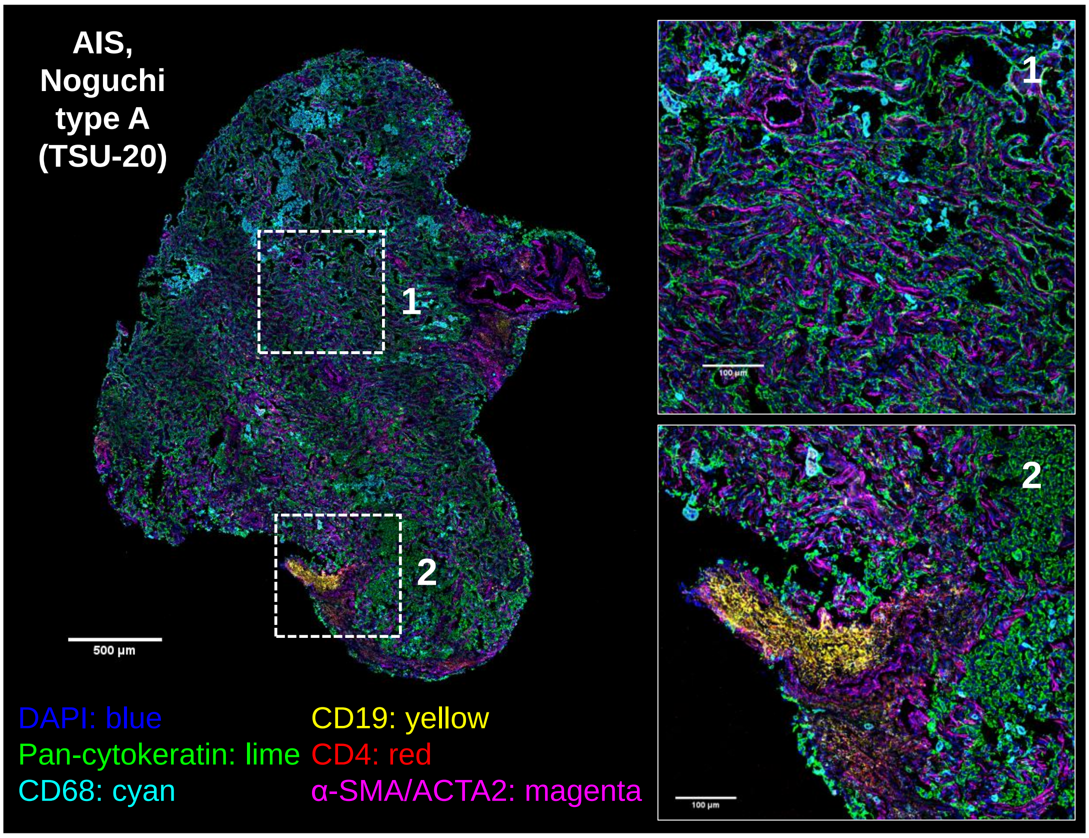
AIS,
Noguchi
type A
(TSU-20)
1
2
500 µm
100 µm
DAPI: blue
Pan-cytokeratin: lime
CD68: cyan
CD19: yellow
CD4: red
α-SMA/ACTA2: magenta

b

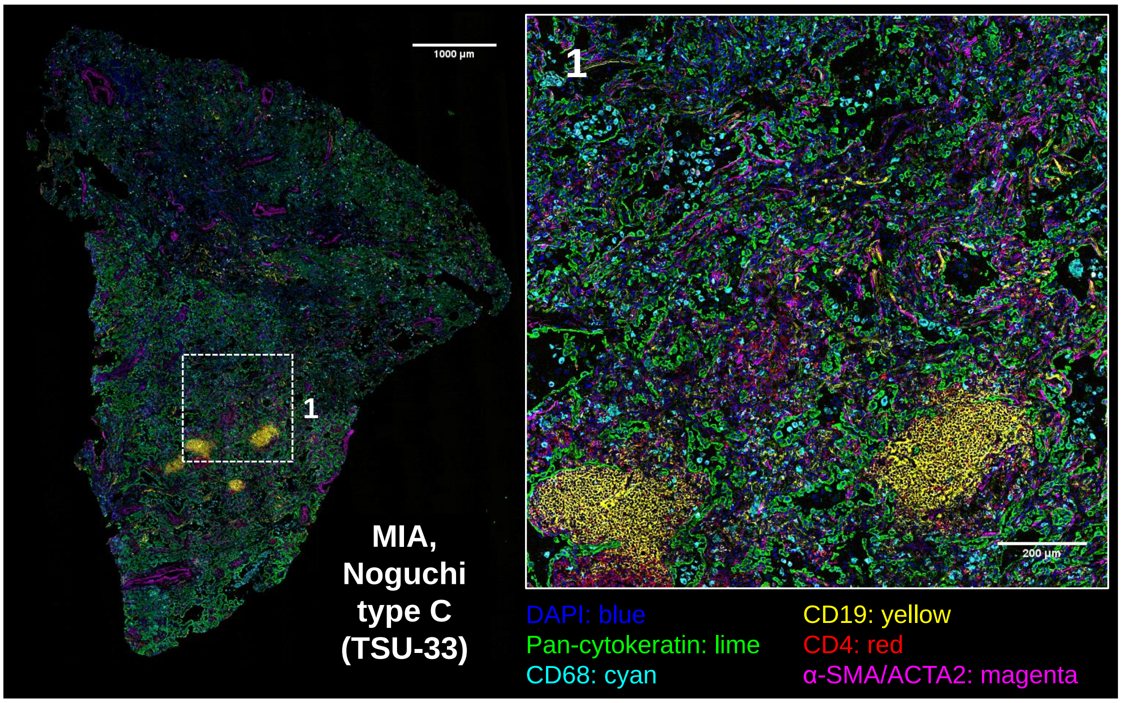
1000 µm
1
200 µm
MIA,
Noguchi
type C
(TSU-33)
DAPI: blue
Pan-cytokeratin: lime
CD68: cyan
CD19: yellow
CD4: red
α-SMA/ACTA2: magenta

**Supplementary Figure S9 Multiplexed fluorescence immunostaining analysis for early cases**

Representative cell-type marker expression patterns visualized with multiplexed fluorescence immunostaining (PhenoCycler) of serial sections of spatial transcriptome data. The entire region and some regions-of-interests (ROIs) are shown. Scale bars are shown in the each image. (**a**) For case TSU-20 (AIS, Noguchi type A), the following two ROIs are shown: 1) a region of tumors with pure-lepidic growth and alveolar macrophages, and 2) a region with lymphocyte infiltration. (**b**) For case TSU-33 (MIA, Noguchi type C), one ROI is shown as a region nearby lymphocyte infiltration and invasion. Each antibody was used as a corresponding cell-type marker as follows: Pan-cytokeratin, epithelial cells; CD19, B cells; CD4, T cells; CD68, macrophages; and α-SMA/ACTA2, myofibroblasts.

a

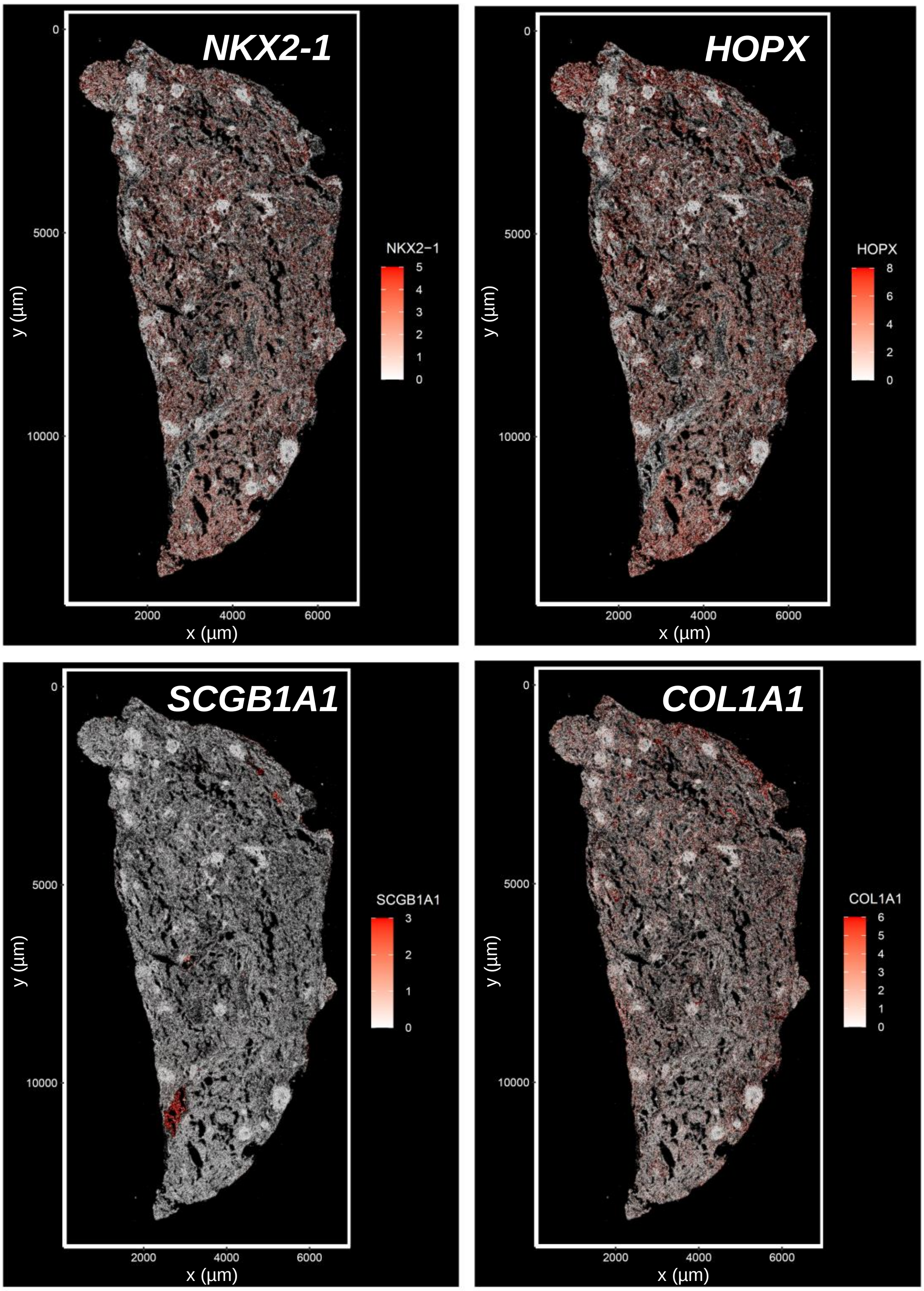

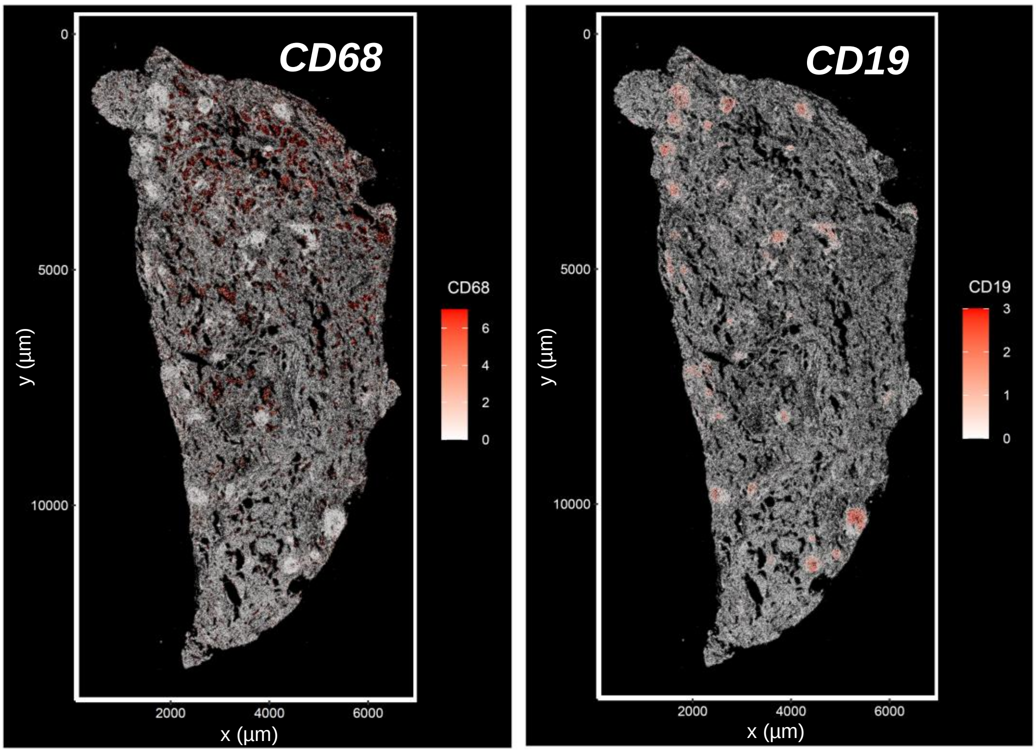
CD68
CD19
CD68
6
4
2
0
CD19
3
2
1
0
0
5000
10000
y (µm)
2000
4000
6000
x (µm)

b

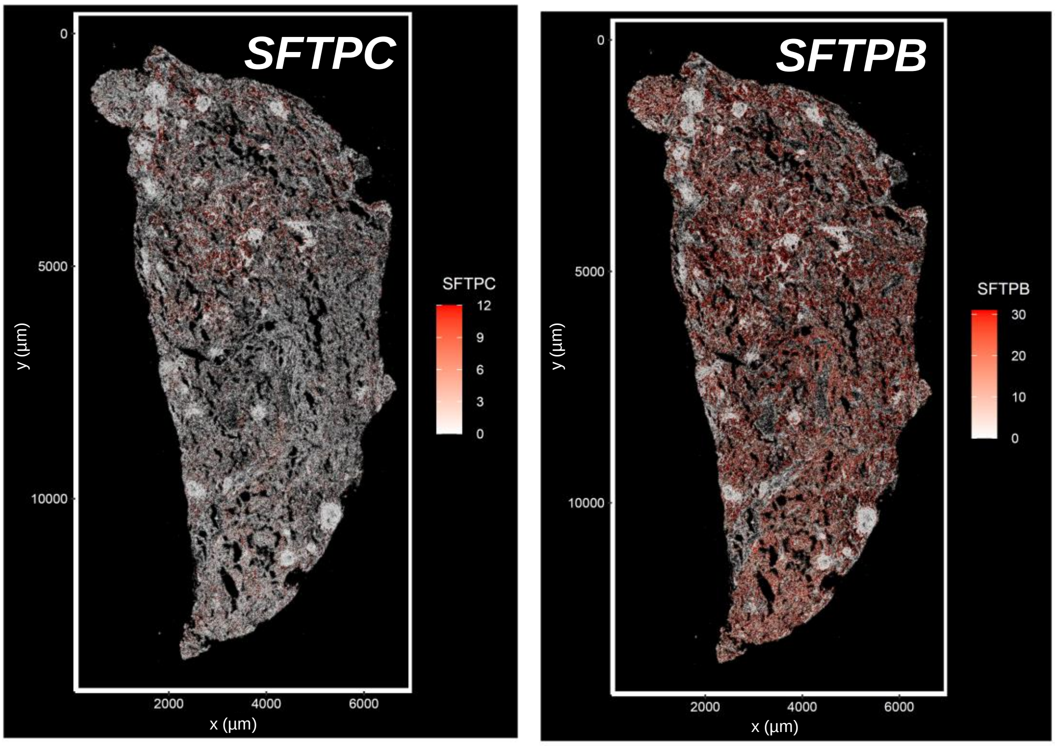
SFTPC
SFTPB
SFTPC
12
9
6
3
0
SFTPB
30
20
10
0
0
5000
10000
y (µm)
2000
4000
6000
x (µm)

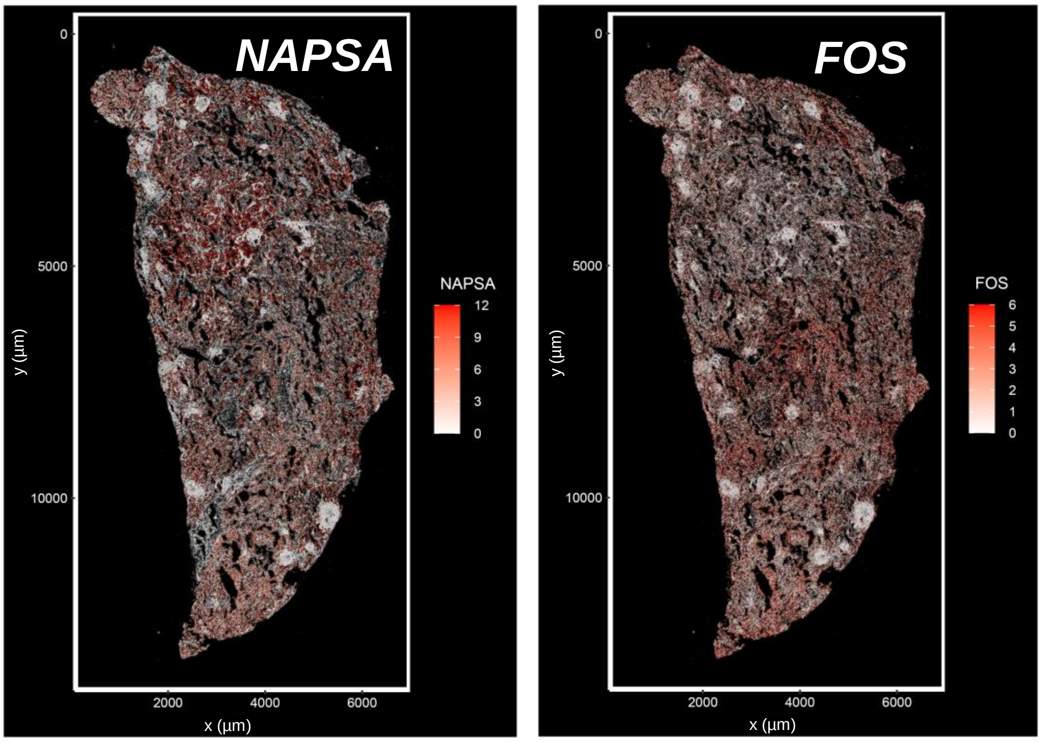
NAPSA
FOS
y (µm)
x (µm)
0
5000
10000
2000
4000
6000
NAPSA
12
9
6
3
0
FOS
6
5
4
3
2
1
0

**c**

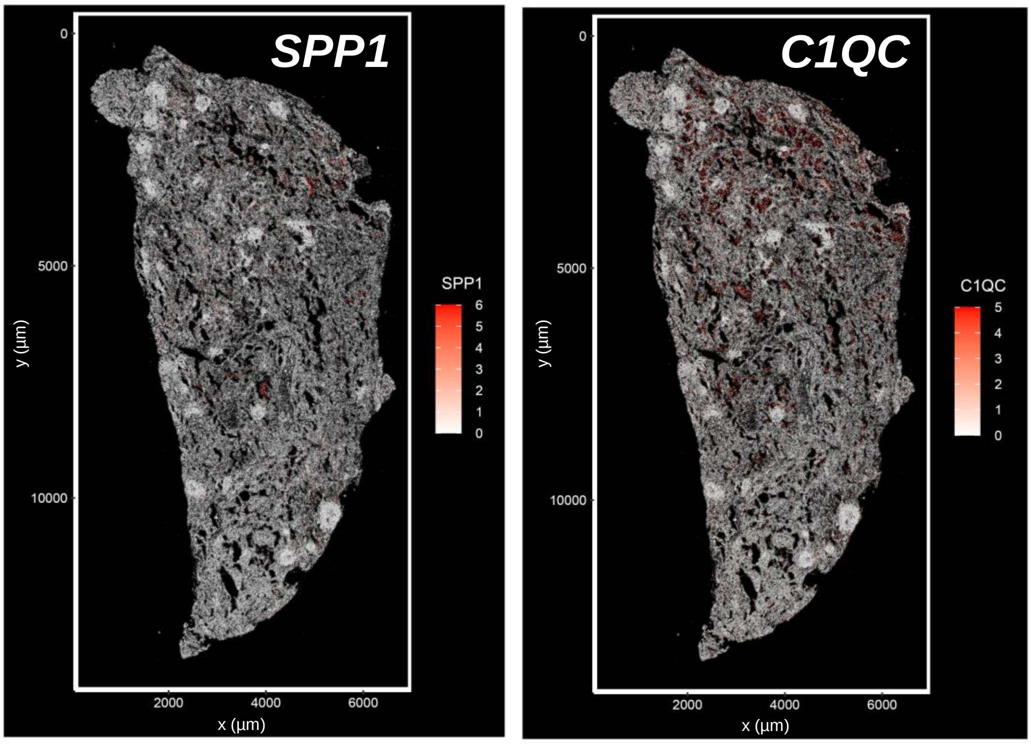
SPP1
C1QC
y (µm)
x (µm)
0
5000
10000
2000
4000
6000
SPP1
6
5
4
3
2
1
0
C1QC
5
4
3
2
1
0

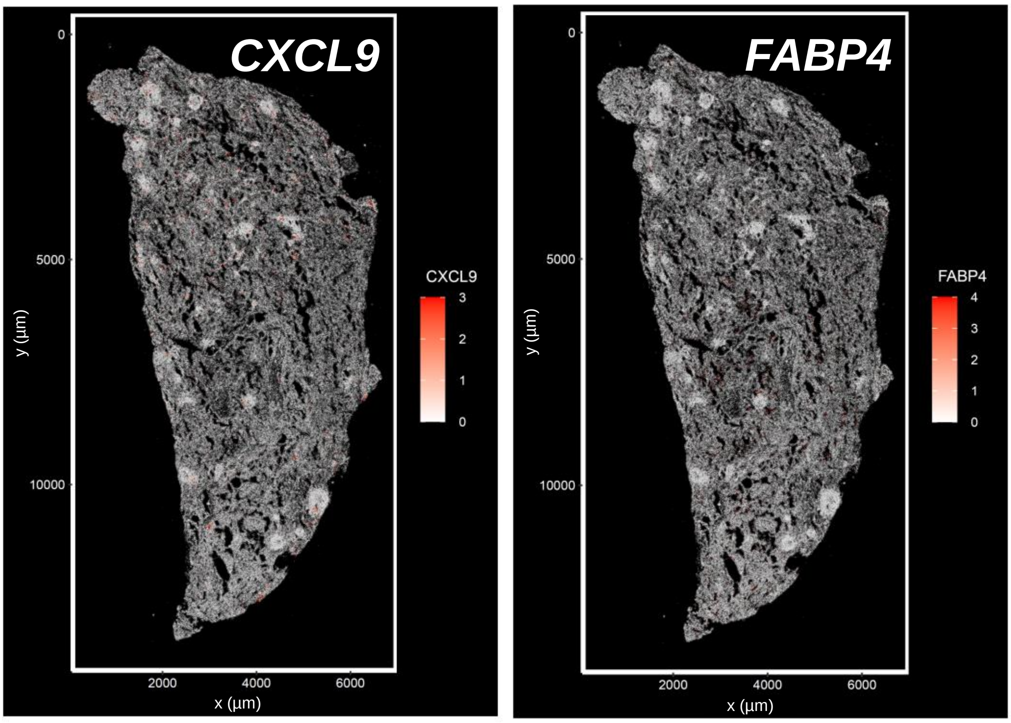
CXCL9
y (µm)
0
5000
10000
2000
4000
6000
x (µm)
CXCL9
3
2
1
0
FABP4
y (µm)
0
5000
10000
2000
4000
6000
x (µm)
FABP4
4
3
2
1
0

d

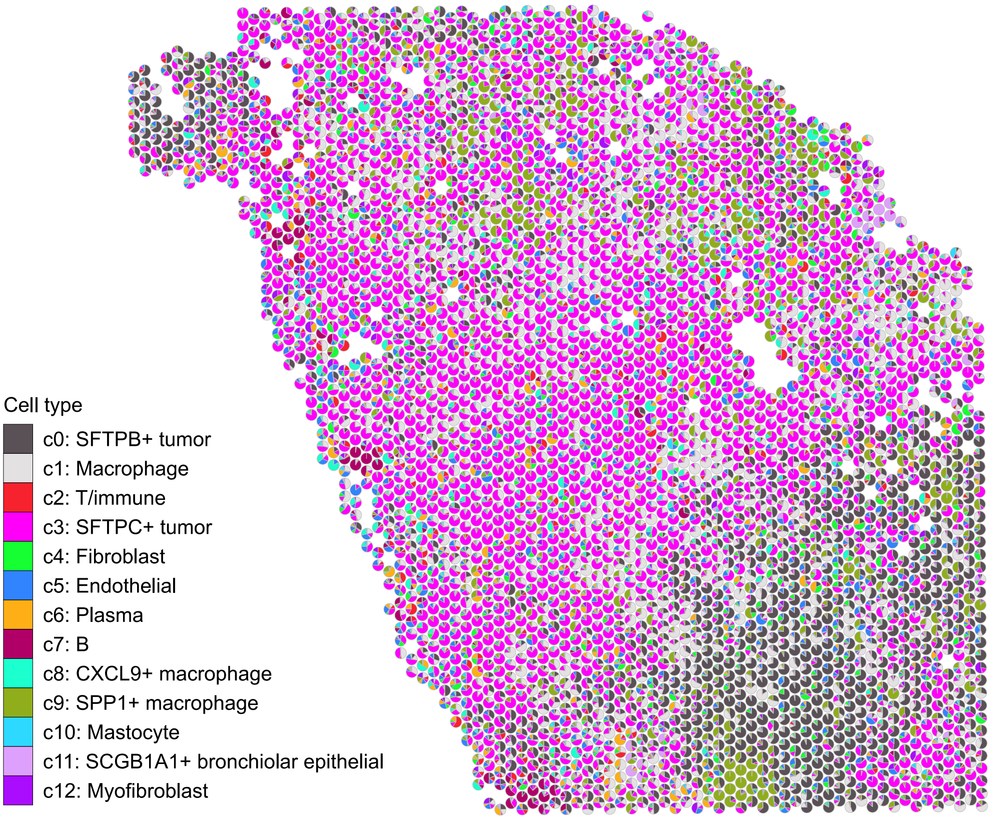
Cell type
c0: SFTPB+ tumor
c1: Macrophage
c2: T/immune
c3: SFTPC+ tumor
c4: Fibroblast
c5: Endothelial
c6: Plasma
c7: B
c8: CXCL9+ macrophage
c9: SPP1+ macrophage
c10: Mastocyte
c11: SCGB1A1+ bronchiolar epithelial
c12: Myofibroblast

e

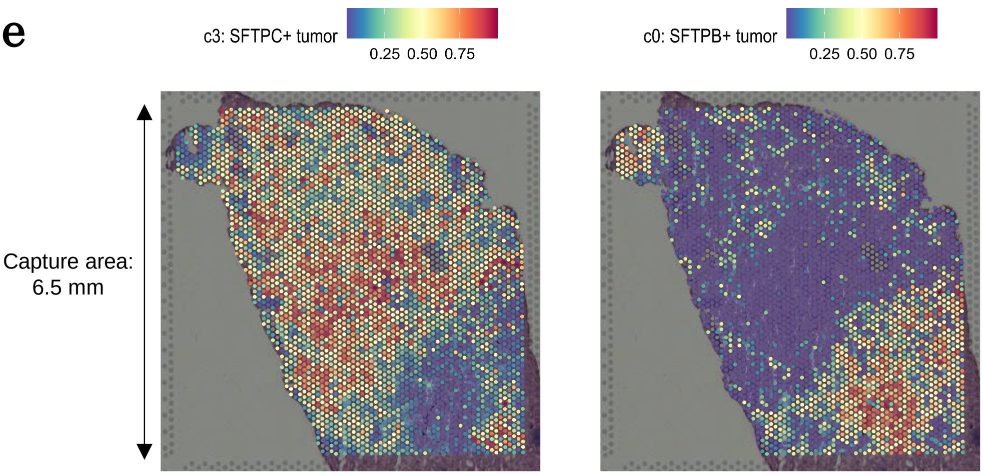


f

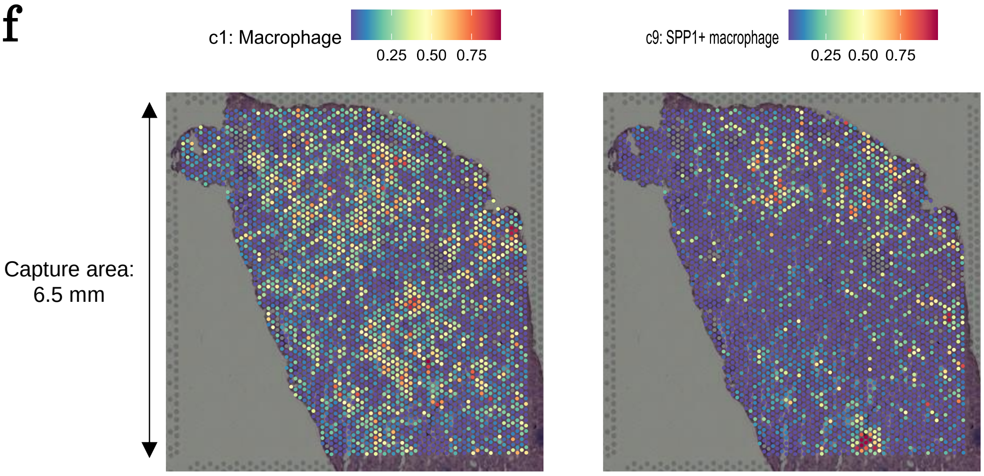


g

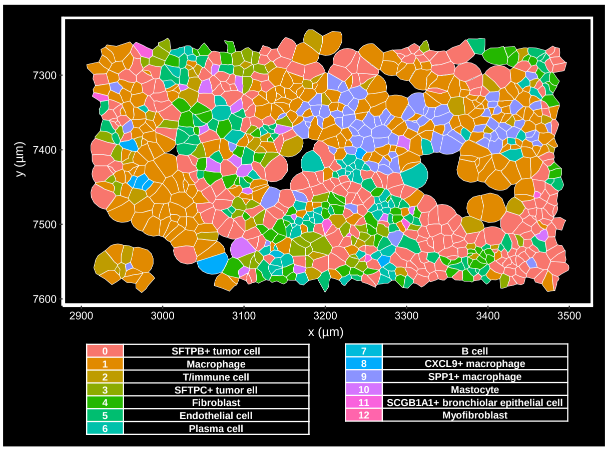


**Supplementary Figure S10 *In situ* gene expression analysis for the Noguchi type B case (case TSU-21)**

(**a**) Spatial expression patterns of representative cell type markers. The count data is

shown as expression levels. (**b**) Spatial expression patterns of genes possibly associated with tumor cell progression. (**c**) Spatial expression patterns of macrophage markers. (**d**) Deconvolution analysis of Visium using Xenium data by spacexr RCTD. Each pie chart represents the estimated cell type fraction in each spot. (**e**) Fraction of high *SFTPC* and *SFTPB* tumor cells estimated by deconvolution analysis using Xenium data. (**f**) Fraction of macrophages and SPP1+ macrophages estimated by deconvolution analysis using Xenium data. (**g**) Local patterns of clusters in the local region (the same region as **Fig. 6j**).

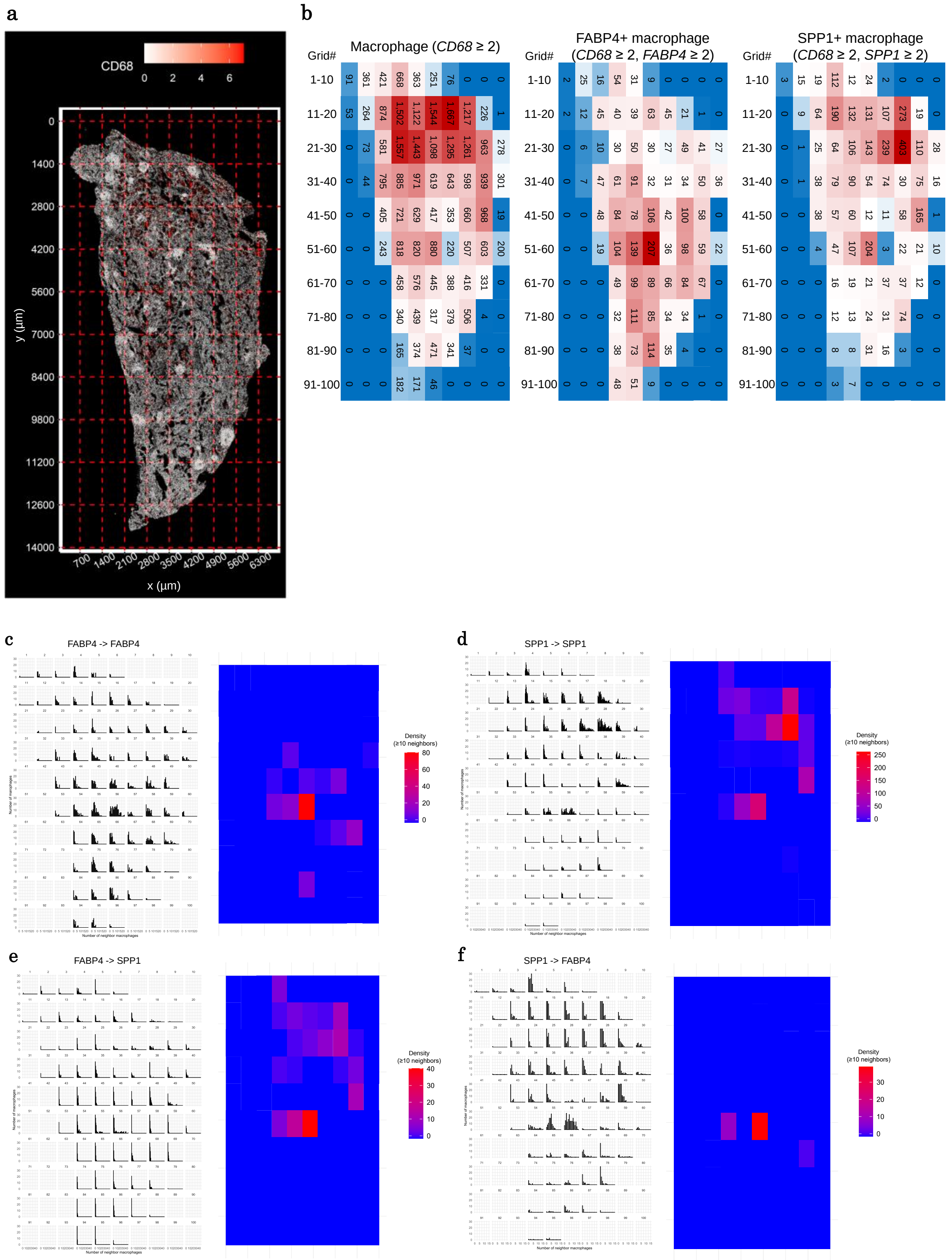


**Supplementary Figure S11 Distribution and co-localization of macrophages in the Noguchi type B case**

(**a**) The spatial RNA expression pattern of CD68 molecules of case TSU-21. For the

further analyses, the tissue region was divided into 100 parts (700 μm × 1,400 μm each) which are shown in red grid lines. (**b**) The number of macrophages in each tissue part. Total macrophages, FABP4+ macrophages, SPP1+ macrophages are shown in the left, middle and right panels, respectively. (**c–f**) Co-localization of macrophages. Neighborhood relationships from a FABP4+ macrophage to FABP4+ macrophages ("FABP4 -> FABP4") are represented in **c**. Those in "SPP1 -> SPP1", "FABP4 -> SPP1" and "SPP1 -> FABP4" are also shown in **d**, **e** and **f**, respectively. For each, distributions of the number of neighbor macrophages in each tissue part are shown in the graphs (left). The heatmaps represent the frequencies of macrophages co-localized with ≥10 macrophages (right). FABP4+ macrophages and SPP1+ macrophages were co-localized in the grid #54–56 where fibrotic foci with alveolar collapse were observed.

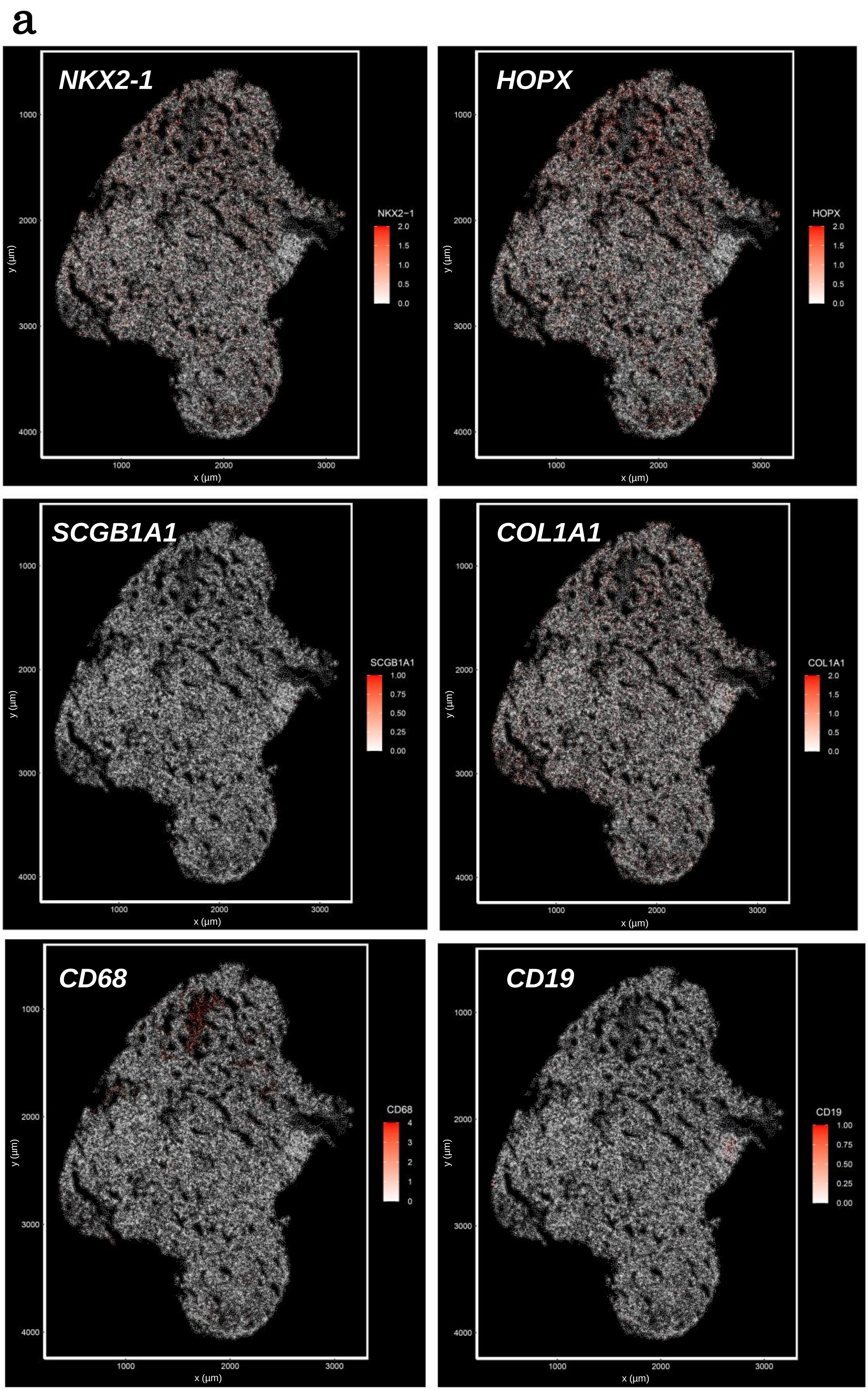
a
NKX2-1
HOPX
SCGB1A1
COL1A1
CD68
CD19
y (µm)
x (µm)

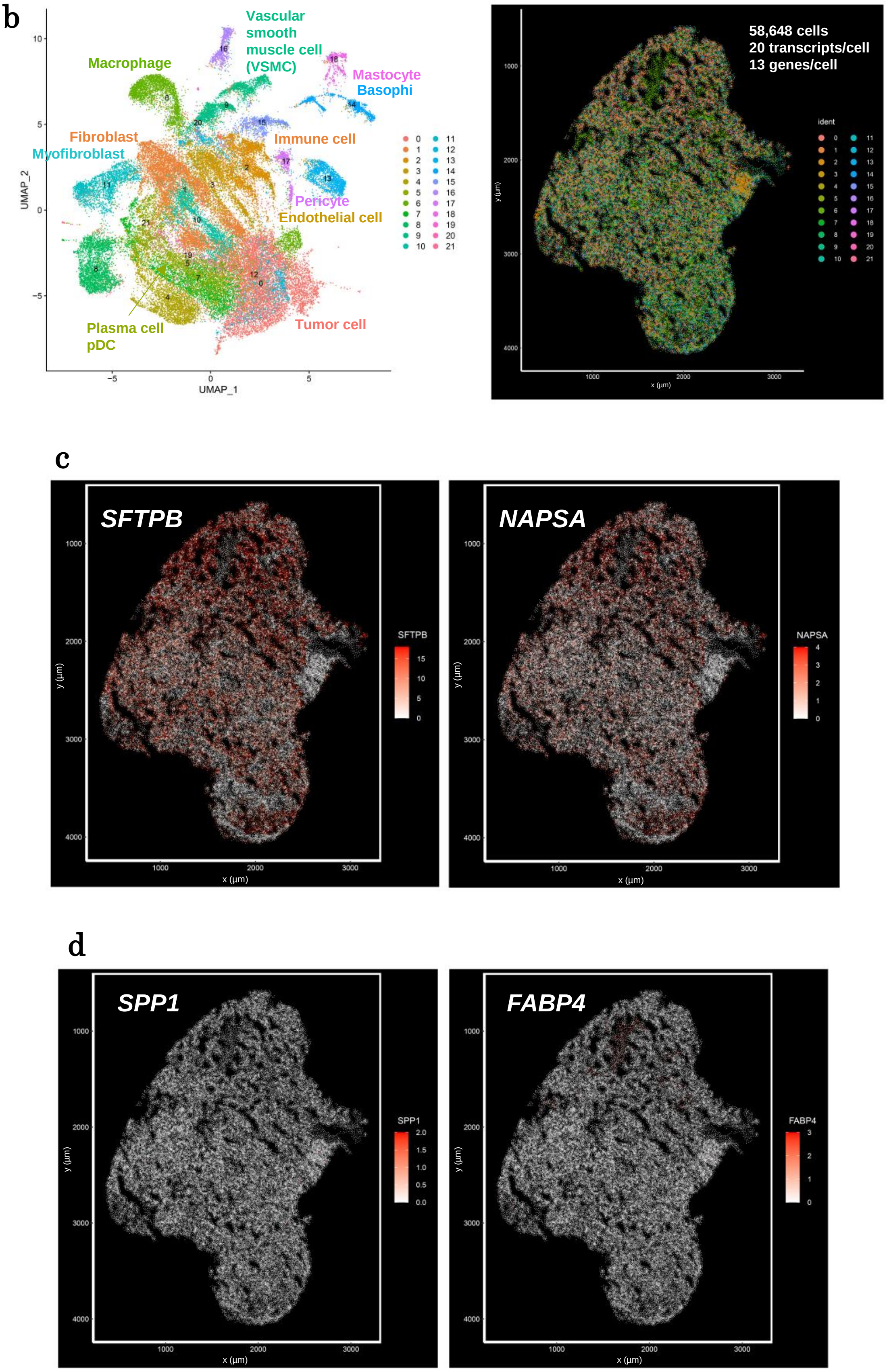
b
Vascular smooth muscle cell (VSMC)
Macrophage
Mastocyte
Basophi
Fibroblast
Myofibroblast
Immune cell
Pericyte
Endothelial cell
Plasma cell
pDC
Tumor cell
UMAP_1
UMAP_2
58,648 cells
20 transcripts/cell
13 genes/cell
ident
x (µm)
y (µm)
c
SFTPB
NAPSA
d
SPP1
FABP4

e

| Case | | TSU-20 | TSU-21 |
|---|---|---|---|
| Noguchi type | | A | B |
| Number of cells | Total (excluding empty cells) | 58,449 | 351,738 |
| | Macrophage (*CD68* ≥ 2) | 812 | 39,894 |
| | FABP4+ macrophage (*CD68* ≥ 2, *FABP4* ≥ 2) | 153 | 3,387 |
| | SPP1+ macrophage (*CD68* ≥ 2, *SPP1* ≥ 2) | 1 | 3,880 |

**Supplementary Figure S12 *In situ* gene expression analysis for the Noguchi type A case (case TSU-20)**

(**a**) Spatial expression patterns of representative cell type markers. The count data is shown as expression levels. (**b**) The result of clustering analysis is represented in the spatial plot (left) and the UMAP plot (right). The cell-type annotation is shown in the margin of the UMAP plot. (**c**) Spatial expression patterns of genes associated with well-differentiated tumor markers. (**d**) Spatial expression patterns of macrophage markers. (**e**) Comparison of the number of macrophages between Noguchi type A and type B tumors. There are almost no SPP1+ macrophages in the Noguchi type A case.

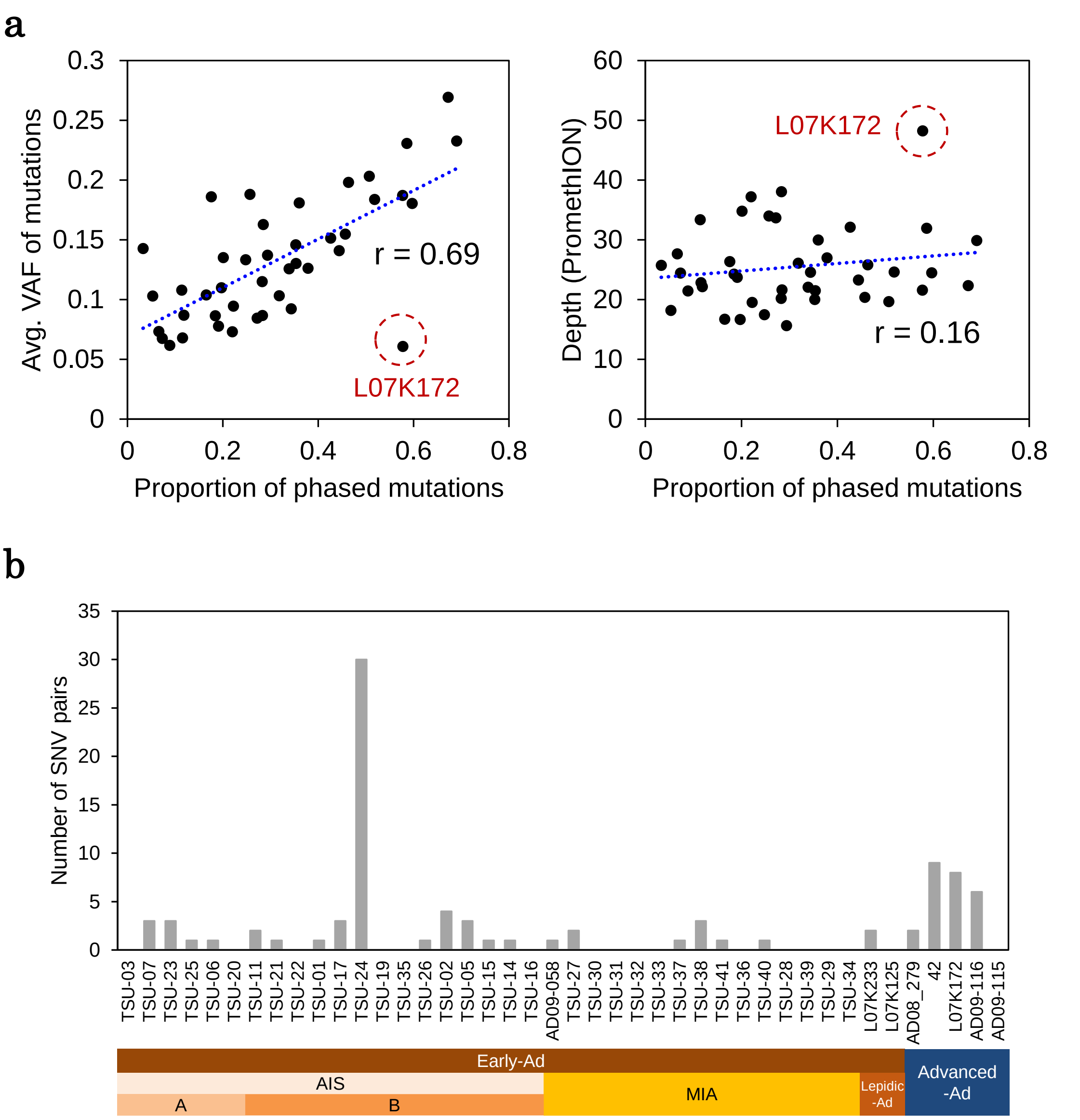


**Supplementary Figure S13 Phasing analysis**

(**a**) Correlation of the proportion of phased mutations with average VAFs (left) and average depth of long read WGS (right) in each case. Pearson's r is shown in the inset. The case L07K172 showed a high proportion of phased mutations as indicated by the high sequencing depth of long read WGS. (**b**) Number of mutation pairs for which the order of occurrence could be resolved. Source data are provided as a Source Data file for **a** and **b**.

# Supplementary Tables

## Supplementary Table S1 Clinical and pathological information of cases of advanced lung adenocarcinoma and other lung cancers

| Case | Classification | Noguchi type | Pathological stage | Histological Predominant | Smoking history | Driver mutation |
|---|---|---|---|---|---|---|
| TSU-03 | AIS | A | 0 | Ad, lepidic | y | *EGFR* L858R |
| TSU-07 | AIS | A | 0 | Ad, lepidic | n | *EGFR* L858R |
| TSU-13 | AIS | A | 0 | Ad, lepidic | n | *EGFR* L858R, L62R |
| TSU-23 | AIS | A | 0 | Ad, lepidic | n | *EGFR* G719A, S768I |
| TSU-25 | AIS | A | 0 | Ad, lepidic | y | *EGFR* L747_P753delinsS |
| TSU-06 | AIS | A | 0 | Ad, lepidic | y | *BRAF* A489_Q493del |
| TSU-10 | AIS | A | 0 | Ad, lepidic | n | *MET* exon 14 splice site |
| TSU-20 | AIS | A | 0 | Ad, lepidic | n | *MAP2K1* E102_I103del |
| AD18-012 | AIS | A | 0 | Ad, lepidic | y | - |
| TSU-09 | AIS | B | 0 | Ad, lepidic | n | *EGFR* L858R |
| TSU-11 | AIS | B | 0 | Ad, lepidic | y | *EGFR* L858R |
| TSU-18 | AIS | B | 0 | Ad, lepidic | y | *EGFR* L858R |
| TSU-21 | AIS | B | 0 | Ad, lepidic | n | *EGFR* L858R |
| TSU-22 | AIS | B | 0 | Ad, lepidic | y | *EGFR* L858R |
| TSU-01 | AIS | B | 0 | Ad, lepidic | y | *EGFR* E746_A750del |
| TSU-17 | AIS | B | 0 | Ad, lepidic | n | *EGFR* E746_A750del |
| TSU-24 | AIS | B | 0 | Ad, lepidic | y | *EGFR* E746_A750del |
| TSU-19 | AIS | B | 0 | Ad, lepidic | y | *EGFR* L747_T751del |
| TSU-35 | AIS | B | 0 | Ad, lepidic | n | *EGFR* L747_E749del, A750P |
| TSU-26 | AIS | B | 0 | Ad, lepidic | n | *EGFR* V769_D770insASV |
| TSU-02 | AIS | B | 0 | Ad, lepidic | n | *EGFR* D770_N771insSVD |
| TSU-05 | AIS | B | 0 | Ad, lepidic | y | *KRAS* G12A |
| TSU-15 | AIS | B | 0 | Ad, lepidic | n | *KIF5B-RET* |
| TSU-12 | AIS | B | 0 | Ad, lepidic | n | - |
| TSU-14 | AIS | B | 0 | Ad, lepidic | n | - |
| TSU-16 | AIS | B | 0 | Ad, lepidic | y | - |
| AD09-058 | MIA | B | IA1 | Ad, lepidic | n | *KRAS* G12D[†] |
| AD18-003 | MIA | B | IA1 | Ad, lepidic | y | - |

| | | | | | | |
|---|---|---|---|---|---|---|
| TSU-27 | MIA | C | IA1 | Ad, lepidic | n | *EGFR* L858R |
| TSU-30 | MIA | C | IA1 | Ad, lepidic | n | *EGFR* L858R |
| TSU-31 | MIA | C | IA1 | Ad, lepidic | y | *EGFR* L858R |
| TSU-32 | MIA | C | IA1 | Ad, lepidic | y | *EGFR* L858R |
| TSU-33 | MIA | C | IA1 | Ad, lepidic | y | *EGFR* L858R |
| TSU-37 | MIA | C | IA1 | Ad, lepidic | n | *EGFR* L858R |
| TSU-38 | MIA | C | IA1 | Ad, lepidic | n | *EGFR* L858R |
| TSU-41 | MIA | C | IA1 | Ad, lepidic | n | *EGFR* L858R |
| TSU-36 | MIA | C | IA1 | Ad, lepidic | n | *EGFR* L861Q |
| TSU-40 | MIA | C | IA1 | Ad, lepidic | n | *EGFR* E746_A750del |
| TSU-28 | MIA | C | IA1 | Ad, lepidic | n | *EGFR* S752_I759del |
| TSU-04 | MIA | C | IA1 | Ad, lepidic | y | *MET* Y1021N |
| TSU-39 | MIA | C | IA1 | Ad, lepidic | y | *MET* exon 14 deletion |
| AD18-015 | MIA | C | IA1 | Ad, lepidic | y | - |
| TSU-29 | MIA | C | IA1 | Ad, lepidic | n | - |
| TSU-34 | MIA | C | IA1 | Ad, lepidic | n | - |
| TSU-08 | Lepidic-Ad | C | IA1 | Ad, lepidic | y | *EGFR* E746_A750del |
| L07K233 | Lepidic-Ad | C | IA1 | Ad, lepidic | y | *EGFR* H773_V774insAH |
| L07K199 | Lepidic-Ad | C | IA3 | Ad, lepidic | n | *ERBB2* V659E |
| L07K125 | Lepidic-Ad | C | IA2 | Ad, lepidic | n | *EML4-ALK*[‡] |
| AD08_279 | Advanced-Ad | *** | IA2 | Ad, papillary | n | *EGFR* L858R |
| AD17-040 | Advanced-Ad | *** | IA1 | Ad, papillary | n | *EGFR* L858R |
| 42 | Advanced-Ad | *** | IA3 | Ad, papillary | y | *EGFR* E746_A750del |
| AD09-104 | Advanced-Ad | *** | IA2 | Ad, acinar | y | *EGFR* H773_V774insAH |
| L07K172 | Advanced-Ad | *** | IA3 | Ad, acinar | n | *EGFR* E709_T710delinsD |
| AD09-044 | Advanced-Ad | *** | IB | Ad, papillary | y | - |
| AD09-116 | Advanced-Ad | *** | IA1 | Ad, papillary | y | - |
| AD09-115 | Advanced-Ad | *** | IIB | Ad, acinar and solid | y | - |
| S9[*] | Advanced-Ad | *** | IA1 | Ad, lepidic | n | *EGFR* L858R |
| S10[*] | Advanced-Ad | *** | IA3 | Ad, papillary | n | *EGFR* L858R |
| S18[*] | Advanced-Ad | *** | IB | Ad, papillary | n | *EGFR* L858R |
| S6[*] | Advanced-Ad | *** | IA3 | Ad, papillary | n | *EGFR* E746_A750del |
| S20[*] | Advanced-Ad | *** | IA3 | Ad, papillary | n | *EGFR* E746_A750del |
| S8[*] | Advanced-Ad | *** | IB | Ad, papillary | n | *EGFR* L747_T751del |
| S19[*] | Advanced-Ad | *** | IA3 | Ad, papillary | y | - |

| | | | | | | |
|---|---|---|---|---|---|---|
| S2* | Advanced-Ad | *** | IIB | Ad, micropapillary | n | *EGFR* L858R |
| S13* | Advanced-Ad | *** | IIIA | Ad, micropapillary | n | *EGFR* L858R |
| S21* | Advanced-Ad | *** | IB | Ad, micropapillary | n | *EGFR* L858R |
| S7* | Advanced-Ad | *** | IA3 | Ad, micropapillary | n | *EGFR* D770_N771insSVD |
| S5* | Advanced-Ad | *** | IIIA | Ad, micropapillary | y | - |
| S15* | Advanced-Ad | *** | IIIA | Ad, micropapillary | y | - |
| S3* | Advanced-Ad | *** | IIB | Ad, solid | y | *NRAS* Q61L |
| S17* | Advanced-Ad | *** | IIIA | Ad, variant | y | - |
| S14* | Others | *** | IIIB | Sq, keratinizing | y | *PIK3CA* E545K, *PTEN* del |
| S11* | Others | *** | IIIB | Sq, keratinizing | y | - |
| S12* | Others | *** | IIB | Pleomorphic carcinoma | y | *KRAS* G12C |
| S1* | Others | *** | IA3 | Large cell carcinoma | y | - |
| S16* | Others | *** | IIB | LCNEC | y | - |

Ad: Adenocarcinoma; Sq: Squamous cell carcinoma.

†These mutations were detected from NCC oncopanel sequencing datasets

*These were previously obtained and analyzed[1,2]

Note: we especially listed mutations in oncogenes previously reported[5–7] as driver aberrations. *KRAS* G12 and *NRAS* Q61 mutations are well-known hotspot mutations[8,9] and *ALK/RET* fusions and *MET* exon 14 mutations were previously reported as driver aberrations[5,7]. For other driver genes, such as *ERBB2*, *BRAF*, and *MAP2K1*, we checked the COSMIC database[10] and the available literature[11–13] to confirm their oncogenic functions.

## Supplementary Table S2 General statistics of whole-genome short read sequencing

| Case | Classification | Noguchi type | Tumor | | Normal | |
|---|---|---|---|---|---|---|
| | | | Mapped read pairs | Depth (×) | Mapped read pairs | Depth (×) |
| TSU-03 | AIS | A | 922,335,607 | 79.2 | 322,232,541 | 28.1 |
| TSU-07 | AIS | A | 1,178,035,168 | 102.5 | 313,353,499 | 26.1 |
| TSU-13 | AIS | A | 1,048,514,386 | 93.6 | 318,488,984 | 27.8 |
| TSU-23 | AIS | A | 1,106,755,909 | 100.6 | 1,142,061,573 | 103.3 |
| TSU-25 | AIS | A | 1,025,242,478 | 92.9 | 1,138,162,827 | 103.1 |
| TSU-06 | AIS | A | 1,074,538,700 | 93.4 | 358,907,561 | 31.2 |
| TSU-10 | AIS | A | 1,048,378,678 | 91.9 | 374,762,665 | 32.9 |
| TSU-20 | AIS | A | 975,599,441 | 88.7 | 1,152,443,298 | 104.6 |
| AD18-012 | AIS | A | 913,015,661 | 80.5 | 279,550,156 | 25.6 |
| TSU-09 | AIS | B | 1,112,647,794 | 96.7 | 352,891,733 | 30.8 |
| TSU-11 | AIS | B | 1,202,093,452 | 104.4 | 354,246,762 | 31.0 |
| TSU-18 | AIS | B | 918,192,612 | 81.9 | 273,804,614 | 24.1 |
| TSU-21 | AIS | B | 901,270,002 | 82.3 | 975,182,539 | 88.2 |
| TSU-22 | AIS | B | 1,137,508,709 | 103.4 | 1,235,250,574 | 112.5 |
| TSU-01 | AIS | B | 1,120,989,158 | 98.6 | 324,830,049 | 28.2 |
| TSU-17 | AIS | B | 1,159,113,785 | 103.6 | 298,409,084 | 26.4 |
| TSU-24 | AIS | B | 1,058,315,245 | 94.7 | 1,335,993,784 | 121.6 |
| TSU-19 | AIS | B | 1,203,790,825 | 109.2 | 868,610,917 | 78.7 |
| TSU-35 | AIS | B | 981,947,196 | 89.2 | 965,234,450 | 87.1 |
| TSU-26 | AIS | B | 988,241,818 | 90.2 | 732,373,720 | 66.3 |
| TSU-02 | AIS | B | 1,183,430,159 | 103.4 | 328,162,278 | 28.5 |
| TSU-05 | AIS | B | 938,113,083 | 81.0 | 317,627,522 | 26.9 |
| TSU-15 | AIS | B | 866,195,780 | 76.6 | 414,650,002 | 36.7 |
| TSU-12 | AIS | B | 1,242,186,651 | 111.0 | 323,190,538 | 28.2 |
| TSU-14 | AIS | B | 1,131,516,564 | 100.9 | 495,983,082 | 43.3 |
| TSU-16 | AIS | B | 1,310,317,039 | 115.8 | 420,658,318 | 37.0 |
| AD09-058 | MIA | B | 1,233,707,578 | 110.8 | 381,481,779 | 34.6 |
| AD18-003 | MIA | B | 1,183,256,837 | 107.0 | 318,454,342 | 27.8 |
| TSU-27 | MIA | C | 1,066,681,176 | 96.9 | 907,045,038 | 82.7 |
| TSU-30 | MIA | C | 1,102,767,685 | 99.0 | 1,334,375,192 | 121.1 |

| | | | | | | |
|---|---|---|---|---|---|---|
| TSU-31 | MIA | C | 1,092,725,883 | 98.2 | 1,008,774,610 | 91.6 |
| TSU-32 | MIA | C | 811,813,913 | 72.7 | 1,188,300,999 | 107.2 |
| TSU-33 | MIA | C | 1,074,170,587 | 95.6 | 1,020,960,201 | 92.6 |
| TSU-37 | MIA | C | 1,081,981,749 | 97.8 | 1,025,917,198 | 92.7 |
| TSU-38 | MIA | C | 978,860,332 | 88.2 | 1,124,304,055 | 101.2 |
| TSU-41 | MIA | C | 964,533,710 | 87.3 | 577,520,978 | 52.1 |
| TSU-36 | MIA | C | 1,133,513,865 | 102.6 | 1,248,897,388 | 112.6 |
| TSU-40 | MIA | C | 858,718,729 | 78.3 | 955,336,073 | 86.4 |
| TSU-28 | MIA | C | 1,216,144,182 | 110.7 | 1,322,751,660 | 119.9 |
| TSU-04 | MIA | C | 972,938,888 | 85.0 | 429,693,173 | 37.3 |
| TSU-39 | MIA | C | 1,119,287,762 | 102.5 | 1,008,839,915 | 92.1 |
| AD18-015 | MIA | C | 1,065,849,587 | 94.7 | 383,908,694 | 34.1 |
| TSU-29 | MIA | C | 1,180,460,306 | 106.3 | 928,043,098 | 84.1 |
| TSU-34 | MIA | C | 1,248,855,621 | 111.7 | 1,170,025,085 | 105.6 |
| TSU-08 | Lepidic-Ad | C | 1,399,161,594 | 127.1 | 301,204,851 | 26.6 |
| L07K233 | Lepidic-Ad | C | 1,190,306,637 | 103.1 | 326,381,074 | 28.2 |
| L07K199 | Lepidic-Ad | C | 1,203,589,922 | 107.9 | 318,425,600 | 28.7 |
| L07K125 | Lepidic-Ad | C | 1,209,127,360 | 111.1 | 395,255,941 | 35.9 |
| Avg. Early-Ad | | | 1,086,182,079 | 97.1 | 689,436,667 | 61.9 |
| AD08_279 | Advanced-Ad | *** | 1,251,784,230 | 112.4 | 366,972,133 | 33.1 |
| AD17-040 | Advanced-Ad | *** | 1,178,555,224 | 107.1 | 476,512,254 | 43.4 |
| 42 | Advanced-Ad | *** | 1,158,220,840 | 106.6 | 442,862,132 | 39.5 |
| AD09-104 | Advanced-Ad | *** | 1,414,095,428 | 128.8 | 295,889,464 | 26.3 |
| L07K172 | Advanced-Ad | *** | 1,227,294,811 | 110.4 | 351,657,932 | 31.8 |
| AD09-044 | Advanced-Ad | *** | 1,159,513,157 | 105.1 | 319,607,929 | 28.5 |
| AD09-115 | Advanced-Ad | *** | 1,110,721,609 | 103.1 | 357,866,527 | 31.9 |
| AD09-116 | Advanced-Ad | *** | 1,164,633,689 | 102.9 | 331,654,558 | 29.4 |
| Avg. Advanced-Ad | | | 1,208,102,374 | 109.6 | 367,877,866 | 33.0 |

Data on 20 Advanced-Ad and other NSCLC cases (S1-S3, S5-S21) previously obtained and analyzed[1,2] were also used here.

## Supplementary Table S3 General statistics of whole-genome long read sequencing

| Case | Classification | Noguchi type | Tumor | | | | | Normal | | | | |
|---|---|---|---|---|---|---|---|---|---|---|---|---|
| | | | 1d reads | Yields (Gb) | Depth (×) | Avg. read length | N50 read length | 1d reads | Yields (Gb) | Depth (×) | Avg. read length | N50 read length |
| TSU-03 | AIS | A | 6,012,636 | 54.3 | 18.2 | 9,031 | 26,153 | 13,511,590 | 124.6 | 41.7 | 9,220 | 23,782 |
| TSU-07 | AIS | A | 5,802,042 | 64.6 | 21.6 | 11,136 | 32,537 | *** | *** | *** | *** | *** |
| TSU-23 | AIS | A | 3,773,102 | 58.3 | 19.5 | 15,463 | 30,917 | 8,719,401 | 83.1 | 27.8 | 9,532 | 16,192 |
| TSU-25 | AIS | A | 4,927,922 | 64.4 | 21.5 | 13,066 | 24,974 | 5,934,998 | 74.8 | 25.0 | 12,602 | 24,627 |
| TSU-06 | AIS | A | 9,378,281 | 89.2 | 29.8 | 9,510 | 22,510 | 43,854,903 | 126.3 | 42.3 | 2,881 | 11,916 |
| TSU-20 | AIS | A | 5,033,248 | 73.5 | 24.6 | 14,596 | 27,842 | 4,097,148 | 63.8 | 21.4 | 15,580 | 25,267 |
| TSU-11 | AIS | B | 8,675,171 | 80.6 | 27.0 | 9,295 | 22,920 | 10,168,961 | 81.1 | 27.1 | 7,975 | 22,720 |
| TSU-21 | AIS | B | 4,282,718 | 60.4 | 20.2 | 14,092 | 27,051 | 5,940,025 | 86.0 | 28.8 | 14,472 | 24,499 |
| TSU-22 | AIS | B | 4,978,266 | 65.9 | 22.0 | 13,230 | 27,032 | 8,076,561 | 63.7 | 21.3 | 7,881 | 16,551 |
| TSU-01 | AIS | B | 11,099,766 | 89.5 | 29.9 | 8,061 | 23,837 | *** | *** | *** | *** | *** |
| TSU-17 | AIS | B | 10,542,203 | 95.9 | 32.1 | 9,101 | 22,142 | 8,146,748 | 89.0 | 29.8 | 10,927 | 28,279 |
| TSU-24 | AIS | B | 4,150,946 | 66.7 | 22.3 | 16,069 | 27,652 | 4,921,466 | 74.6 | 25.0 | 15,155 | 27,181 |
| TSU-19 | AIS | B | 5,579,061 | 70.8 | 23.7 | 12,686 | 25,059 | 8,852,893 | 74.0 | 24.8 | 8,362 | 16,849 |
| TSU-35 | AIS | B | 3,092,238 | 49.8 | 16.7 | 16,114 | 27,244 | 13,639,065 | 98.6 | 33.0 | 7,227 | 12,276 |
| TSU-26 | AIS | B | 5,473,897 | 69.5 | 23.2 | 12,689 | 24,766 | 5,177,836 | 80.7 | 27.0 | 15,582 | 24,479 |
| TSU-02 | AIS | B | 9,821,450 | 95.4 | 31.9 | 9,710 | 24,453 | 11,307,022 | 91.5 | 30.6 | 8,095 | 20,523 |
| TSU-05 | AIS | B | 14,609,466 | 100.6 | 33.7 | 6,887 | 20,412 | 10,689,979 | 98.9 | 33.1 | 9,256 | 23,679 |
| TSU-15 | AIS | B | 19,133,000 | 113.7 | 38.0 | 5,942 | 18,503 | 11,814,642 | 115.7 | 38.7 | 9,790 | 25,219 |
| TSU-14 | AIS | B | 12,306,241 | 103.9 | 34.8 | 8,443 | 20,957 | *** | *** | *** | *** | *** |
| TSU-16 | AIS | B | 7,025,466 | 64.0 | 21.4 | 9,113 | 23,614 | 7,234,038 | 34.9 | 11.7 | 4,828 | 13,063 |
| AD09-058 | MIA | B | 12,654,436 | 111.1 | 37.2 | 8,777 | 20,628 | 14,763,439 | 130.6 | 43.7 | 8,844 | 23,572 |
| TSU-27 | MIA | C | 3,864,129 | 59.7 | 20.0 | 15,450 | 25,755 | 3,493,175 | 53.0 | 17.7 | 15,176 | 25,829 |
| TSU-30 | MIA | C | 9,024,698 | 73.3 | 24.5 | 8,125 | 12,261 | 5,135,113 | 64.0 | 21.4 | 12,466 | 20,546 |
| TSU-31 | MIA | C | 3,978,096 | 60.9 | 20.4 | 15,308 | 26,340 | 6,565,264 | 73.2 | 24.5 | 11,147 | 18,513 |
| TSU-32 | MIA | C | 4,440,524 | 52.1 | 17.4 | 11,732 | 20,003 | 5,051,910 | 69.4 | 23.2 | 13,730 | 24,424 |
| TSU-33 | MIA | C | 5,747,524 | 77.9 | 26.1 | 13,547 | 22,698 | 5,597,496 | 76.7 | 25.7 | 13,711 | 25,479 |
| TSU-37 | MIA | C | 5,260,887 | 68.2 | 22.8 | 12,958 | 22,747 | 7,168,511 | 71.1 | 23.8 | 9,915 | 19,887 |
| TSU-38 | MIA | C | 5,429,216 | 73.1 | 24.5 | 13,471 | 23,374 | 5,355,957 | 74.1 | 24.8 | 13,827 | 24,054 |
| TSU-41 | MIA | C | 5,892,878 | 72.4 | 24.2 | 12,294 | 22,134 | 7,334,555 | 76.1 | 25.5 | 10,376 | 17,676 |

| | | | | | | | | | | | | |
|---|---|---|---|---|---|---|---|---|---|---|---|---|
| TSU-36 | MIA | C | 3,435,147 | 46.7 | 15.6 | 13,600 | 23,633 | 5,309,987 | 80.3 | 26.9 | 15,121 | 26,804 |
| TSU-40 | MIA | C | 4,950,136 | 58.7 | 19.7 | 11,864 | 22,967 | 5,859,023 | 72.1 | 24.1 | 12,302 | 21,943 |
| TSU-28 | MIA | C | 3,445,814 | 49.8 | 16.7 | 14,451 | 24,247 | 4,538,673 | 75.1 | 25.1 | 16,542 | 24,998 |
| TSU-39 | MIA | C | 5,739,380 | 64.1 | 21.5 | 11,170 | 19,637 | 4,771,139 | 53.1 | 17.8 | 11,131 | 21,009 |
| TSU-29 | MIA | C | 4,657,276 | 66.2 | 22.2 | 14,220 | 22,444 | 4,460,070 | 69.7 | 23.3 | 15,637 | 24,199 |
| TSU-34 | MIA | C | 4,961,192 | 73.0 | 24.4 | 14,718 | 25,921 | 4,781,651 | 80.8 | 27.0 | 16,902 | 27,639 |
| L07K233 | Lepidic-Ad | C | 8,152,588 | 77.2 | 25.8 | 9,465 | 26,750 | 15,539,876 | 95.9 | 32.1 | 6,172 | 16,212 |
| L07K125 | Lepidic-Ad | C | 6,853,921 | 82.6 | 27.7 | 12,057 | 28,661 | 18,734,193 | 119.1 | 39.9 | 6,358 | 16,650 |
| Avg. Early-Ad | | | 6,869,864 | 72.9 | 24.4 | 11,823 | 24,075 | 9,016,097 | 82.2 | 27.5 | 11,139 | 21,663 |
| AD08_279 | Advanced-Ad | *** | 17,182,498 | 99.7 | 33.4 | 5,803 | 29,079 | 7,222,097 | 93.0 | 31.1 | 12,883 | 14,081 |
| 42 | Advanced-Ad | *** | 10,220,367 | 101.6 | 34.0 | 9,939 | 29,583 | 8,852,353 | 79.3 | 26.6 | 8,964 | 22,627 |
| L07K172 | Advanced-Ad | *** | 14,458,496 | 144.0 | 48.2 | 7,810 | 21,327 | 14,567,636 | 113.8 | 38.1 | 7,810 | 21,327 |
| AD09-115 | Advanced-Ad | *** | 10,486,393 | 78.7 | 26.4 | 7,509 | 26,347 | 25,323,035 | 132.1 | 44.2 | 5,215 | 20,950 |
| AD09-116 | Advanced-Ad | *** | 14,957,010 | 76.8 | 25.7 | 5,133 | 10,100 | 21,190,778 | 132.7 | 44.4 | 6,262 | 15,403 |
| Avg. Advanced-Ad | | | 13,460,953 | 100.2 | 33.5 | 7,239 | 23,287 | 15,431,180 | 110.2 | 36.9 | 8,227 | 18,878 |

Data on 20 Advanced-Ad and other NSCLC cases (S1-S3, S5-S21) previously obtained and analyzed[1,2] were also used here.

## Supplementary Table S4 DNA methylation patterns profiled from long read data

### (a) Tumor

| Case | Classification | Noguchi type | Tumor | | | | |
|---|---|---|---|---|---|---|---|
| | | | Number of CpG sites | Number of CpG sites (≥5 reads) | | | |
| | | | | Total | 0-25% | 25-75% | 75-100% |
| TSU-03 | AIS | A | 28,153,144 | 26,022,528 | 2,780,489 | 6,584,355 | 16,657,684 |
| TSU-07* | AIS | A | 28,207,539 | 26,973,943 | 2,961,465 | 5,843,207 | 18,169,271 |
| TSU-23 | AIS | A | 28,341,079 | 25,257,745 | 2,600,356 | 6,759,849 | 15,897,540 |
| TSU-25 | AIS | A | 28,215,288 | 25,904,713 | 2,845,676 | 7,958,241 | 15,100,796 |
| TSU-06 | AIS | A | 28,232,763 | 27,641,028 | 3,085,230 | 6,084,224 | 18,471,574 |
| TSU-20 | AIS | A | 28,237,956 | 27,097,739 | 2,979,218 | 6,634,674 | 17,483,847 |
| TSU-11 | AIS | B | 28,196,551 | 27,506,890 | 2,817,707 | 5,300,041 | 19,389,142 |
| TSU-21 | AIS | B | 28,327,191 | 25,261,606 | 2,683,757 | 7,296,039 | 15,281,810 |
| TSU-22 | AIS | B | 28,232,781 | 26,700,422 | 2,785,130 | 7,269,586 | 16,645,706 |
| TSU-01* | AIS | B | 28,116,344 | 27,645,523 | 3,034,474 | 6,221,295 | 18,389,754 |
| TSU-17 | AIS | B | 28,087,454 | 27,712,333 | 2,803,944 | 5,220,250 | 19,688,139 |
| TSU-24 | AIS | B | 28,326,349 | 25,088,072 | 4,004,058 | 6,252,307 | 14,831,707 |
| TSU-19 | AIS | B | 28,372,605 | 26,833,714 | 2,690,058 | 7,124,908 | 17,018,748 |
| TSU-35 | AIS | B | 28,190,821 | 23,708,664 | 2,319,800 | 6,606,385 | 14,782,479 |
| TSU-26 | AIS | B | 28,222,387 | 26,764,297 | 2,553,049 | 7,141,136 | 17,070,112 |
| TSU-02 | AIS | B | 28,111,956 | 27,677,694 | 2,846,581 | 5,532,866 | 19,298,247 |
| TSU-05 | AIS | B | 28,212,397 | 27,606,823 | 2,808,481 | 6,889,653 | 17,908,689 |
| TSU-15 | AIS | B | 28,133,251 | 27,842,033 | 2,804,275 | 6,528,167 | 18,509,591 |
| TSU-14* | AIS | B | 28,124,486 | 27,840,812 | 2,793,645 | 6,508,103 | 18,539,064 |
| TSU-16 | AIS | B | 28,146,099 | 26,207,706 | 2,870,842 | 6,063,053 | 17,273,811 |
| TSU-27 | MIA | C | 28,211,740 | 25,803,726 | 2,902,075 | 8,236,796 | 14,664,855 |
| TSU-30 | MIA | C | 28,222,231 | 27,084,355 | 2,714,397 | 7,553,810 | 16,816,148 |
| TSU-31 | MIA | C | 28,330,723 | 25,542,278 | 2,897,550 | 7,300,274 | 15,344,454 |
| TSU-32 | MIA | C | 28,302,606 | 21,800,119 | 2,163,273 | 6,538,761 | 13,098,085 |
| TSU-33 | MIA | C | 28,231,736 | 27,215,608 | 3,088,742 | 9,569,332 | 14,557,534 |
| TSU-37 | MIA | C | 28,215,584 | 26,851,711 | 2,890,477 | 8,860,894 | 15,100,340 |
| TSU-38 | MIA | C | 28,234,114 | 27,079,673 | 2,734,093 | 7,454,775 | 16,890,805 |
| TSU-41 | MIA | C | 28,228,783 | 26,977,913 | 2,777,855 | 7,029,228 | 17,170,830 |

| | | | | | | | | |
|---|---|---|---|---|---|---|---|---|
| TSU-36 | MIA | C | 28,201,185 | 22,918,466 | 2,691,733 | 6,614,619 | 13,612,114 |
| TSU-40 | MIA | C | 28,209,736 | 25,180,323 | 2,980,119 | 6,622,835 | 15,577,369 |
| TSU-28 | MIA | C | 28,175,348 | 23,032,535 | 2,317,893 | 6,533,600 | 14,181,042 |
| TSU-39 | MIA | C | 28,171,684 | 24,862,999 | 2,640,039 | 8,452,137 | 13,770,823 |
| TSU-29 | MIA | C | 28,205,481 | 26,651,818 | 2,806,468 | 7,236,218 | 16,609,132 |
| TSU-34 | MIA | C | 28,228,983 | 27,231,647 | 2,858,151 | 7,519,236 | 16,854,260 |
| L07K233 | Lepedic-Ad | C | 28,202,082 | 26,996,129 | 2,748,119 | 6,024,729 | 18,223,281 |
| L07K125 | Lepedic-Ad | C | 28,090,137 | 27,164,200 | 2,916,280 | 6,967,669 | 17,280,251 |
| 42 | Advanced-Ad | *** | 28,234,407 | 27,616,640 | 2,904,570 | 8,258,463 | 16,453,607 |
| L07K172 | Advanced-Ad | *** | 28,074,971 | 26,831,086 | 3,083,183 | 7,736,375 | 16,011,528 |
| AD09-116 | Advanced-Ad | *** | 28,187,105 | 26,637,180 | 3,182,244 | 8,491,243 | 14,963,693 |
| AD09-115 | Advanced-Ad | *** | 28,165,863 | 26,610,806 | 2,941,688 | 7,831,278 | 15,837,840 |
| S9† | Advanced-Ad | *** | 28,133,602 | 27,849,871 | 3,151,829 | 5,376,757 | 19,321,285 |
| S10† | Advanced-Ad | *** | 28,032,169 | 26,657,216 | 3,619,939 | 9,325,991 | 13,711,286 |
| S18† | Advanced-Ad | *** | 28,032,332 | 25,756,576 | 2,908,684 | 7,130,113 | 15,717,779 |
| S8† | Advanced-Ad | *** | 28,021,424 | 26,642,627 | 3,970,284 | 7,558,249 | 15,114,094 |
| S6† | Advanced-Ad | *** | 28,027,073 | 26,375,130 | 2,985,242 | 6,262,740 | 17,127,148 |
| S20† | Advanced-Ad | *** | 28,144,276 | 26,489,511 | 4,351,842 | 8,721,088 | 13,416,581 |
| S19† | Advanced-Ad | *** | 28,199,749 | 27,535,504 | 2,995,028 | 6,977,697 | 17,562,779 |
| S2† | Advanced-Ad | *** | 28,036,860 | 27,096,813 | 3,504,669 | 6,439,915 | 17,152,229 |
| S13† | Advanced-Ad | *** | 28,064,949 | 27,690,891 | 2,977,748 | 6,982,119 | 17,731,024 |
| S21† | Advanced-Ad | *** | 28,069,556 | 27,638,058 | 3,236,509 | 8,573,453 | 15,828,096 |
| S7† | Advanced-Ad | *** | 27,951,791 | 25,236,399 | 2,898,223 | 6,214,110 | 16,124,066 |
| S5† | Advanced-Ad | *** | 28,160,267 | 27,033,985 | 3,009,573 | 7,454,997 | 16,569,415 |
| S15† | Advanced-Ad | *** | 28,205,078 | 27,645,233 | 3,034,276 | 8,011,569 | 16,599,388 |
| S3† | Advanced-Ad | *** | 28,109,637 | 25,915,581 | 3,778,529 | 10,637,262 | 11,499,790 |
| S17† | Advanced-Ad | *** | 28,116,616 | 26,792,366 | 2,923,070 | 7,515,649 | 16,353,647 |
| S14† | Others | *** | 28,060,141 | 27,100,064 | 11,171,629 | 6,308,869 | 9,619,566 |
| S11† | Others | *** | 28,130,197 | 27,405,999 | 4,397,751 | 10,931,113 | 12,077,135 |
| S12† | Others | *** | 28,206,905 | 27,802,412 | 2,860,842 | 6,949,726 | 17,991,844 |
| S1† | Others | *** | 28,038,203 | 27,275,785 | 3,301,597 | 11,130,799 | 12,843,389 |
| S16† | Others | *** | 27,927,904 | 25,546,371 | 6,963,593 | 7,018,621 | 11,564,157 |

## (b) Normal

| Case | Classification | Noguchi type | Tumor | | | | |
|---|---|---|---|---|---|---|---|
| | | | Number of CpG sites | Number of CpG sites (≥5 reads) | | | |
| | | | | Total | 0-25% | 25-75% | 75-100% |
| TSU-03 | AIS | A | 28,261,902 | 27,959,933 | 3,019,362 | 6,448,434 | 18,492,137 |
| TSU-07* | AIS | A | *** | *** | *** | *** | *** |
| TSU-23 | AIS | A | 28,359,494 | 28,082,741 | 2,913,361 | 6,288,153 | 18,881,227 |
| TSU-25 | AIS | A | 28,235,925 | 27,998,331 | 2,870,724 | 6,361,021 | 18,766,586 |
| TSU-06 | AIS | A | 28,284,007 | 27,971,213 | 2,933,585 | 6,588,605 | 18,449,023 |
| TSU-20 | AIS | A | 28,228,094 | 27,980,062 | 2,951,727 | 6,319,023 | 18,709,312 |
| TSU-11 | AIS | B | 28,176,637 | 26,346,601 | 2,908,325 | 6,082,550 | 17,355,726 |
| TSU-21 | AIS | B | 28,370,993 | 28,118,174 | 2,917,490 | 6,351,492 | 18,849,192 |
| TSU-22 | AIS | B | 28,189,694 | 27,863,128 | 2,804,053 | 6,850,221 | 18,208,854 |
| TSU-01* | AIS | B | *** | *** | *** | *** | *** |
| TSU-17 | AIS | B | 28,122,577 | 27,696,303 | 2,919,261 | 6,525,006 | 18,252,036 |
| TSU-24 | AIS | B | 28,383,590 | 28,094,765 | 2,904,749 | 6,359,869 | 18,830,147 |
| TSU-19 | AIS | B | 28,381,219 | 28,060,339 | 2,896,239 | 6,902,586 | 18,261,514 |
| TSU-35 | AIS | B | 28,272,518 | 27,993,276 | 2,896,357 | 7,142,187 | 17,954,732 |
| TSU-26 | AIS | B | 28,212,984 | 28,008,201 | 2,901,295 | 6,481,452 | 18,625,454 |
| TSU-02 | AIS | B | 28,117,750 | 27,665,722 | 2,903,656 | 6,822,800 | 17,939,266 |
| TSU-05 | AIS | B | 28,246,843 | 27,855,638 | 2,930,580 | 6,319,385 | 18,605,673 |
| TSU-15 | AIS | B | 28,145,522 | 27,876,613 | 2,915,598 | 6,304,347 | 18,656,668 |
| TSU-14* | AIS | B | *** | *** | *** | *** | *** |
| TSU-16 | AIS | B | 27,840,800 | 17,647,344 | 1,967,620 | 3,500,179 | 12,179,545 |
| TSU-27 | MIA | C | 28,211,439 | 27,882,231 | 2,861,547 | 6,245,778 | 18,774,906 |
| TSU-30 | MIA | C | 28,216,528 | 27,957,590 | 2,892,722 | 6,516,009 | 18,548,859 |
| TSU-31 | MIA | C | 28,362,592 | 28,066,378 | 2,959,033 | 6,264,907 | 18,842,438 |
| TSU-32 | MIA | C | 28,343,962 | 28,040,715 | 2,899,450 | 6,209,165 | 18,932,100 |
| TSU-33 | MIA | C | 28,241,953 | 28,015,382 | 2,791,376 | 6,709,627 | 18,514,379 |
| TSU-37 | MIA | C | 28,231,667 | 27,969,447 | 2,899,274 | 7,017,702 | 18,052,471 |
| TSU-38 | MIA | C | 28,245,391 | 28,009,105 | 2,845,959 | 6,026,661 | 19,136,485 |
| TSU-41 | MIA | C | 28,249,754 | 27,300,465 | 2,812,540 | 6,927,839 | 17,560,086 |
| TSU-36 | MIA | C | 28,255,504 | 28,039,308 | 2,887,012 | 6,359,935 | 18,792,361 |

| | | | | | | | |
|---|---|---|---|---|---|---|---|
| TSU-40 | MIA | C | 28,238,557 | 27,994,993 | 2,810,407 | 5,969,666 | 19,214,920 |
| TSU-28 | MIA | C | 28,219,303 | 28,001,160 | 2,863,034 | 5,977,868 | 19,160,258 |
| TSU-39 | MIA | C | 28,193,002 | 27,836,324 | 2,830,004 | 7,610,022 | 17,396,298 |
| TSU-29 | MIA | C | 28,226,551 | 27,983,987 | 2,919,254 | 6,703,801 | 18,360,932 |
| TSU-34 | MIA | C | 28,237,304 | 28,014,366 | 2,879,840 | 6,166,215 | 18,968,311 |
| L07K233 | Lepedic-Ad | C | 28,207,028 | 27,538,540 | 2,883,399 | 6,295,943 | 18,359,198 |
| L07K125 | Lepedic-Ad | C | 28,098,252 | 27,796,955 | 2,931,023 | 6,730,034 | 18,135,898 |
| 42 | Advanced-Ad | *** | 28,167,374 | 26,475,754 | 3,014,143 | 6,579,723 | 16,881,888 |
| L07K172 | Advanced-Ad | *** | 28,121,320 | 27,797,866 | 2,926,784 | 6,345,078 | 18,526,004 |
| AD09-116 | Advanced-Ad | *** | 28,287,570 | 27,995,376 | 2,911,637 | 6,558,035 | 18,525,704 |
| AD09-115 | Advanced-Ad | *** | 28,243,718 | 27,951,325 | 2,847,604 | 6,327,045 | 18,776,676 |
| S9[†] | Advanced-Ad | *** | 28,089,031 | 27,528,863 | 2,995,095 | 6,287,680 | 18,246,088 |
| S10[†] | Advanced-Ad | *** | 27,767,663 | 20,065,282 | 2,532,525 | 4,457,940 | 13,074,817 |
| S18[†] | Advanced-Ad | *** | 28,059,806 | 27,658,021 | 3,034,270 | 6,735,022 | 17,888,729 |
| S8[†] | Advanced-Ad | *** | 27,920,932 | 23,601,666 | 2,869,209 | 5,663,663 | 15,068,794 |
| S6[†] | Advanced-Ad | *** | 27,800,824 | 20,892,470 | 2,624,922 | 4,499,874 | 13,767,674 |
| S20[†] | Advanced-Ad | *** | 28,013,367 | 23,245,385 | 2,802,883 | 5,373,936 | 15,068,566 |
| S19[†] | Advanced-Ad | *** | 28,171,733 | 27,421,932 | 2,891,468 | 5,999,399 | 18,531,065 |
| S2[†] | Advanced-Ad | *** | 27,713,111 | 18,726,913 | 2,438,512 | 4,242,608 | 12,045,793 |
| S13[†] | Advanced-Ad | *** | 27,788,127 | 19,269,550 | 2,321,715 | 4,352,025 | 12,595,810 |
| S21[†] | Advanced-Ad | *** | 27,979,916 | 25,675,930 | 2,793,581 | 6,344,255 | 16,538,094 |
| S7[†] | Advanced-Ad | *** | 27,753,842 | 19,156,481 | 2,469,021 | 4,430,874 | 12,256,586 |
| S5[†] | Advanced-Ad | *** | 27,757,176 | 17,587,129 | 2,070,632 | 3,560,046 | 11,956,451 |
| S15[†] | Advanced-Ad | *** | 28,099,665 | 25,250,975 | 2,961,465 | 5,766,994 | 16,522,516 |
| S3[†] | Advanced-Ad | *** | 27,794,490 | 20,301,079 | 2,486,937 | 5,089,828 | 12,724,314 |
| S17[†] | Advanced-Ad | *** | 28,105,766 | 25,395,076 | 2,884,956 | 5,985,312 | 16,524,808 |
| S14[†] | Others | *** | 28,138,316 | 26,979,517 | 2,904,187 | 6,198,044 | 17,877,286 |
| S11[†] | Others | *** | 28,101,826 | 26,036,811 | 2,858,816 | 4,706,911 | 18,471,084 |
| S12[†] | Others | *** | 28,178,140 | 27,252,793 | 3,003,869 | 6,369,877 | 17,879,047 |
| S1[†] | Others | *** | 27,972,623 | 25,737,078 | 2,828,241 | 6,382,108 | 16,526,729 |
| S16[†] | Others | *** | 27,964,141 | 22,707,556 | 2,563,215 | 5,664,834 | 14,479,507 |

[*]Long read data of three cases only obtained from tumor tissues

[†]These data were previously obtained and analyzed[1,2]

Note: DNA methylation data of AD08_279 and AD09-058 not available because the FAST5 files were accidently broken.

**Supplementary Table S5 General RNA-seq statistics**

| Case | Classification | Noguchi type | Total read pairs | Uniquely mapped read pairs | Method |
|---|---|---|---|---|---|
| TSU-13 | AIS | A | 20,237,503 | 19,500,952 | Poly-A† |
| TSU-06 | AIS | A | 25,623,933 | 24,223,042 | Poly-A† |
| TSU-10 | AIS | A | 18,897,022 | 17,937,064 | Poly-A† |
| AD18-012 | AIS | A | 72,593,427 | 62,502,385 | SMART-seq |
| TSU-11 | AIS | B | 21,742,690 | 20,866,455 | Poly-A† |
| TSU-18 | AIS | B | 21,259,283 | 19,916,262 | Poly-A† |
| TSU-01 | AIS | B | 25,095,979 | 23,623,377 | Poly-A† |
| TSU-17 | AIS | B | 25,309,240 | 24,435,047 | Poly-A† |
| TSU-02 | AIS | B | 22,674,975 | 21,176,270 | Poly-A† |
| TSU-05 | AIS | B | 19,161,174 | 17,414,229 | Poly-A† |
| TSU-14 | AIS | B | 17,952,542 | 17,207,619 | Poly-A† |
| AD09-058 | MIA | B | 56,417,008 | 30,860,991 | RIBOZERO |
| AD18-003 | MIA | B | 73,827,196 | 51,901,845 | RIBOZERO |
| AD18-015 | MIA | C | 70,414,466 | 41,263,967 | RIBOZERO |
| TSU-08 | Lepidic-Ad | C | 23,050,361 | 22,008,142 | Poly-A† |
| L07K233 | Lepidic-Ad | C | 27,972,474 | 25,853,753 | Poly-A† |
| L07K199 | Lepidic-Ad | C | 59,822,466 | 35,399,313 | RIBOZERO |
| L07K125 | Lepidic-Ad | C | 50,536,202 | 20,709,447 | RIBOZERO |
| AD08_279 | Advanced-Ad | *** | 20,152,890 | 18,890,978 | Poly-A† |
| AD17-040 | Advanced-Ad | *** | 58,421,226 | 54,484,948 | RIBOZERO |
| 42 | Advanced-Ad | *** | 23,676,292 | 22,473,900 | Poly-A† |
| AD09-104 | Advanced-Ad | *** | 23,623,815 | 22,659,209 | Poly-A† |
| L07K172 | Advanced-Ad | *** | 22,534,520 | 21,245,157 | Poly-A† |
| AD09-044 | Advanced-Ad | *** | 75,966,507 | 64,498,168 | Poly-A† |
| AD09-116 | Advanced-Ad | *** | 53,694,501 | 25,928,091 | RIBOZERO |
| AD09-115 | Advanced-Ad | *** | 20,627,259 | 19,373,067 | Poly-A† |

Poly-A: TruSeq Stranded mRNA Library Prep; RIBOZERO: TruSeq Stranded Total RNA Library Prep Gold; SMART-seq: SMART-Seq Stranded Kit.

*Total: 28,278 protein-coding genes.

†Expression analysis performed only using datasets from the Poly-A method.

**Supplementary Table S6 General statistics of the spatial transcriptome analysis with Visium**

| Case | TSU-20 | TSU-21 | TSU-33 |
|---|---|---|---|
| Classification | AIS | AIS | MIA |
| Noguchi type | A | B | C |
| Number of spots | 771 | 3,630 | 2,594 |
| Number of reads | 520,375,172 | 212,891,961 | 396,273,370 |
| Mean reads per spot | 674,935 | 58,648 | 152,765 |
| Median genes per spot | 4,302 | 2,236 | 1,505 |
| Median UMI counts per spot | 11,457 | 4,336 | 2,464 |
| Sequencing saturation | 0.97 | 0.87 | 0.96 |

**Supplementary Table S7 General information for multiplexed fluorescence immunostaining by PhenoCycler**

**(a) List of antibodies, barcodes, and reporters**

PhenoCycler inventory

| Molecule | Conjugated-antibody catalog number | Clone | Barcode | Florescence |
|---|---|---|---|---|
| CD19 | 4550099 | HIB19 | BX003 | AF647 |
| CD8 | 4150004 | SK1 | BX004 | AF488 |
| LIF | 4450032 | M1506B09 | BX006 | Cy5 |
| CD279/PD-1 | 4250010 | EH12.2H7 | BX014 | Atto 550 |
| CD68 | 4350019 | KP1 | BX015 | Cy5 |
| CD45RO | 4250023 | UCHL1 | BX017 | Atto 550 |
| Pan-Cytokeratin | 4150020 | AE-1/AE-3 | BX019 | AF488 |
| β-Catenin1 | 4450036 | 12F7 | BX020 | Atto 550 |
| CD4 | 4350010 | SK3 | BX021 | Cy5 |
| Podoplanin | 4250004 | NC-08 | BX023 | Atto 550 |
| CD11c | 4550107 | S-HCL-3 | BX027 | AF647 |
| CD31 | 4250009 | WM59 | BX032 | Atto 550 |
| Mac2/Galectin-3 | 4450034 | M3/38 | BX035 | Atto 550 |
| Ki67 | 4250019 | B56 | BX047 | Atto 550 |
| Caveolin | 4550084 | D46G3 | BX086 | AF647 |

Custom

| Molecule | Antibody catalog number | Clone | Barcode | Florescence |
|---|---|---|---|---|
| CD163 | #25121 (CST) | D6U1J | BX028 | AF488 |
| BCA1/CXCL13 | ab270408 (Abcam) | EPR23400-92 | BX030 | Cy5 |
| CTLA4 | #26893 (CST) | E2V1Z | BX033 | Cy5 |
| CD274/PD-L1 | #85164 (CST) | E1L3N | BX041 | Atto 550 |
| TTF-1/NKX2-1 | ab242428 (Abcam) | SP141 | BX050 | AF647 |
| FOXP3 | #14-4777-82 (Invitrogen) | 236A/E7 | BX052 | Atto 550 |
| α-SMA/ACTA2 | ab7817 (Abcam) | 1A4 | BX054 | Cy5 |

## (b) Run design

| Cycle | DAPI | Atto 550 | | Cy5/AF647 | | AF488 | |
|---|---|---|---|---|---|---|---|
| 1 | DAPI | Blank | --- | Blank | --- | Blank | --- |
| 2 | DAPI | RX014 | CD279/PD-1 | RX003 | CD19 | RX004 | CD8 |
| 3 | DAPI | RX017 | CD45RO | RX006 | LIF | RX019 | Pan-Cytokeratin |
| 4 | DAPI | RX020 | β-Catenin1 | RX015 | CD68 | RX028 | CD163 |
| 5 | DAPI | RX023 | Podoplanin | RX021 | CD4 | Empty | --- |
| 6 | DAPI | RX032 | CD31 | RX027 | CD11c | Empty | --- |
| 7 | DAPI | RX035 | Mac2/Galectin-3 | RX030 | BCA1/CXCL13 | Empty | --- |
| 8 | DAPI | RX041 | CD274/PD-L1 | RX033 | CTLA4 | Empty | --- |
| 9 | DAPI | RX047 | Ki67 | RX050 | TTF1 | Empty | --- |
| 10 | DAPI | RX052 | FOXP3 | RX054 | α-SMA/ACTA2 | Empty | --- |
| 11 | DAPI | Empty | --- | RX086 | Caveolin | Empty | --- |
| 12 | DAPI | Blank | --- | Blank | --- | Blank | --- |

**Supplementary Table S8 General statistics of *in situ* gene expression profiling Xenium**

| | TSU-20 | TSU-21 |
|---|---|---|
| Classification | AIS | AIS |
| Noguchi type | A | B |
| Region area (μm$^2$) | 11,382,582 | 66,611,240 |
| Total cell area (μm$^2$) | 5,506,283 | 46,327,927 |
| Fraction of transcripts decoded q20 | 0.95 | 0.89 |
| Decoded transcripts per 100 μm$^2$ | 28.2 | 96.6 |
| Negative control probe rate | 0.00069 | 0.020 |
| Negative control codeword rate | 0.00026 | 0.0091 |
| Number of cells detected | 58,648 | 351,742 |
| Median genes per cell | 13 | 46 |
| Median transcripts per cell | 20 | 105 |

**Supplementary Table S9 Results of phasing analysis**

| Case | Classification | Noguchi type | Number of blocks | N50 block length (bp) | Number of phased SNPs |
|---|---|---|---|---|---|
| TSU-03 | AIS | A | 4,064 | 1,292,554 | 1,843,350 |
| TSU-07 | AIS | A | 3,217 | 1,722,864 | 1,836,097 |
| TSU-23 | AIS | A | 4,080 | 1,229,019 | 1,695,993 |
| TSU-25 | AIS | A | 5,510 | 1,005,603 | 1,838,619 |
| TSU-06 | AIS | A | 3,183 | 1,642,965 | 1,846,192 |
| TSU-20 | AIS | A | 4,004 | 1,393,481 | 1,851,306 |
| TSU-11 | AIS | B | 4,127 | 1,261,984 | 1,837,586 |
| TSU-21 | AIS | B | 4,884 | 1,079,220 | 1,798,831 |
| TSU-22 | AIS | B | 5,628 | 962,161 | 1,828,245 |
| TSU-01 | AIS | B | 3,457 | 1,590,894 | 1,890,520 |
| TSU-17 | AIS | B | 4,323 | 1,239,317 | 1,883,337 |
| TSU-24 | AIS | B | 5,912 | 904,857 | 1,856,013 |
| TSU-19 | AIS | B | 4,779 | 1,129,480 | 1,817,040 |
| TSU-35 | AIS | B | 6,120 | 889,273 | 1,851,180 |
| TSU-26 | AIS | B | 5,095 | 1,078,180 | 1,866,606 |
| TSU-02 | AIS | B | 3,191 | 1,809,512 | 1,888,110 |
| TSU-05 | AIS | B | 3,895 | 1,354,063 | 1,849,471 |
| TSU-15 | AIS | B | 3,397 | 1,705,302 | 1,911,984 |
| TSU-14 | AIS | B | 4,072 | 1,345,003 | 1,889,449 |
| TSU-16 | AIS | B | 4,794 | 1,077,865 | 1,822,595 |
| AD09-058 | MIA | B | 3,982 | 1,408,487 | 1,911,053 |
| TSU-27 | MIA | C | 5,974 | 912,762 | 1,852,755 |
| TSU-30 | MIA | C | 7,909 | 691,616 | 1,834,696 |
| TSU-31 | MIA | C | 4,785 | 1,056,118 | 1,813,757 |
| TSU-32 | MIA | C | 7,735 | 669,632 | 1,782,724 |
| TSU-33 | MIA | C | 5,132 | 1,071,939 | 1,835,112 |
| TSU-37 | MIA | C | 5,623 | 987,402 | 1,853,347 |
| TSU-38 | MIA | C | 5,367 | 1,036,423 | 1,841,939 |
| TSU-41 | MIA | C | 5,113 | 1,097,518 | 1,885,113 |
| TSU-36 | MIA | C | 8,113 | 666,380 | 1,848,815 |

| | | | | | |
|---|---|---|---|---|---|
| TSU-40 | MIA | C | 5,098 | 1,065,710 | 1,845,203 |
| TSU-28 | MIA | C | 7,277 | 749,095 | 1,833,530 |
| TSU-39 | MIA | C | 7,770 | 684,519 | 1,841,086 |
| TSU-29 | MIA | C | 7,234 | 761,125 | 1,836,202 |
| TSU-34 | MIA | C | 5,722 | 976,256 | 1,854,351 |
| L07K233 | Lepidic-Ad | C | 3,121 | 1,667,288 | 1,841,988 |
| L07K125 | Lepidic-Ad | C | 3,187 | 1,725,303 | 1,889,258 |
| Average (Early-Ad) | | | 5,051 | 1,160,572 | 1,846,039 |
| AD08_279 | Advanced-Ad | *** | 3,318 | 1,727,036 | 1,909,697 |
| 42 | Advanced-Ad | *** | 2,348 | 2,282,128 | 1,844,531 |
| L07K172 | Advanced-Ad | *** | 2,423 | 2,349,885 | 1,908,261 |
| AD09-116 | Advanced-Ad | *** | 2,966 | 1,800,665 | 1,857,754 |
| AD09-115 | Advanced-Ad | *** | 7,275 | 701,908 | 1,857,996 |
| Average (Advanced-Ad)* | | | 3,666 | 1,772,324 | 1,875,648 |

*The results of the other 20 cases (Advanced-Ad and Others) were previously reported[1,2].

**Supplementary Table S10 General statistics of target captured sequencing**

| Case | Classification | Noguchi type | Total | Depth |
|---|---|---|---|---|
| TSU-03 | AIS | A | 40,336,274 | 3,152 |
| TSU-06 | AIS | A | 32,203,911 | 2,632 |
| TSU-11 | AIS | B | 33,505,805 | 2,933 |
| TSU-01 | AIS | B | 34,802,939 | 3,044 |
| TSU-17 | AIS | B | 31,908,286 | 3,046 |
| TSU-02 | AIS | B | 41,068,769 | 3,140 |
| TSU-05 | AIS | B | 37,757,578 | 2,458 |
| TSU-15 | AIS | B | 32,784,974 | 2,560 |
| TSU-14 | AIS | B | 27,901,230 | 2,269 |
| TSU-16 | AIS | B | 32,441,797 | 2,930 |
| AD09-058 | MIA | B | 30,354,690 | 2,338 |
| L07K233 | Lepidic-Ad | C | 31,749,163 | 3,262 |
| L07K125 | Lepidic-Ad | C | 31,124,478 | 2,465 |
| AD08_279 | Advanced-Ad | *** | 32,139,598 | 2,621 |
| 42 | Advanced-Ad | *** | 29,023,660 | 2,362 |
| L07K172 | Advanced-Ad | *** | 28,836,229 | 2,113 |
| AD09-116 | Advanced-Ad | *** | 24,425,781 | 2,251 |
| AD09-115 | Advanced-Ad | *** | 21,417,537 | 2,617 |
| S10 | Advanced-Ad | *** | 22,000,328 | 2,445 |
| S8 | Advanced-Ad | *** | 24,949,870 | 2,367 |
| S6 | Advanced-Ad | *** | 24,091,125 | 2,435 |
| S20 | Advanced-Ad | *** | 26,062,568 | 2,837 |
| S19 | Advanced-Ad | *** | 25,184,536 | 2,692 |
| S2 | Advanced-Ad | *** | 25,179,015 | 2,344 |
| S13 | Advanced-Ad | *** | 24,360,134 | 2,568 |
| S21 | Advanced-Ad | *** | 21,591,371 | 2,245 |
| S7 | Advanced-Ad | *** | 26,245,224 | 2,810 |
| S5 | Advanced-Ad | *** | 27,075,142 | 2,848 |
| S15 | Advanced-Ad | *** | 27,705,348 | 2,891 |
| S3 | Advanced-Ad | *** | 25,632,648 | 2,538 |
| S17 | Advanced-Ad | *** | 22,179,933 | 2,484 |
| S14 | Others | *** | 28,712,781 | 2,897 |

| | | | | |
|---|---|---|---|---|
| S11 | Others | *** | 26,575,451 | 2,888 |
| S12 | Others | *** | 24,353,155 | 2,498 |
| S1 | Others | *** | 24,110,920 | 2,605 |
| S16 | Others | *** | 24,090,469 | 2,690 |

## Supplementary References


1. Sakamoto, Y. *et al.* Long-read sequencing for non-small-cell lung cancer genomes. *Genome Res.* **30**, 1243–1257 (2020).
2. Sakamoto, Y. *et al.* Phasing analysis of lung cancer genomes using a long read sequencer. *Nat. Commun.* **13**, 1–17 (2022).
3. Zhu, J. *et al.* Delineating the dynamic evolution from preneoplasia to invasive lung adenocarcinoma by integrating single-cell RNA sequencing and spatial transcriptomics. *Exp. Mol. Med.* **54**, 2060–2076 (2022).
4. Dejima, H. *et al.* Immune evolution from preneoplasia to invasive lung adenocarcinomas and underlying molecular features. *Nat. Commun.* **12**, 1–11 (2021).
5. The Cancer Genome Atlas Research Network. Comprehensive molecular profiling of lung adenocarcinoma. *Nature* **511**, 543–550 (2014).
6. Ding, L. *et al.* Somatic mutations affect key pathways in lung adenocarcinoma. *Nature* **455**, 1069–1075 (2008).
7. Saito, M. *et al.* Gene aberrations for precision medicine against lung adenocarcinoma. *Cancer Sci.* **107**, 713–720 (2016).
8. Dogan, S. *et al.* Molecular epidemiology of EGFR and KRAS mutations in 3,026 lung adenocarcinomas: Higher susceptibility of women to smoking-related KRAS-mutant cancers. *Clin. Cancer Res.* **18**, 6169–6177 (2012).
9. Ohashi, K. *et al.* Characteristics of lung cancers harboring NRAS mutations. *Clin. Cancer Res.* **19**, 2584–2591 (2013).
10. Tate, J. G. *et al.* COSMIC: The Catalogue Of Somatic Mutations In Cancer. *Nucleic Acids Res.* **47**, D941–D947 (2019).
11. Ou, S. H. I. *et al.* HER2 Transmembrane Domain (TMD) Mutations (V659/G660) That Stabilize Homo- and Heterodimerization Are Rare Oncogenic Drivers in Lung Adenocarcinoma That Respond to Afatinib. *J. Thorac. Oncol.* **12**, 446–457 (2017).
12. Sheikine, Y. *et al.* BRAF in Lung Cancers: Analysis of Patient Cases Reveals Recurrent BRAF Mutations, Fusions, Kinase Duplications, and Concurrent Alterations . *JCO Precis. Oncol.* **2**, 1–15 (2018).
13. Arcila, M. E. *et al.* MAP2K1 (MEK1) mutations define a distinct subset of lung adenocarcinoma associated with smoking. *Clin. Cancer Res.* **21**, 1935–1943 (2015).
