# Supplementary material for: Whole-genome sequencing reveals the molecular implications of the stepwise progression of lung adenocarcinoma
Source: Nat Commun. 2023 Dec 15;14:8375. doi: 10.1038/s41467-023-43732-y (PMC10724178; doi:10.1038/s41467-023-43732-y)
Supplement: Supplementary file 3 — Description of Additional Supplementary Files [file 41467_2023_43732_MOESM3_ESM.pdf]

Description of Additional Supplementary Files

Supplementary Data S1: Differentially expressed genes in RNA-seq data among the histological class

Supplementary Data S2: Differentially expressed genes in each cluster of Visium data

Supplementary Data S3: List of target genes in the human lung panel of Xenium
