# Supplementary material for: Whole-genome sequencing reveals the molecular implications of the stepwise progression of lung adenocarcinoma
Source: Nat Commun. 2023 Dec 15;14:8375. doi: 10.1038/s41467-023-43732-y (PMC10724178; doi:10.1038/s41467-023-43732-y)
Supplement: Supplementary file 5 — Reporting Summary [file 41467_2023_43732_MOESM5_ESM.pdf]

nature portfolio

Corresponding author(s): Yutaka Suzuki, Takashi Kohno, Ayako Suzuki

Last updated by author(s): Oct 31, 2023

Reporting Summary

Nature Portfolio wishes to improve the reproducibility of the work that we publish. This form provides structure for consistency and transparency in reporting. For further information on Nature Portfolio policies, see our Editorial Policies and the Editorial Policy Checklist.

Statistics

For all statistical analyses, confirm that the following items are present in the figure legend, table legend, main text, or Methods section.

n/a	Confirmed	
☐	☒	The exact sample size (*n*) for each experimental group/condition, given as a discrete number and unit of measurement
☐	☒	A statement on whether measurements were taken from distinct samples or whether the same sample was measured repeatedly
☐	☒	The statistical test(s) used AND whether they are one- or two-sided<br>*Only common tests should be described solely by name; describe more complex techniques in the Methods section.*
☐	☒	A description of all covariates tested
☐	☒	A description of any assumptions or corrections, such as tests of normality and adjustment for multiple comparisons
☐	☒	A full description of the statistical parameters including central tendency (e.g. means) or other basic estimates (e.g. regression coefficient) AND variation (e.g. standard deviation) or associated estimates of uncertainty (e.g. confidence intervals)
☐	☒	For null hypothesis testing, the test statistic (e.g. *F*, *t*, *r*) with confidence intervals, effect sizes, degrees of freedom and *P* value noted<br>*Give P values as exact values whenever suitable.*
☒	☐	For Bayesian analysis, information on the choice of priors and Markov chain Monte Carlo settings
☒	☐	For hierarchical and complex designs, identification of the appropriate level for tests and full reporting of outcomes
☐	☒	Estimates of effect sizes (e.g. Cohen's *d*, Pearson's *r*), indicating how they were calculated

Our web collection on statistics for biologists contains articles on many of the points above.

Software and code

Policy information about availability of computer code

Data collection

The methods for the data collection are described in the Methods section. The methods are also described as below;

Short read whole-genome sequencing:
Short read WGS was conducted at the two institutions. At the National Cancer Center in Japan, genomic DNA was extracted from frozen tumor tissues and paired normal counterparts using AllPrep DNA/RNA Mini Kit (Qiagen). According to the manufacturers' protocols, library preparation was conducted using TruSeq DNA PCR-Free Library Prep Kit (Illumina). At the University of Tokyo in Japan, genomic DNA was extracted using MagAttract HMW DNA Kit (Qiagen). According to the manufacturers' protocols, library preparation was conducted using TruSeq Nano DNA Library Prep kit (Illumina). Sequencing was performed by NovaSeq 6000 (Illumina).

Long read whole-genome sequencing:
From frozen tumor tissues and their normal counterparts, high molecular weight genomic DNA was extracted using MagAttract HMW DNA Kit (Qiagen). Library preparation was conducted using Ligation Sequencing Kit (SQK-LSK109 or SQK-LSK112/114, ONT) according to the manufacturer's protocol. Sequencing was performed by PromethION (ONT) with R9.4.1 (FLO-PRO002, ONT) or R10.2/10.4 (FLO-PRO112/FLO-PRO114, ONT) flow cells.

RNA-seq:
Total RNA was extracted from frozen tissues using AllPrep DNA/RNA Mini Kit (Qiagen). RNA-seq library preparation was conducted using TruSeq Stranded mRNA Library Prep (Illumina), TruSeq Stranded Total RNA Library Prep Gold (Illumina), or SMART-Seq Stranded Kit (Clontech Laboratories). Sequencing was performed by HiSeq 2000 system (Illumina).

Spatial transcriptome sequencing:

Fresh frozen tissues were sectioned at 10-µm thickness using a cryostat (CM1950, Leica). Methanol fixation and H&E staining were conducted according to the demonstrated protocol (CG000160, RevC, 10x Genomics). H&E imaging was performed using BZ-X800 (Keyence). Library construction was performed using Visium Spatial Gene Expression Slide & Reagent Kit (10x Genomics) according to the manufacturer's protocol (CG000239, RevF, 10x Genomics). Permeabilization time was set to 6 min. Sequencing was conducted by NovaSeq 6000 system (Illumina).

Multiplexed fluorescence immunostaining:
Fresh frozen tissue sections were prepared with Poly-L-Lysine-coated coverslips (CS04863, Matsunami) or MAS-coated coverslips (CS04883, Matsunami) at 10-µm thickness using a cryostat (CM1950, Leica). Multiplexed fluorescence immunostaining was conducted by PhenoCycler system (Akoya Biosciences) according to the manufacturer's protocol (CODEX User Manual – Rev C). To run a multicycle fluorescence detection, Keyence microscopy system (BZX800, Keyence) was used under the control by CODEX Instrument Manager (CIM, Akoya Biosciences). The output image data was visualized by QuPath (version 0.3.2).

In situ gene expression profiling
In situ RNA expression analysis at a subcellular level was performed by Xenium (10x Genomics). Fresh frozen tissue sections (10 µm thickness) were prepared on Xenium slides (10x Genomics) using a cryostat (CM1950, Leica). Fixation and tissue permeabilization were performed according to the protocol (CG000581, Rev A, 10x Genomics). Probe Hybridization Mix was prepared using a pre-designed panel (Xenium Human Lung Gene Expression panel, developmental build) and custom panel (Xenium Custom Gene Expression panel, design ID: 9GT3BT) according to the user guide (CG000582, Rev A, 10x Genomics). The prepared probes were hybridized at 50 °C overnight. Post-hybridization wash (37 °C for 30 min), ligation (37 °C for 2 h) and amplification (30 °C for 2 h) were performed according to the user guide. Autofluorescence quenching and nuclei staining were conducted in the dark. Using the prepared slide, fluorescent probe hybridization and imaging were conducted using the Xenium Analyzer (on-board analysis: version 1.1.0.2, software: version 1.1.2.4, 10x Genomics).

Target captured sequencing:
Using the extracted genomic DNA samples, target enrichment and library preparation were performed using SureSelect XT HS Reagents (cat# G9702C, Agilent Technologies) and SureSelect XT NCC oncopanel (cat# 931197, Agilent Technologies). The generated libraries were sequenced by NovaSeq 6000 system at 150-bp paired-end reads.

Data analysis

The softwares used for the data analysis are described in the Methods section. The code used in this study is available in the GitHub repository at https://github.com/asuzuki-asuzuki/Early-Ad_2023. The used softwares are also listed as below;

Detection of point mutations:
BWA-MEM (version 0.7.17), SAMtools (version 1.9), Picard (version 2.20.7) MarkDuplicates, GATK (version 4.1.3.0) Mutect2/FilterMutectCalls.

Detection of CNVs:
Control-FREEC (version 11.6).

Detection of SVs:
Minimap2 (version 2.17-r941), Nanomonsv (v0.5.0), GenomonSV (version 2.6.1), NCBI BLAST+ (version 2.13).

Mutational signature analysis:
SigProfilerExtractor (version 1.1.4).

DNA methylation analysis:
Nanopolish (version 0.13.2), guppy (version 6.2.1, ONT).

Gene expression analysis:
STAR (version 2.7.5c), fastp (version 0.23.2), Bowtie 2 (version 2.3.4.3), featureCounts (version 2.0.2), DESeq2 (version 1.32.0).

Analysis of spatial transcriptome data:
Space Ranger (version 1.3.1, 10x Genomics), Seurat (version v4.3.0), Giotto (version 1.1.2), CellChat (version 1.6.1).

Analysis of in situ gene expression data:
Seurat (version v4.3.0).

Deconvolution analysis:
CIBERSORTx (version 1.0), spacexr (version 2.2.1), scatterpie (version 0.1.9).

Phasing analysis:
GATK HaplotypeCaller (version 4.1.3.0), BCFtools (version 1.9), WhatsHap (version 1.1).

Haplotype assignments of somatic mutations and extraction of the order of mutation occurrences:
SAMtools (version 1.12) mpileup.

Estimation of clone architecture:
PyClone-VI (version 0.1.1), FACETS (version 0.6.2), snp-pileup (version 434b5ce), ClonEvol (version 0.99.11).

For manuscripts utilizing custom algorithms or software that are central to the research but not yet described in published literature, software must be made available to editors and reviewers. We strongly encourage code deposition in a community repository (e.g. GitHub). See the Nature Portfolio guidelines for submitting code & software for further information.

Data

Policy information about availability of data

All manuscripts must include a data availability statement. This statement should provide the following information, where applicable:

- Accession codes, unique identifiers, or web links for publicly available datasets
- A description of any restrictions on data availability
- For clinical datasets or third party data, please ensure that the statement adheres to our policy

The raw sequencing data including short read WGS, long read WGS, RNA-seq and spatial transcriptome data from the adenocarcinoma cases have been deposited in the Japanese Genotype-Phenotype Archive (JGA, http://trace.ddbj.nig.ac.jp/jga), which is hosted by the National Bioscience Database Center and DDBJ under accession code JGAS000570 [https://ddbj.nig.ac.jp/resource/jga-study/JGAS000570]. These data are available under restricted access due to ethical restriction. The raw sequencing data of short read WGS and long read WGS from the 20 advanced NSCLC cases that were previously obtained and reported were deposited in the DDBJ JGA with accession numbers JGAS000065 [https://ddbj.nig.ac.jp/resource/jga-study/JGAS000065] (JGAD000252 and JGAD000253) and JGAS000349 [https://ddbj.nig.ac.jp/resource/jga-study/JGAS000349], which are available under restricted access due to ethical restriction. These raw sequencing data are under restricted access because the Act on the Protection of Personal Information in Japan defines them as personally identifiable information. To access these data, users require the approval by the NBDC (https://humandbs.biosciencedbc.jp/en/guidelines/data-sharing-guidelines). The users can apply for the use of data via the application system (https://humandbs.biosciencedbc.jp/en/data-use). The restrictions for granting data are described in the following URL (https://humandbs.biosciencedbc.jp/en/guidelines/security-guidelines-for-users). The processed data was deposited in the database DBKERO (https://kero.hgc.jp/) and made publicly available in the download page (https://kero.hgc.jp/Early_cancer.html). The human reference genome hg38 was downloaded from the UCSC Genome Browser (https://hgdownload.soe.ucsc.edu/downloads.html). Source data of the figures are provided with this paper.

Research involving human participants, their data, or biological material

Policy information about studies with human participants or human data. See also policy information about sex, gender (identity/presentation), and sexual orientation and race, ethnicity and racism.

Reporting on sex and gender	We reported the number of cases for each sex in Table 1 of the manuscript.
Reporting on race, ethnicity, or other socially relevant groupings	We analyzed Japanese lung cancer patients.
Population characteristics	Japanese lung cancer patients.<br>We reported information of patients including age, smoking history tumor subtype and pathological stages in Table 1 and Supplementary Table S1.
Recruitment	All clinical samples were obtained with the appropriate informed consent by the National Cancer Center and University of Tsukuba, Japan.
Ethics oversight	National Cancer Center, Japan; University of Tsukuba, Japan; The University of Tokyo, Japan

Note that full information on the approval of the study protocol must also be provided in the manuscript.

Field-specific reporting

Please select the one below that is the best fit for your research. If you are not sure, read the appropriate sections before making your selection.

☒ Life sciences ☐ Behavioural & social sciences ☐ Ecological, evolutionary & environmental sciences

For a reference copy of the document with all sections, see nature.com/documents/nr-reporting-summary-flat.pdf

Life sciences study design

All studies must disclose on these points even when the disclosure is negative.

Sample size	No sample-size calculation was performed. Sample size was determined by the availability of the specimens and the sequencing data.
Data exclusions	No data were excluded from the analyses. For the RNA-seq data analyses (extraction of DEGs among stages and deconvolution), we used only the datasets from the poly-A capture method to avoid batch effect.
Replication	For some cases, the sequencing data cannot be reproduced because there is limitation for sample amounts of clinical specimens. For some cases with enough specimens, we validated driver mutations using target-captured sequencing.
Randomization	Randomization was not relevant to our study. Groups were determined by histological subtypes.
Blinding	Blinding was not relevant to our study using clinical specimens. Associations between the results of omics analyses and clinical information including histological subtypes in each case are important to understand biological relevance in this study.

Reporting for specific materials, systems and methods

We require information from authors about some types of materials, experimental systems and methods used in many studies. Here, indicate whether each material, system or method listed is relevant to your study. If you are not sure if a list item applies to your research, read the appropriate section before selecting a response.

Materials & experimental systems

n/a	Involved in the study
☐	☒ Antibodies
☒	☐ Eukaryotic cell lines
☒	☐ Palaeontology and archaeology
☒	☐ Animals and other organisms
☒	☐ Clinical data
☒	☐ Dual use research of concern
☒	☐ Plants

Methods

n/a	Involved in the study
☒	☐ ChIP-seq
☒	☐ Flow cytometry
☒	☐ MRI-based neuroimaging

Antibodies

Antibodies used

We used antibodies for multiplexed fluorescence immunostaining analysis by PhenoCycler (Akoya Biosciences). All antibodies are available to purchase. Information of all antibodies including clone names and catalogue numbers is shown in Supplementary Table S9.
Anti-CD19 (HIB19, cat# 4550099), anti-CD8 (SK1, cat# 4150004), anti-LIF (M1506B09, cat# 4450032), anti-CD279/PD-1 (EH12.2H7, cat# 4250010), anti-CD68 (KP1, cat# 4350019), anti-CD45RO (UCHL1, cat# 4250023), anti-Pan-Cytokeratin (AE-1/AE-3, cat# 4150020), anti-β-Catenin1 (12F7, cat# 4450036), anti-CD4 (SK3, cat# 4350010), anti-Podoplanin (NC-08, cat# 4250004), anti-CD11c (S-HCL-3, cat# 4550107), anti-CD31 (WM59, cat# 4250009), anti-Mac2/Galectin-3 (M3/38, cat# 4450034), anti-Ki67 (B56, cat# 4250019) and anti-Caveolin (D46G3, cat# 4550084) were purchased as conjugated-antibodies (PhenoCycler invetory, Akoya Biosciences).
Anti-CD163 (D6U1J, cat# 25121, CST), anti-BCA1/CXCL13 (EPR23400-92, cat# ab270408, Abcam), anti-CTLA4 (E2V1Z, cat# 26893, CST), anti-CD274/PD-L1 (E1L3N, cat# 85164, CST), anti-TTF-1/NKX2-1 (SP141, cat# ab242428, Abcam), anti-FOXP3 (236A/E7, cat# 14-4777-82, Invitrogen) and anti-α-SMA/ACTA2 (1A4, cat# ab7817, Abcam) were purchased from the corresponding suppliers for custom conjugation of PhenoCycler oligos.

Validation

Information of each antibody is provided by the manufacturers' web page. We reported information of manufacturers and catalogue numbers of antibodies in Supplementary Table S9 and above.
For conjugated-antibodies, information is provided in the Akoya Biosciences web page [https://www.akoyabio.com/phenocycler/assays/].
For other antibodies, information is provided as below;
anti-CD163 (D6U1J, cat# 25121, CST) [https://www.cellsignal.com/products/primary-antibodies/cd163-d6u1j-rabbit-mab-bsa-and-azide-free/25121];
anti-BCA1/CXCL13 (EPR23400-92, cat# ab270408, Abcam) [https://www.abcam.co.jp/products/primary-antibodies/cxcl13-antibody-epr23400-92-bsa-and-azide-free-ab270408.html];
anti-CTLA4 (E2V1Z, cat# 26893, CST) [https://www.cellsignal.jp/products/primary-antibodies/ctla-4-e2v1z-rabbit-mab-bsa-and-azide-free/26893];
anti-CD274/PD-L1 (E1L3N, cat# 85164, CST) [https://www.cellsignal.com/products/primary-antibodies/pd-l1-e1l3n-xp-rabbit-mab-bsa-and-azide-free/85164];
anti-TTF-1/NKX2-1 (SP141, cat# ab242428, Abcam) [https://www.abcam.co.jp/products/primary-antibodies/ttf1-antibody-sp141-bsa-and-azide-free-ab242428.html];
anti-FOXP3 (236A/E7, cat# 14-4777-82, Invitrogen) [https://www.thermofisher.com/antibody/product/FOXP3-Antibody-clone-236A-E7-Monoclonal/14-4777-82];
anti-α-SMA/ACTA2 (1A4, cat# ab7817, Abcam) [https://www.abcam.co.jp/products/primary-antibodies/alpha-smooth-muscle-actin-antibody-1a4-ab7817.html].
